# Integrated Mechanisms of Polarity–Based Extracts of *Cucumis melo* L. Seed Kernels for Airway Smooth Muscle Relaxation via Key Signaling Pathways Based on WGCNA, In Vivo, and In Vitro Analyses

**DOI:** 10.3390/ph15121522

**Published:** 2022-12-07

**Authors:** Muqeet Wahid, Fatima Saqib, Anam Ali, Abdulrahman Alshammari, Metab Alharbi, Abdur Rauf, Mohammad S. Mubarak

**Affiliations:** 1 Department of Pharmacology, Faculty of Pharmacy, Bahauddin Zakariya University, Multan 60000, Pakistan; 2 Department of Pharmacology and Toxicology, College of Pharmacy, King Saud University, Post Box 2455, Riyadh 11451, Saudi Arabia; 3 Department of Chemistry, University of Swabi, Swabi 94640, Pakistan; 4 Department of Chemistry, The University of Jordan, Amma 11942, Jordan; 5 Department of Chemistry, Indiana University, Bloomington, IN 47405, USA

**Keywords:** *Cucumis melo*, asthma, airway smooth muscle, WGCNA, LC/ESI–MS/MS, calcium–mediated smooth muscle contraction

## Abstract

The present study aimed to determine the mechanisms responsible for calcium–mediated smooth muscle contractions in *C. melo* seeds. The phytochemicals of *C. melo* were identified and quantified with the aid of Liquid Chromatography Electrospray Ionization Tandem Mass Spectrometric (LC/ESI–MS/MS) and high–performance liquid chromatography (HPLC), and then tested in–vitro and in vivo to confirm involvement in smooth muscle relaxation. Allergic asthma gene datasets were acquired from the NCBI gene expression omnibus (GEO) and differentially expressed gene (DEG) analysis, weighted gene co–expression network analysis (WGCNA), and functional enrichment analysis were conducted. Additionally, molecular docking of key genes was carried out. Kaempferol, rutin, and quercetin are identified as phytochemical constituents of *C. melo* seeds. Results indicated that *C. melo* seeds exhibit a dose–dependent relaxant effect for potassium chloride (80 mM)– induced spastic contraction and calcium antagonistic response in calcium dose–response curves. The functional enrichment of WGCNA and DEG asthma–associated pathogenic genes showed cytokine–mediated pathways and inflammatory responses. Furthermore, CACNA1A, IL2RB, and NOS2 were identified as key genes with greater binding affinity with rutin, quercitrin, and kaempferol in molecular docking. These results show that the bronchodilator and antidiarrheal effects of *C. melo* were produced by altering the regulatory genes of calcium–mediated smooth muscle contraction.

## 1. Introduction

Asthma is a chronic inflammatory disorder of the airways involving airflow obstruction and underlying inflammation. Its clinical symptoms depend on allergic status, age, and environmental triggering factors [1]. It affects about 300 million people globally, including 25 million Americans [1]. Asthma is characterized by severe airway structural alterations, including epithelial lining loss, hyperplasia in goblet cells, thickening of the basement membrane of subepithelial reticular, and enlargement of airway smooth muscle mass, contributing to airflow obstruction [2]. Airway hyper–responsiveness (AHR) is another clinical feature with increased sensitivity to inhaled constrictor agonists, resulting in stronger airway constriction in response to environmental stimuli [3]. It also shows an association with the histamine cytokines, i.e., interleukin–13 (IL–13), interleukin–4 (IL–4), and interleukin–5 (IL–5), which stimulate the formation of the IgE and eosinophilia in the airways as well as mucus and bronchial hyperresponsiveness [4].

Diarrhea is the hallmark symptom associated with inflammatory bowel disease (IBD) and is observed in approximately 80% of cases. The diarrhea–predominant disease displays more–rapid gastrointestinal (GI) transit times. In this respect, the autonomic nervous system mediates brain–gut communication, and coordinates and modulates GI motility, secretion, and immune function. Increased sympathetic nervous activity and decreased parasympathetic activity are essential to responding to stress [5]. Mechanistically, an imbalance in electrolyte absorption and secretion in the intestine disrupts the osmotic gradient, resulting in water retention in the lumen and diarrhea. The severity of diarrhea (stool frequency and consistency) is considered an essential determinant of the disease activity index [6,7]. Bladder smooth muscles control the two primary functions of the bladder: storage of urine and voiding. They include muscular bundles that contract to facilitate urination and relax to facilitate storage [8].

*Cucumis melo Linn* belongs to the family Cucurbitaceae; its common name is muskmelon [9]. It is extensively cultivated worldwide as an edible fruit or vegetable [10,11]. *C. melo* is a nutritious and therapeutic fruit with a delicious taste. Due to its high nutritional value, *C. melo* fruit can be consumed fresh, canned, or processed. The pulp of *C. melo* is commonly eaten in desserts, in salads, raw, or roasted [11,12]. The fruit is a rich source of amino acids, proteins, carbohydrates, vitamins A and C along with B1 and B2, pyridoxine, niacin, and folate in smaller quantities, minerals including calcium, iron, potassium, phosphorus, magnesium, and sodium [13,14,15,16]. Melons contain various fatty acids, polyphenols, and carotenoids that have several health benefits. Several chronic diseases, including, inflammation, and certain types of cancer, can be treated and prevented by consuming melon as a natural curative agent [17]. *C. melo* has been used in traditional medicines for centuries in India, Pakistan, Iran, and China for gastrointestinal, circulatory, neurological, and urogenital problems [18,19,20,21,22]. Accordingly, this study aims to determine whether *C. melo* seed kernels have antispasmodic properties and identify how calcium–mediated signaling pathways control smooth muscle contraction genes.

## 2. Results

### 2.1. Identification of Bioactive Compounds

Twenty phytoconstituents were identified in the LC/ESI–MS/MS study of *C. melo* seed kernel sequential extracts. Table S1 lists the fragmentations of the major phytochemicals responsible for the biological activities.

### 2.2. Method Validation and Parameter Optimization for HPLC

We adjusted HPLC settings to achieve maximum separation and noticeable peaks in the *C. melo* seed kernel sequential extracts. Separation of phytoconstituents was performed using a binary mobile–phase system with an 0.8 mL/min flow rate. Different wavelengths of 320, 280, and 270 nm were used to identify separation peaks for *C. melo* seed kernel sequential extracts. In the next step, the peaks were compared to external standards (Table 1 and Figure S1). Table 1 lists the separation peaks for phytochemicals from sequential extracts at wavelengths of 270, 280, and 320 nm. 

### 2.3. Quantification of Phytochemical Compounds

Using linear regression, phytoconstituents were quantified by comparing them to external standards. Table 1 lists the quantification of phytoconstituent in *C. melo* seed kernel sequential extracts.

### 2.4. Isolated Tissue Experimentation

#### 2.4.1. Effects on Isolated Rabbit Jejunum Preparation

Seed kernel extracts of *C. melo* were tested for their effects on spontaneous spasms in jejunal preparations. Despite the ethanol extract displaying a spasmogenic contractile response, all subsequent extracts had a spasmolytic effect and inhibited spasms evoked by potassium chloride (K^+^; 80 and 25 mM). A dose–dependent inhibition of spontaneous rhythmic spasms was found at 0.3 and 1 mg/mL for *n*–hexane (Cm–hexane), DCM (Cm–DCM), and aqueous (Cm–aqueous) extracts of *C. melo* seed kernels. The EC_50_ was 0.1767 mg/mL (0.1244 to 0.2636; 95% CI), 0.2490 mg/mL (0.1873 to 0.3344; 95% CI), and 4.662 mg/mL (2.125 to 16.28; 95% CI), respectively. In contrast, ethanol (Cm–ethanol) extract of *C. melo* seed kernels did induce a contractile response in a jejunal preparation that was reduced by pretreatment with atropine (1 µM). An effective relaxation was observed at 1 mg/mL dose with an EC_50_ of 0.5218 (0.3858 to 0.7317; 95% CI). Cm–hexane relaxed the spastic spasms of potassium chloride (80 mM), Cm–DCM, Cm–ethanol, and Cm–aqueous at doses of 0.3, 1, 3, and 3 mg/mL, with EC_50_ of 0.2437 (0.1636 to 0.3969; 95% CI), 0.3128 (0.2484 to 0.3982; 95% CI), 2.089 (1.218 to 4.141; 95% CI), and 0.5439 mg/mL (0.4209 to 0.7075; 95% CI). All extracts relaxed the potassium chloride (25 mM)–induced spasms at 0.1, 0.3, 1, and 0.3 mg/mL with EC_50_ of 0.1112 (0.08209 to 0.1532; 95% CI), 0.1926 (0.1281 to 0.2919; 95% CI), 1.076 (0.8378 to 1.402; 95% CI), and 0.2654 (0.1822 to 0.3938; 95% CI), respectively (Figure S2, Table 2).

In both the absence and presence of *C. melo* seed kernel sequential extracts, the antagonistic response of calcium ion channels was determined, and the calcium DRCs were produced in cytosolic calcium–free jejunal preparation. However, Cm–hexane suppressed calcium contractile response DRCs only at 0.3 and 1 mg/mL, whereas all other extracts significantly suppressed calcium DRCs at 3 mg/mL. Although cholinomimetic bioactive substances may affect or alter Cm–ethanol effects, calcium DRCs in atropine–pretreated tissue displays a rightward shift with suppression of DRCs during atropine pretreatment [23]. Atropine showed no effect on calcium DRCs [24]. On the other hand, verapamil was used to confirm these findings of *C. melo* seed kernel sequential extracts. Verapamil reduced spontaneous contractions of jejunal preparations, potassium chloride (80 and 25 mM)–elicited spasms at corresponding doses of 0.1, 1, and 0.1 µM with EC_50_ of 0.04725 (0.03764 to 0.05968; 95% CI), 0.1194 (0.09889 to 0.1445; 95% CI), and 0.01595 µM (0.01303 to 0.01956; 95% CI). Additionally, calcium DRCs were constructed, resulting in calcium DRCs blocked at a dose of 0.3 µM equivalent to *C. melo* seed kernel sequential extracts, indicating an antagonistic effect towards the calcium channels (Figure S2, Table 2).

#### 2.4.2. Effect of Cm–Ethanol on Isolated Rat Ileum Preparations

**Figure 1:**
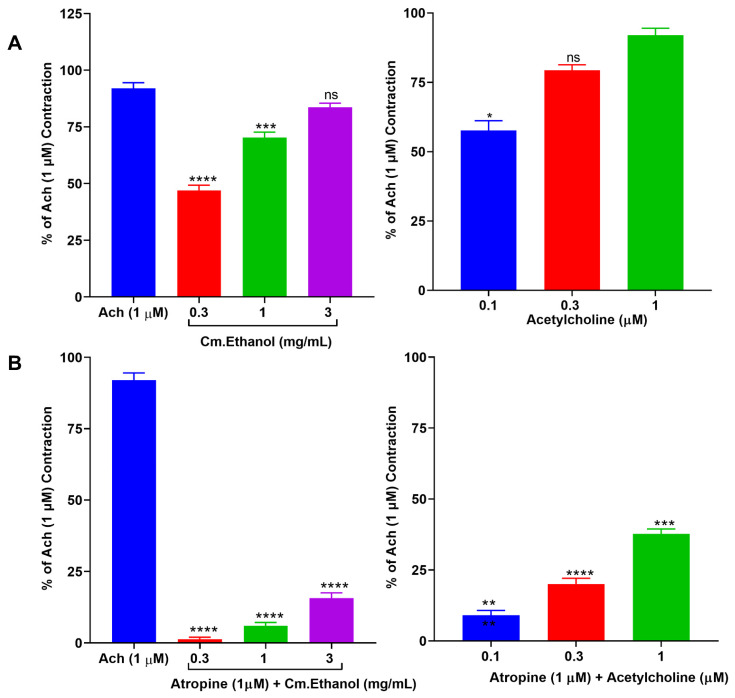
Cm–ethanol and acetylcholine (Ach) were tested in the (**A**) absence and the (**B**) presence of atropine in the ileum preparations of rats. Data are expressed as the mean ± S.D. (*n* = 4). A Student’s *t*–test was performed to determine significance when compared to Ach: *p* < 0.05, * *p* < 0.01, ** *p* < 0.001, *** *p* < 0.0001, **** *p* < 0.00001 were used to determine significance.

The bolus doses of Cm–ethanol were added to isolated rat ileum to induce spasms. We found that Cm–ethanol elicited more–significant contractile responses than acetylcholine (0.1, 0.3, and 1 µM) at 0.3, 1, and 3 mg/mL. With the presence of atropine (1 µM), Cm–ethanol spasms were decreased to a similar extent as those of Ach–induced spasm inhibition (Figure 1, Table 2).

#### 2.4.3. Effect on Isolated Rabbit Tracheal Preparations

Sequential extracts of *C. melo* seed kernels were tested in rabbit tracheal preparations for their bronchodilator properties. The dilatory effect of *C. melo* seed kernel sequential extracts was dose–dependently induced by spastic spasms of potassium chloride (80 and 25 mM) and CCh (1 µM). Cm–hexane, Cm–DCM, Cm–ethanol, and Cm–aqueous relaxed spastic spasms of potassium chloride (80 mM) at doses of 3, 5, 10, and 5 mg/mL, with EC_50_ of 0.9567 (0.6366 to 1.482; 95% CI), 2.596 (1.855 to 3.796; 95% CI), 4.055 (2.838 to 5.896; 95% CI), and 1.448 (1.186 to 1.783; 95% CI), respectively. Cm–hexane, Cm–DCM, Cm–ethanol, and Cm–aqueous relaxed CCh (1 µM)–elicited contraction at doses of 1, 3, 5, and 1 mg/mL, with EC_50_ of 0.5497 (0.4305 to 0.7191; 95% CI), 0.8250 (0.5890 to 1.176; 95% CI), 3.393 (2.357 to 5.184; 95% CI), and 0.1972 (0.1556 to 0.2510; 95% CI), respectively. However, Cm–hexane, Cm–DCM, Cm–ethanol, and Cm–aqueous relaxed the potassium chloride (25 mM)–induced spasms at doses of 0.3, 1, 3, and 1 mg/mL, with EC_50_ of 0.05417 (0.03680 to 0.07970; 95% CI), 0.7764 (0.5116 to 1.312; 95% CI), 1.726 (1.166 to 2.714; 95% CI), and 0.2748 (0.2116 to 0.3606; 95% CI), respectively (Figure S3, Table 2). Carbachol DRCs were formed in the absence and presence of Cm–DCM, Cm–ethanol, and Cm–aqueous (1 and 3 mg/mL), except Cm–hexane at doses of 0.3 and 1 mg/mL on tracheal preparation, and their suppression resulted in a non–competitive shift to the right. In tracheal preparations, verapamil inhibited spastic spasms of potassium chloride (80 and 25 mM) and carbachol (1 µM) in a dose–dependent manner at respective doses of 3, 0.3, and 0.1 µM with EC_50_ of 0.2564 µM (0.2111 to 0.3118; 95% CI), 0.06765 µM (0.05113 to 0.09052; 95% CI), and 0.01278 µM (0.01017 to 0.01608; 95% CI). Verapamil inhibited DRCs for carbachol at a dose of 1 µM, similar to *C. melo* seed kernel sequential extracts (Figure S3, Table 2).

#### 2.4.4. Effect on Isolated Rabbit Urinary Bladder Preparations

*C. melo* seed kernel sequential extracts were applied on potassium chloride (80 and 25 mM) and CCh (1 µM) to induce spastic spasms in urinary bladder preparations. Cm–hexane, Cm–DCM, Cm–ethanol, and Cm–aqueous exhibited dose–dependent relaxant responses. Potassium chloride (80 mM)–induced spasms were relaxed at doses of 3, 3, 5, and 1 mg/mL, with EC_50_ of 0.5107 (0.3928 to 0.6686; 95% CI), 1.124 (0.8837 to 1.451; 95% CI), 1.607 (1.293 to 2.022; 95% CI), and 0.2199 (0.1667 to 0.2924; 95% CI), respectively. Similarly, CCh (1 µM)–induced spasms were relaxed at doses of 1, 1, 3, and 0.3 mg/mL, with EC_50_ of 0.2540 (0.1859 to 0.3521; 95% CI), 0.4637 (0.3606 to 0.6092; 95% CI), 0.6436 (0.5133 to 0.8126; 95% CI), and 0.7115 (0.04493 to 0.1140; 95% CI), respectively. Furthermore, potassium chloride (25 mM)–induced spasms were relaxed at doses of 1, 3, 5, and 1 mg/mL, with EC_50_ of 0.4152 (0.3313 to 0.5281), 0.7650 (0.5168 to 1.161; 95% CI), 1.461 (1.185 to 1.821; 95% CI), and 0.1602 (0.1195 to 0.2154; 95% CI), respectively (Figure S4, Table 2). DRCs of calcium were produced in the absence and presence of *C. melo* seed kernel sequential extracts. Results showed that Cm–DCM and Cm–aqueous extracts suppress and shift the DRCs to the right at 1 and 3 mg/mL, whereas Cm–hexane suppresses with the rightward shift at 0.30 and 1.0 mg/mL. In contrast, Cm–ethanol extract shifted the calcium DRCs towards the right at doses of 3.0 and 5.0 mg/mL with no evident suppression. However, when pretreated with atropine, Cm–ethanol markedly suppressed calcium DRCs at 0.30 and 1.0 mg/mL. Verapamil reduced potassium chloride (80 and 25 mM)– and CCh (1 µM)–induced spasms at corresponding doses of 0.3, 1, and 0.1 µM with EC_50_ of 0.1036 µM (0.08172 to 0.1330; 95% CI), 0.1371 µM (0.1092 to 0.1723; 95% CI), and 0.009449 µM (0.007502 to 0.01187; 95% CI), respectively. Verapamil inhibited calcium DRCs at a concentration of 1 µM, similar to *C. melo* seed kernel sequential extracts (Figure S4, Table 2).

### 2.5. In Vivo Experiments

#### 2.5.1. Effect on GI Charcoal Meal Intestinal Transit

**Figure 2:**
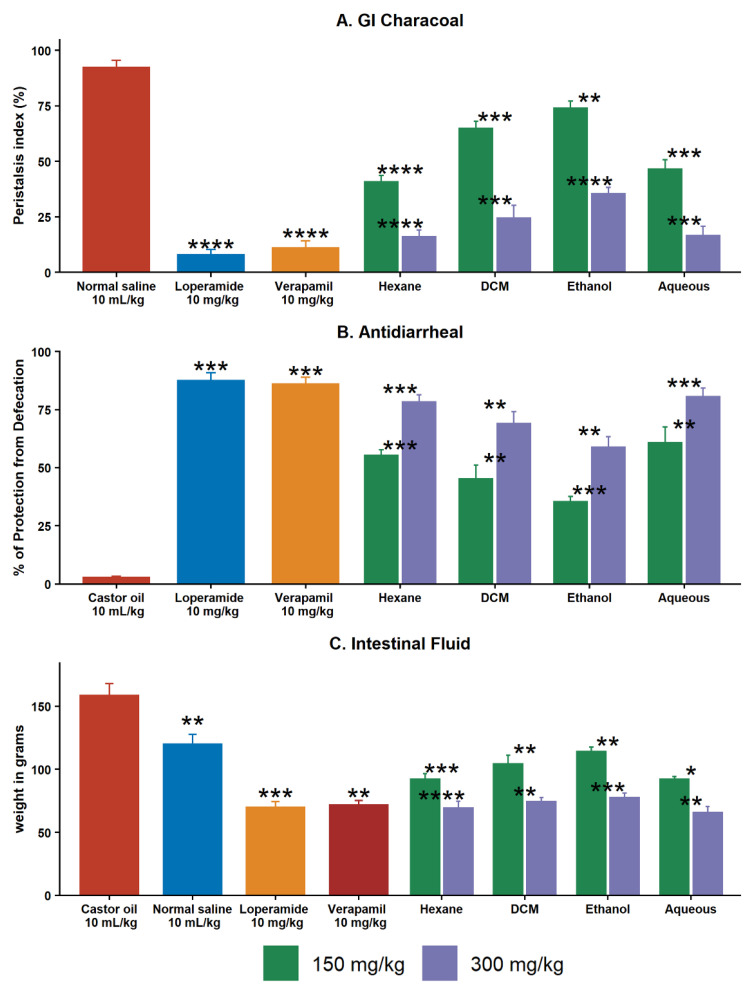
Gastrointestinal activities of in vivo activities of *C. melo* seed kernel sequential extracts. (**A**). Antiperistalsis, (**B**). antidiarrheal activity, and (**C**). GI fluid accumulation activity of verapamil, loperamide, and *C. melo* seed kernel sequential extracts. Data are expressed as the mean ± S.D. (*n* = 4). A Student’s *t*–test (pairwise) was performed to determine significance when compared to control: *p* < 0.05, * *p* < 0.01, ** *p* < 0.001, *** *p* < 0.0001, **** *p* < 0.00001 were used to determine significance.

Charcoal meal propulsive movements were reduced dose–dependently when *C. melo* seed kernel sequential extracts were pretreated (Figure 2). The peristalsis index of Cm–hexane, Cm–DCM, Cm–ethanol, and Cm–aqueous was 40.85 ± 2.8, 64.80 ± 3.3, 73.93 ± 3.3, and 46.54 ± 4.2% at doses of 150 and 16.00 ± 3.0, 24.53 ± 5.6, 35.38 ± 2.8, and 16.52 ± 4.2% at a dose of 300 mg/kg in comparison to loperamide (7.95 ± 2.3%) and verapamil (10.90 ± 3.3%) at a dose 10 mg/kg.

#### 2.5.2. Effect of Extracts on Castor Oil–Induced Diarrhea

A dose–dependent inhibitory effect was found for castor oil–induced diarrhea after the pretreatment of *C. melo* seed kernel sequential extracts (Figure 2). *C. melo* seed kernel sequential extracts protect the animals from defecation. The percentage of protection of Cm–hexane, Cm–DCM, Cm–ethanol, and Cm–aqueous at a dose of 150 mg/kg was 55.28 ± 2.5, 45.22 ± 5.9, 35.36 ± 2.3, and 71.91 ± 4.1% and at a dose of 300 mg/kg was 78.33 ± 3.05, 69.03 ± 5.1, 58.91 ± 4.5, and 80.62 ± 3.7% in comparison to loperamide (87.55 ± 3.3%) and verapamil (86.12 ± 2.9%) at a dose 10 mg/kg.

#### 2.5.3. Effect of Extracts on Intestinal Fluid Accumulation

Results from this investigation (Figure 2) indicated that, in contrast to the control group, castor oil treatment significantly increased gastrointestinal intestinal fluid accumulation (164.41 ± 2.6 g). On the other hand, *C. melo* seed kernel sequential extracts caused a dose–dependent significant (*p* < 0.001 vs. castor oil group) decrease in fluid in the intestine, with fluid weight 92.20 ± 4.4, 104.33 ± 6.6, 114.01 ± 3.7, and 91.96 ± 2.2 g, respectively, at the dose of 150, and 69.39 ± 5.10, 74.32 ± 3.40, 77.72 ± 3.40, and 65.77 ± 4.60 g at the dose of 300 mg/kg in comparison to loperamide (68.4 ± 4.3 g) and verapamil (73.65 ± 3.30 g).

### 2.6. WGCNA and DEG Studies

#### 2.6.1. Data Preprocessing and Standardization

**Figure 3:**
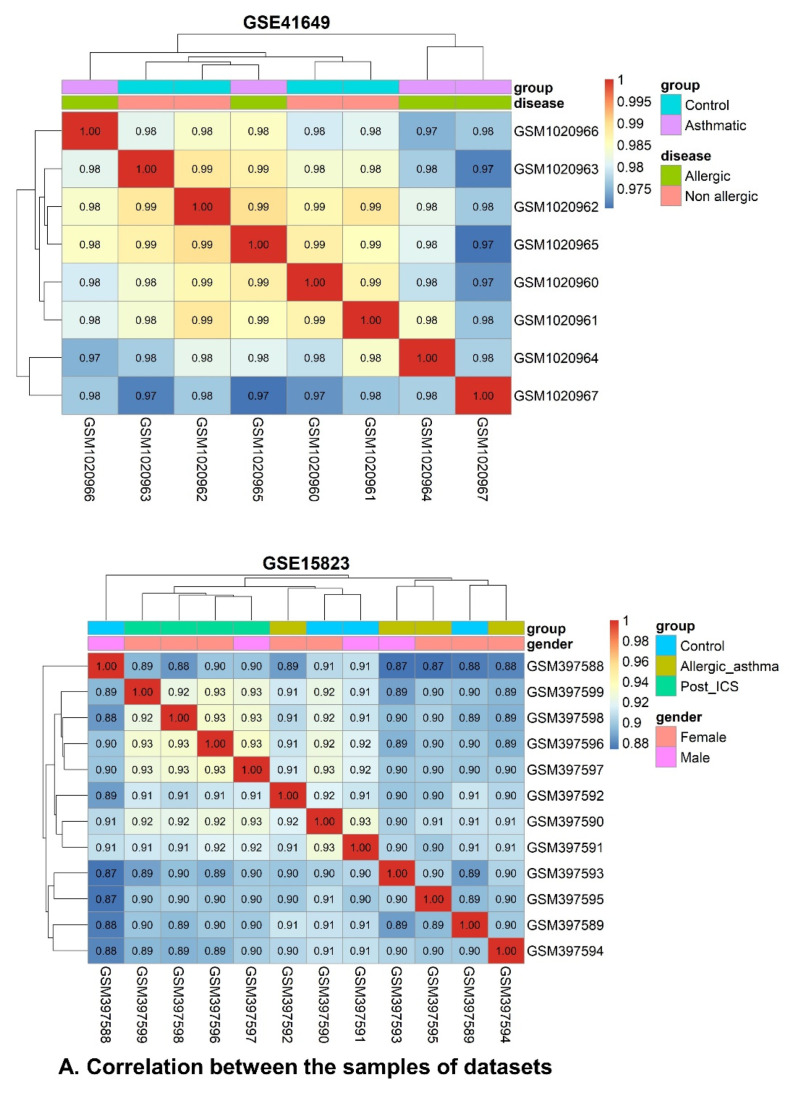

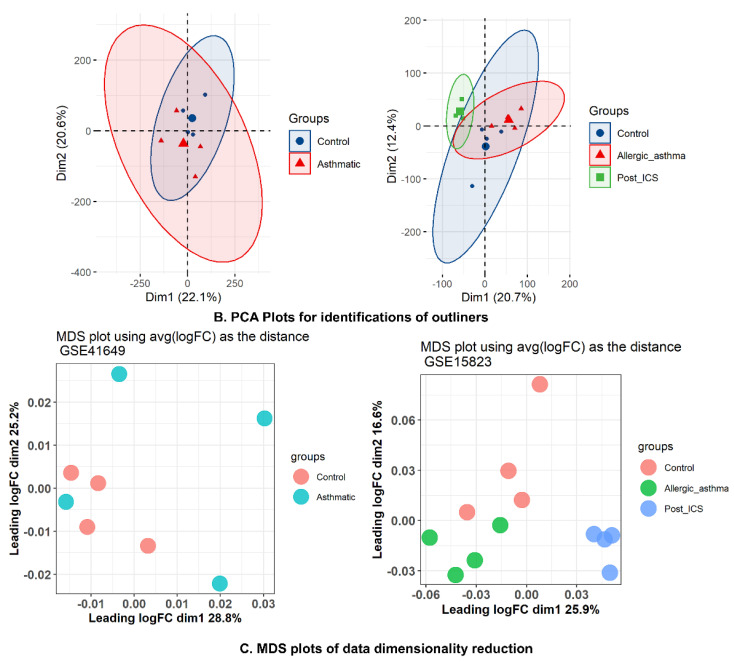
Standardization and preprocessing of datasets (GSE41649 and GSE15823) for WGCNA and DEG analysis. (**A**) Pearson correlation between datasets. (**B**) Principal component analysis (PCA) plot of datasets. (**C**) Dimension–reduction MDS plots for dissimilarities in datasets.

GSE41649 and GSE15823, two genes associated with asthma phenotype, were obtained from the GEO database. GSE41649 included four healthy controls and four allergic asthmatic participants. Control and Asthmatic research groups expressed a total of 22,283 genes in GSE14649. A total of 12,625 gene expressions were obtained from GSE15823, including three research groups (control, asthmatic, and post–inhaled corticosteroids (ICSs)). Following unsupervised analysis, pairwise correlation was used for scaling the data. For further analysis, 11,564 and 7265 gene expressions were retrieved for GSE41649 and GSE15823, respectively, after removing genes with low expression using the coefficient of variance (Figure 3).

#### 2.6.2. DEG Identification and Enrichment Analyses

**Figure 4:**
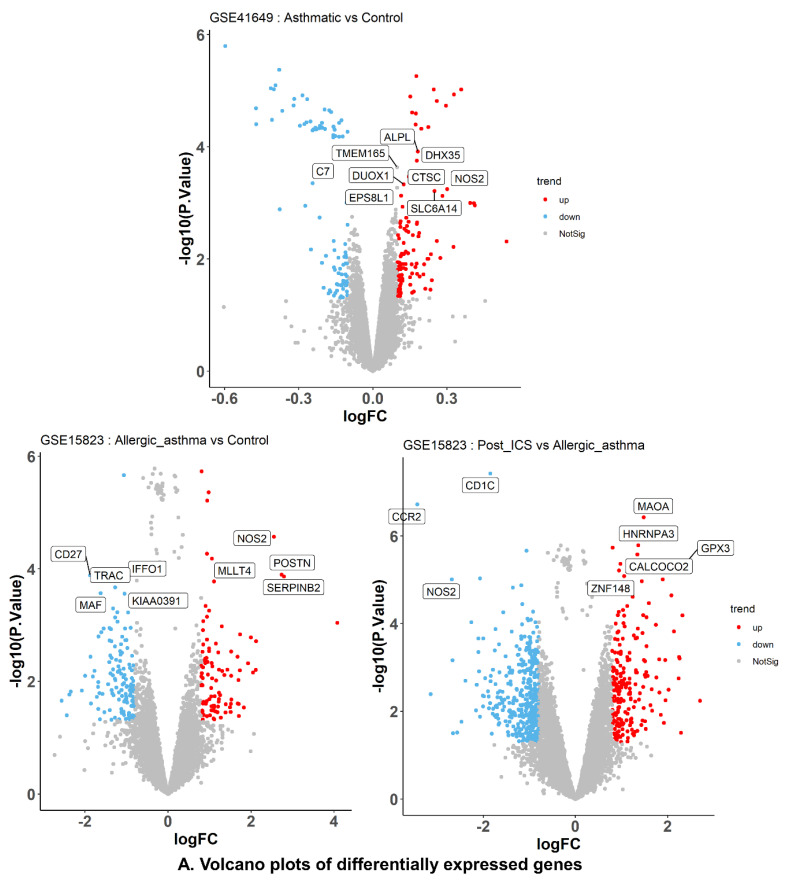

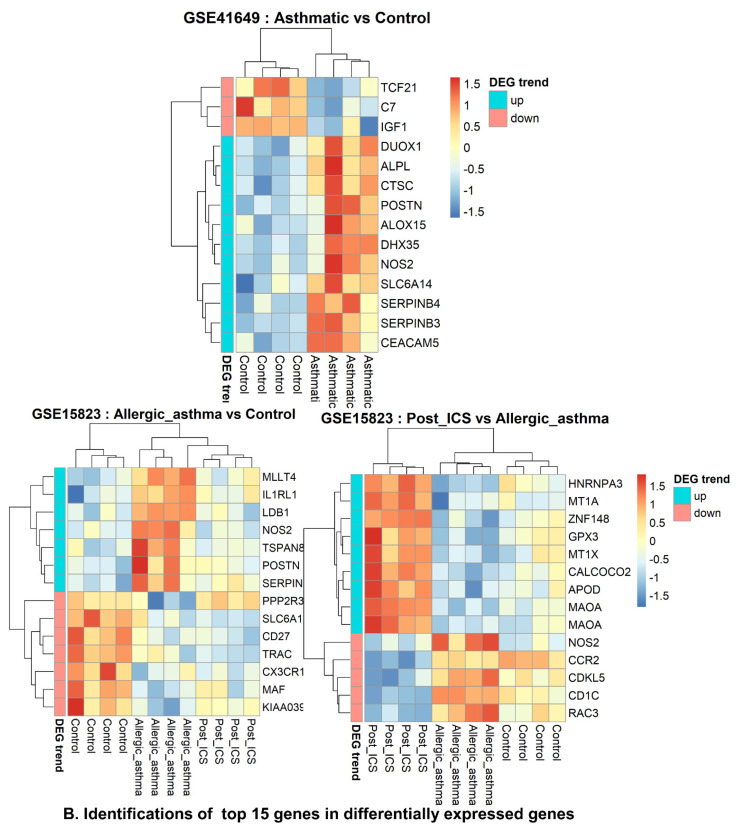
Contrast–dependent identification of differentially expressed genes (DEGs) of GSE41649 and GSE41649. (**A**) DEG volcano plots of datasets. (**B**) Top 15 DEGs of datasets. Contrasts: GSE41649: Asthma vs. control, GSE15283: Asthma vs. control, Post–ICS vs. asthma.

We analyzed GSE41649 and GSE15823 using Limma R for DEGs, identifying 202 and 223 important genes, respectively. Among the genes included in GSE41649, 109 exhibited upregulation, and 93 displayed downregulation (Figure 4A). The gene expression levels of 129 genes were downregulated, while 94 genes were upregulated in GSE15823. Following the identification of the genes with the most significant differences between GSE41649 and GSE15823, hierarchical clustering was conducted (Figure 4B).

Cluster–profiler R was also used to compare down– and upregulated genes in both datasets to determine GO and KEGG enrichment. For GSE41649 and GSE15283, Figure S5 shows the most significant GO BP and KEGG terms. GO BP terms for GSE41649 were calcium–mediated signaling, protein serine/threonine kinase activity, and inflammation regulation. In contrast, KEGG terms included inflammatory mediators of TRP channels, cholinergic synapse signaling, and smooth muscle contraction of vascular vessels. Active stimulation of glutamatergic, dopaminergic, and serotonergic synapses also induced inflammation, bronchoconstriction, and blood vessel contraction.

GSE15283 up– and downregulated genes were significantly enriched in GO BP related to calcium ion transmembrane transport, membrane depolarization, and second messenger signaling. The KEGG pathways associated with up– and downregulation include GnRH secretion, serotonergic synapses, cholinergic synapses, glutamatergic synapses, and the JAK–STAT signaling pathway.

#### 2.6.3. Identification of WGCNA Modules

**Figure 5:**
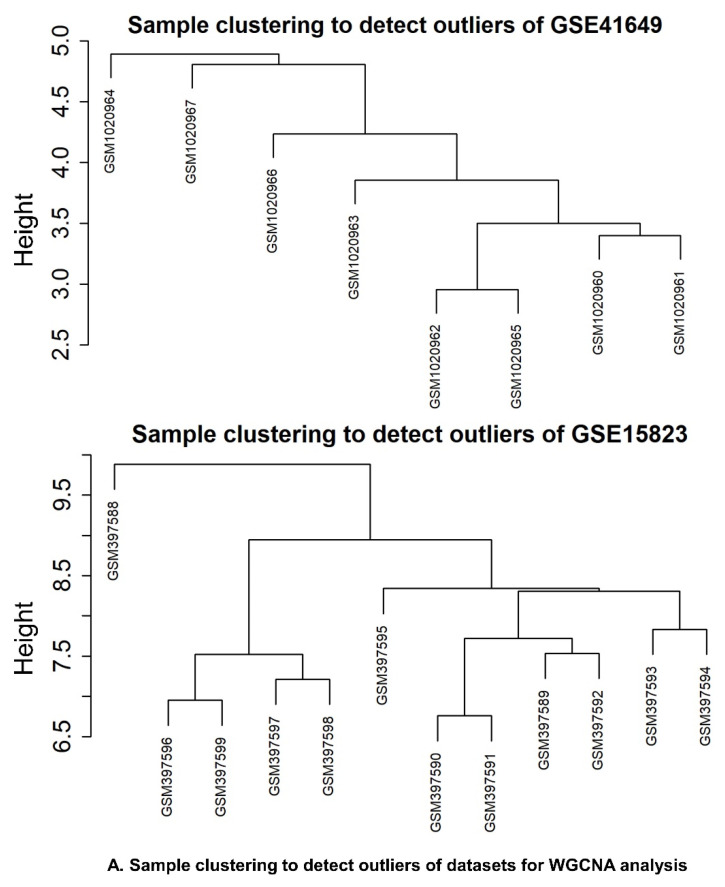

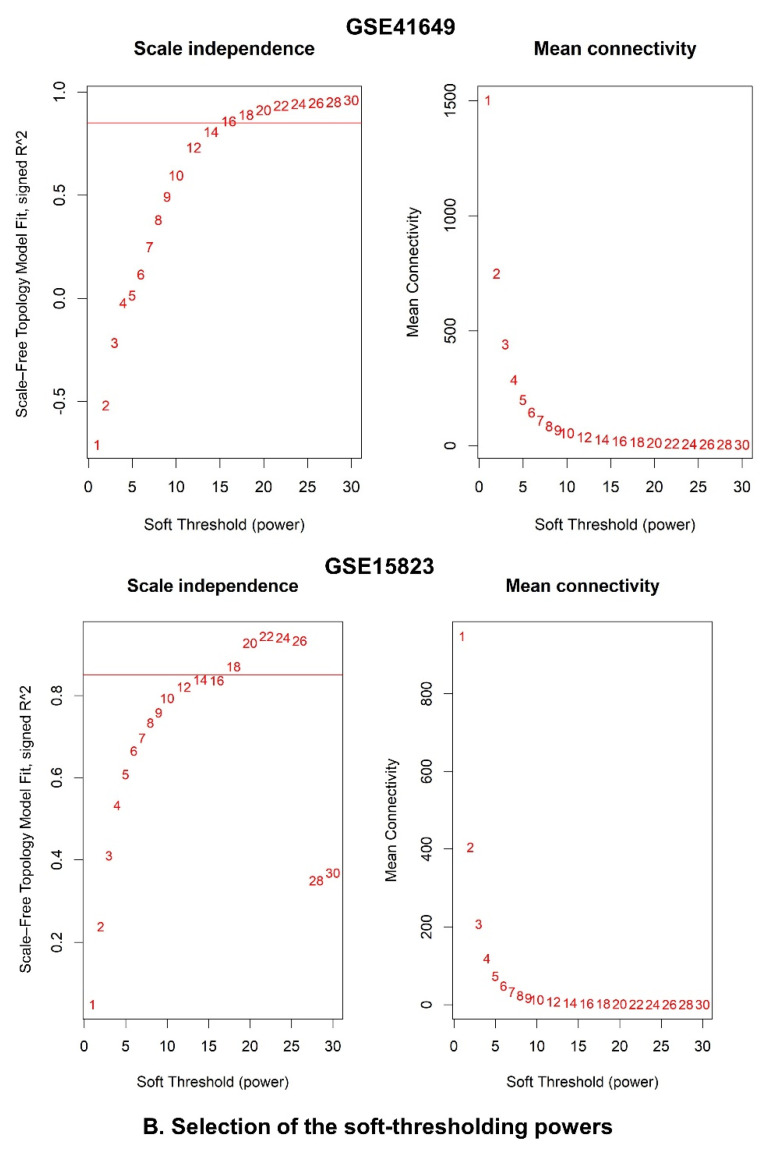
(**A**) The clustering of samples to identify outliners. (**B**) Scale–free fit index (**left**) and mean connectivity analysis (**right**) were used in the soft–threshold power–selection process.

An expression matrix was constructed based on GSE41649 and GSE15823 preprocessed data. A total of 4335 genes were included in the top 25% of variants for GSE41649 and 3256 genes for GSE15823 for WGCNA analysis. The cut height for GSE41649 and GSE15823 was set at 4.9 and 9.5, respectively, to omit the most noticeable outliers (Figure 5A). A gene expression matrix was created when GSE41649 and GSE15823 samples were standardized, background–corrected, and polymerized. To build a gene co–expression network, we chose a soft threshold of β = 16 to verify the attributes of the scale–free network when the fitting coefficient R2 approached 0.90 (Figure 5B).

**Figure 6:**
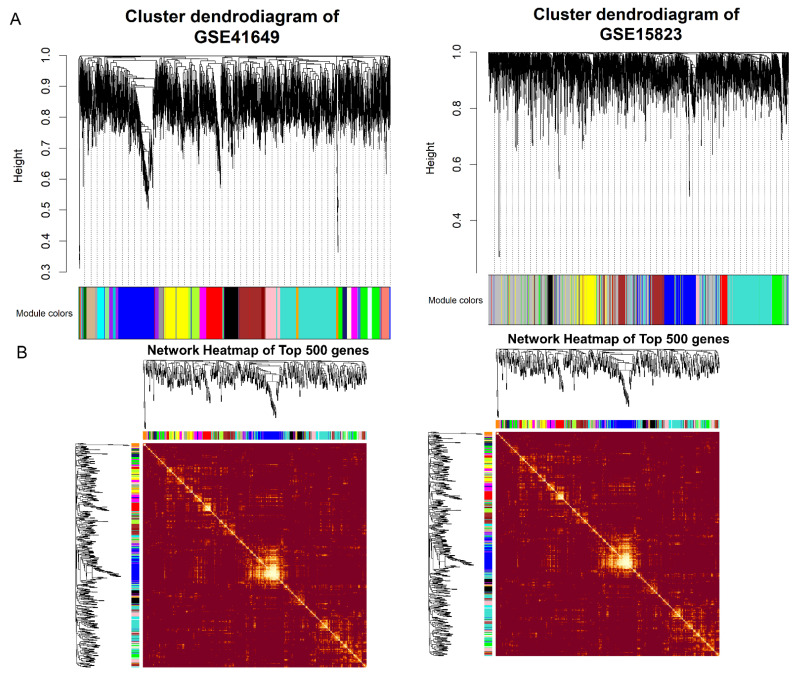
Module identification of weight gene co–expressed networks in datasets. (**A**) Cluster dendrogram and module assignment; the branches represent highly interconnected groups of genes. The horizontal bar shows the colors of the modules. (**B**) Heatmap of top 500 genes of weight gene co–expressed network.

A clustering dendrogram can be drawn by merging modules with high similarity using dynamic tree cutting and the hclust function. A clustering dendrogram (Figure 6A) was drawn by combining highly similar modules with dynamic tree cutting and hclust. A total of 27 gene modules were found for GSE41649, while 9 were for GSE15823.

#### 2.6.4. Correlation between Modules and Clinical Traits

**Figure 7:**
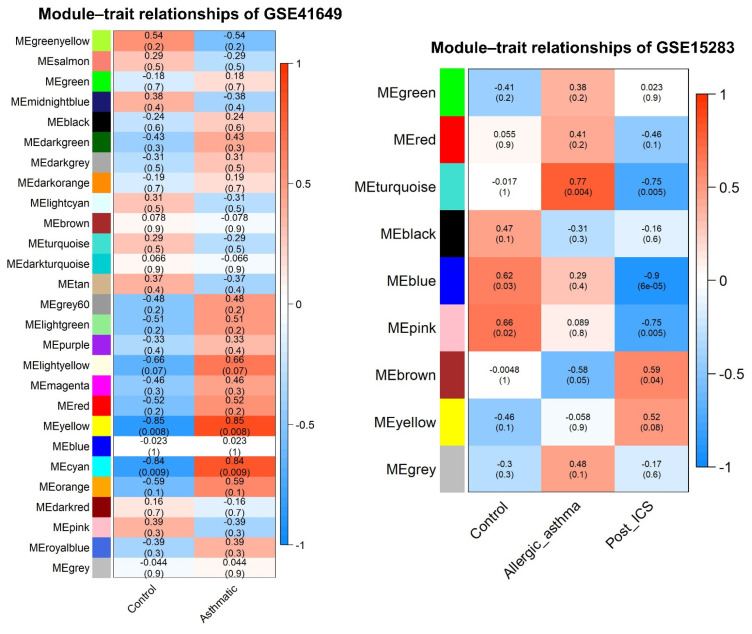
Pearson’s correlation heatmap between dataset groups and color modules.

The correlation results of GSE41649 and GSE15283 are illustrated in Figure 7, which shows the association between clinical features and module characteristics. According to the heatmap, modules that are positively correlated for control in GSE41649 were green–yellow (cor = 0.54; *p*–value = 0.17), pink (cor = 0.39; *p*–value = 0.34), midnight–blue (cor = 0.38; *p*–value = 0.35), and tan (cor = 0.37; *p*–value = 0.37). Conversely, the following modules showed GSE41649 had a positive correlation for asthma: yellow (cor = 0.85; *p*–value = 0.01), cyan (cor = 0.84; *p*–value = 0.01), light yellow (cor = 0.66; *p*–value = 0.07), and orange (cor = 0.59; *p*–value = 0.12).

The modules in GSE15283 positively correlated for control were pink (cor = 0.66; *p*–value = 0.02), blue (cor = 0.62; *p*–value = 0.03), and black (cor = 0.47; *p*–value = 0.12), whereas the negatively significant modules were blue (cor = −0.28; *p*–value = 0.32), grey (cor = −0.21; *p*–value = 0.46), and green (cor = −0.08; *p*–value = 0.78). The modules in GSE15283 positively correlated for asthma were turquoise (cor = 0.77; *p*–value = 0.003), grey (cor = 0.48; *p*–value = 0.12), and red (cor = 0.41; *p*–value = 0.19). In contrast, the modules in GSE15283 positively correlated for the post–ICS–treated asthmatic group were brown (cor = 0.59; *p*–value 0.04), yellow (cor = 0.52; *p*–value = 0.08), and green (cor = 0.02; *p*–value = 0.94).

#### 2.6.5. Hub–Gene Detection and Functional Pathway Enrichment

**Figure 8:**
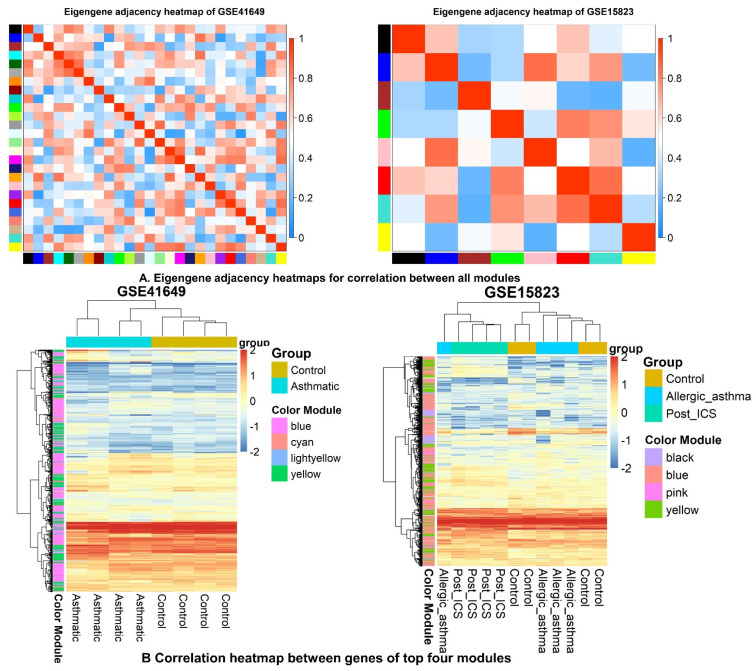
Correlation association between WGCNA modules. (**A**) Eigengene adjacency heatmaps for correlation between WGCNA modules. (**B**) Correlation heatmap between top four modules.

Figure 8 illustrates the correlation between modules in the heatmap of eigengene adjacency. In GSE41649, the modules with positive correlation were cyan, yellow, light yellow, and magenta; in GSE15823, the modules were blue, yellow, pink, and black (Figure 8A). Figure 8B illustrates the relationship between these four modules and gene expression. Mostly, genes have a positive correlation association.

**Figure 9:**
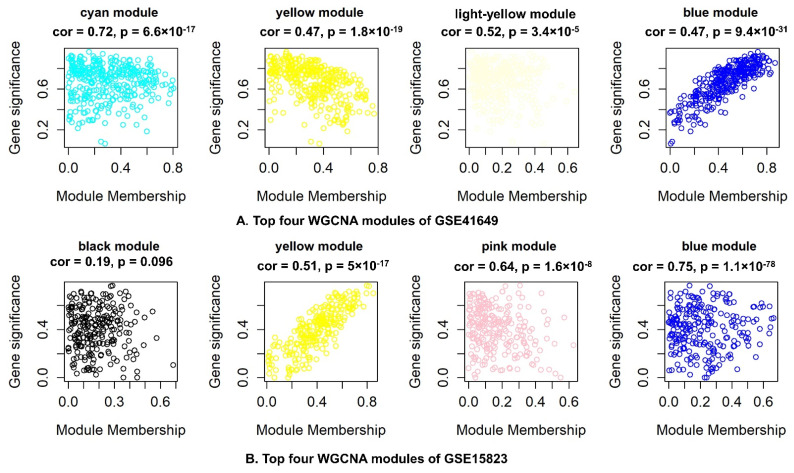
Identification of top four WGCNA modules of datasets with Pearson’s correlation between module membership (MM) and gene significance (G.S.). Top four modules of (**A**) GSE41649 (**B**) GSE15823.

All modules were utilized to identify hub genes highly linked to asthma pathogenesis to analyze the relationship between gene significance (GS) and module membership (MM). We then calculated correlations between MM and GS and analyzed the data (Figure S6, Figure 9). It was determined that the most correlated modules for GSE41649 were cyan, yellow, light yellow, and blue with 98, 329, 57, and 535 hub genes, respectively. GSE15283 had four correlated modules: blue, yellow, pink, and black. These modules contained 429, 236, 63, and 78 hub genes. Functional pathway enrichment was conducted using Cluster Profiler R on both datasets of hub genes. The results of the functional enrichment of both datasets are depicted in Figure S7.

The blue module of GSE41649 was enriched in the following GO biological terms: heart morphogenesis, arachidonic acid, and eicosanoid metabolism, and regulation of voltage–gated calcium channel signaling; for cyan, enriched terms were modulation of phosphatidylinositol 3 kinase signaling, and protein kinase B signaling; for light yellow, enriched terms were the carbohydrate catabolic process, cytosolic transport, and ADP metabolism; for yellow, enriched terms were apoptotic process–related negative modulation of cysteine–type endopeptidase activities and mitochondrial ATP synthesis coupled electron transport. The blue module of GSE41649 enriched for KEGG pathways was the glutathione metabolic pathway, calcium signaling pathway, IL–17 signaling pathway. For cyan, it was arginine biosynthesis. For light–yellow, it was the cholinergic synapse, HIF–1 signaling pathway, and ErbB signaling pathway. For the yellow module, it was vascular smooth muscle contraction, Rap1 signaling, estrogen signaling, and TNF signaling.

In GSE15283, the black and pink modules were enriched in domains concerning cellular compartments (CC) and molecular functions (MF) gene ontologies. The GO BP terms for the blue module of GSE15283 were immune cell activation, positive regulation of cytokine production, interleukin–12 production, and positive regulation of inflammatory response. The KEGG pathways for the blue module of GSE15283 include cytotoxicity induced by natural killer cells, Th1 and Th2 cell differentiation, primary immunodeficiency, the chemokine signaling pathway, and asthma.

#### 2.6.6. GSEA Analysis of Common Genes

We performed GSEA analysis on the common genes of DEG lists and the top modules of both datasets to identify related gene expressions based on the DEG list. In GSE41624, genes were identified as related to GO BP stress response, calcium homeostasis, calcium–mediated signaling, MAPK, phosphatidylinositol 3 kinase, and positive regulation of ERK1 and ERK2. The GSEA KEGG included chemokine signaling, the chemical MAPK signaling pathway, Ras signaling pathway, Rap1 signaling pathway, calcium–mediated signaling pathway, and PI3K–Akt signaling pathway (Figure S8).

In GSE15823, genes were identified as being related to GO BP: positive modulation of TNF production, the ERK1, and ERK2 cascade are regulated, stress response is mediated by G protein–coupled receptor signaling, actin polymerization or depolymerization is regulated, calcium ion transport is regulated, and apoptosis signals are regulated. As a result of KEGG GSEA, we identified interactions of cytokine–cytokine receptors, Rap1 and HIF–1 signaling pathways, cholinergic and serotonergic synapses, and calcium signaling pathways (Figure S9).

#### 2.6.7. Identification of Smooth Muscle Contraction Pathways

**Figure 10:**
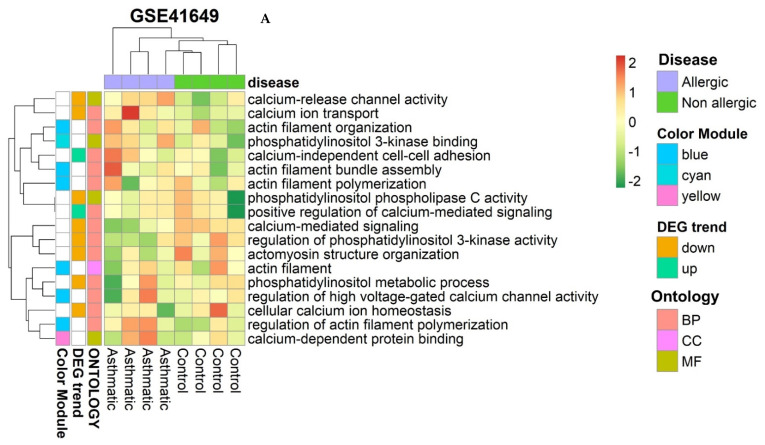

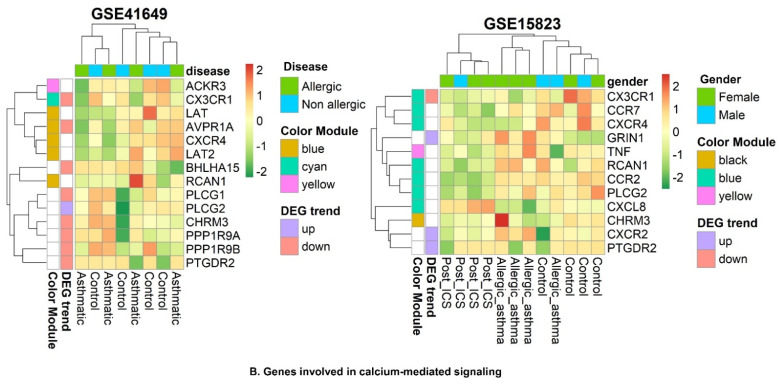
Identifying pathways for calcium regulatory genes involved in smooth muscle contraction. (**A**) GO biological process involved in smooth muscle contraction. (**B**) Regulatory genes involved in the calcium–mediated signaling pathway.

We used cross–functional pathway screening to enhance findings from both datasets related to calcium–mediated signaling and smooth muscle contraction. Using the *hclust* function, genes and functional terms obtained from a particular sample were examined for expression and clustering in various DEG and WGCNA modules. In Figure 10A, GO biological terms are expressed in various groups of GSE41649 and GSE15283. Several key mechanisms are involved in calcium–release channels, calcium ion transport, calcium–mediated signaling, actomyosin structure organization, calcium ion release, import into cell cytosol, and positive modulation of calcium–mediated signaling.

There are many genes in GSE41649 involved in calcium signaling and muscle contraction, including *CX3CR1*, *CHRM3*, *AVPR1A*, *CXCR4*, *RCAN1*, *ACKR3*, *BHLHA15*, *LAT2*, *LAT*, *PLCG2*, *PLCG1*, *PPP1R9B*, *PPP1R9A*, and *PTGDR2*. Several genes were involved in smooth muscle contraction and calcium–mediated signaling for GSE15283, including *PTGDR2*, *PLCG2*, *CX3CR1*, *TNF*, *RCAN1*, *CXCL8*, *CHRM3*, *CCR7*, *CCR2*, *CXCR2*, *CXCR4*, and *GRIN1* (Figure 10B).

#### 2.6.8. Identification of Potentially Active Genes for Bioactive Compounds and Disease

**Figure 11:**
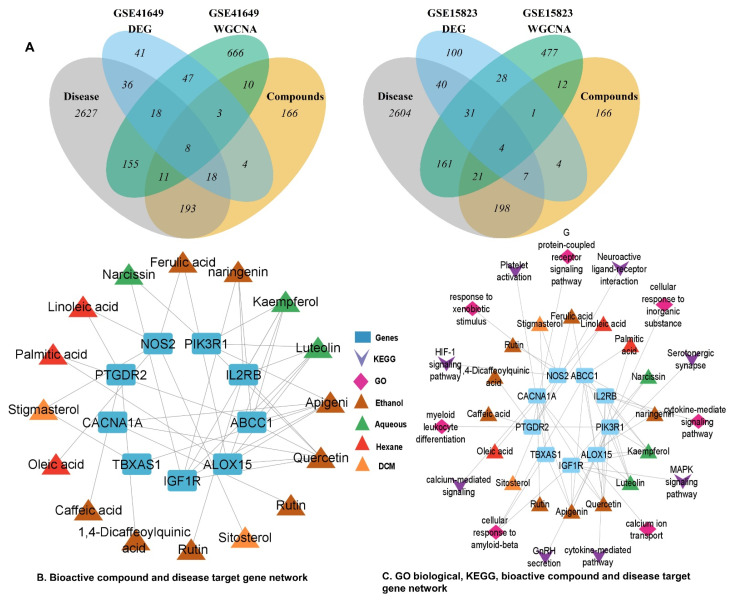
Identification of key genes and network construction. (**A**) Venn diagram of overlapping genes in disease–associated genes, compounds target genes, differentially expressed genes, and WGCNA top 4 modules genes of GSE41649 and GSE15283. (**B**) Compound and disease target genes network (**C**) GO biological process and KEGG pathway network of bioactive compounds and target genes.

A threshold score of more than 50 or a probability greater than 0.5 was used to extract the most likely target profiles for every bioactive molecule from the TCMSP, Swiss–target prediction, and Drugbank databases. A total of 417 genes were identified for *C. melo* seed kernel sequential extracts after removing duplicates. Respiratory–tract–disease genes with the key terms “asthma”, “bronchitis”, and “coughing” were retrieved from PubMed, DisGeNET, GeneCards, and OMIM databases. The target genes were rectified using Uniprot and then validated with the VarElect tool. These targets were used to identify key genes (Figure 11).

#### 2.6.9. Identification of Key Genes

The DEG list, bioactive compounds target genes, disease–associated genes, and the genes of the top four modules were intersected for comparison, and the common or overlapping genes were assumed as key genes for network construction (Figure 11A). Results showed that GSE41649 has eight overlapping genes, including *CACNA1A*, *PIK3R1*, *NOS2*, *PTGDR2*, *ALOX15*, *IGF1R*, *ABCC1*, and *IL2RB*, whereas GSE15283 has four overlapping genes: *IL2RB*, *CACNA1A*, *NOS2*, and *TBXAS1.* The common genes in GSE41649 and GSE1482 were *CACNA1A*, *IL2RB*, and *NOS2* and were considered as the most common key genes for both datasets. Genes from both datasets were pooled and considered for network pharmacology investigations.

### 2.7. PPI and CTP Network Construction with Key Genes

Protein–protein interactions (PPI) of all retained key genes were investigated using Cytoscape’s STRING plugin. The key genes network contained nine nodes and four edges. Network analysis showed that *PIK3R1* (degree = 3) has the highest degree, whereas all other genes have *CACNA1A*, *NOS2*, *ABCC1*, *IGF1R*, *PTGDR2*, *TBXAS1*, *IL2RB*, and *ALOX15*. The C–T–D network had 25 nodes and 41 edges. Quercetin was found with a degree = 12 when networked with target genes, apigenin (degree = 8), kaempferol (degree = 7), luteolin (degree = 5), naringenin (degree = 3), narcissin (degree = 2), and stigmasterol (degree = 2) (Table S4, Figure 11B). The compound target pathway (C–T–P) network was built (Figure 11C). In the C–T–P network, 40 nodes, and 58 edges were found. The degree score of GO biological process terms was calcium ion transport (degree = 8), cytokine–mediated signaling pathway (degree = 6), cellular response to amyloid–beta (degree = 3), cellular response to an inorganic substance (degree = 1), G protein–coupled receptor signaling (degree = 3), and myeloid leukocyte differentiation (degree = 1). Similarly, for KEGG pathways, the degree score was Platelet activation (degree = 6), calcium–mediated signaling (degree = 9), cytokine–mediated pathway (degree = 7), MAPK signaling (degree = 6), Serotonergic synapse (degree = 3), neuroactive ligand–receptor interactions (degree = 3), HIF–1 signaling (degree = 1), and GnRH secretion (degree = 5).

### 2.8. Protein Homology and Validation

Uniprot Model of Interleukin–2 receptor Subunit β (IL2RB): P14784, nitric oxide synthase 2 (NOS2): P35228, and voltage–dependent L–type calcium channel subunit alpha–1C (VGCAC1C): Q13936 were used to generate the sequence.

#### 2.8.1. Physicochemical Characteristics

The physicochemical properties of IL2RB, NOS2, and VGCAC1C were determined using ProtParam. These physicochemical properties of each protein are outlined in Table 3.

#### 2.8.2. Validation of Homology Modeling

**Figure 12:**
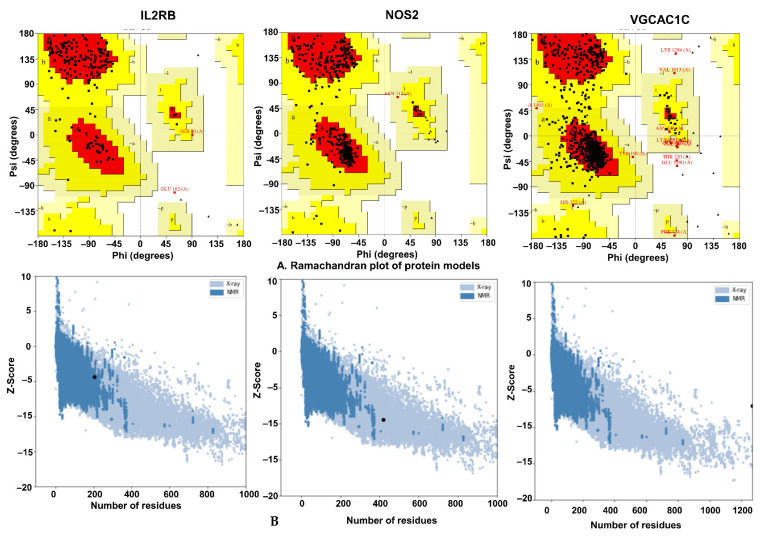
Validation of IL2RB, NOS2, and VGCAC1C models. (**A**) Ramachandran plot of proteins to identify the distribution of amino acid residues. (**B**) Z–score plot of protein models.

A BLAST search was conducted to identify the template of IL2RB, NOS2, and VGCAC1C for homology models (Figure 12). IL2RB had 61 percent identity, 39 percent gaps, and 1781 overall scores on PDB template 5M5E; NOS2 had 56 percent identity, 44 percent gaps, and 8238 overall scores on PDB template 1NSI; calcium ion channels had 71 percent identity and 29 percent gaps on PDB template 7UHF. Ramachandran plots were used to verify the accuracy of the protein model with amino acid distributions in phi and psi angles (Figure 12A). As a result of Ramachandran plot analysis, the amino acid residues in IL2RB, NOS2, and VGCAC1C were distributed in favorable areas in 85.9%, 89.6%, and 88.8%, respectively. In comparison, 0.5%, 0.0, and 0.3% were found in forbidden or disallowed areas. To assess the model quality, we calculated the z–score of the protein structures based on the energy of separation between native and misfolded proteins. Based on the results, IL2RB, NOS2, and VGCAC1C protein models were found to have z–scores of 4–3.33, −9.47, and 7 (Figure 12B). A model of high sequence similarity may be helpful for molecular docking studies if alignment is adequately performed.

### 2.9. Molecular Docking

Molecular docking calculations are helpful for estimating the position of a ligand within the binding site of a target protein. Using physical energy factors (such as solvation energy), docking models can be more accurate [23,25,26]. A docking study was conducted using the models IL2RB, NOS2, and VGCAC1C to evaluate the efficacy of bioactive compounds on target genes. Luteolin binds most strongly to NOS2 (∆G = −47.68 kcal/mol) and IL2RB (∆G = −38.97 kcal/mol), those that have the most negative Gibbs free energy (ΔG) values in comparison to other compounds. Quercetin strongly binds to VGCAC1C (∆G = −40.37 kcal/mol) (Table 4, Table S5, Figure S10).

Based on the results, some ligands exhibit a strong binding affinity for the receptor, as indicated by their highest negative Gibbs free energy (∆G). In order to achieve equilibrium at constant pressure and temperature, a system must have a negative ∆G change and have reached equilibrium. ∆G is believed to be responsible for the stability of a protein–ligand combination depending upon the strength of the interactions between the proteins and the ligands. Alternately, it may be defined as the affinity of the ligand for its acceptor [27]. The inhibitory constant (Ki) is the concentration of inhibitor required to cut the reaction’s maximal rate in half [28]. Therefore, it helps determine whether an inhibitor is effective. In general, Ki determines how much substance is required to block a receptor’s activity. Luteolin had predicted logKi values of −17.48 and −13.70 µM, respectively, for NOS2 and IL2RB. Quercetin had predicted logKi values of −14.30 µM for VGCAC1C.

The Coulomb binding energy, covalent binding energy, lipophilic binding energy, generalized born electrostatic solvation energy, and others are shown in Table S5.

## 3. Discussion

Since ancient times, herbal treatment as supplemental medicine or dietary regimens for treating various complex illnesses has been widely acknowledged as a safe and effective therapeutic. *Cucumis melo* L. seed kernels are an effective traditional healer and can treat various illnesses [29,30,31,32]. The objective of the current study was to look into the possible therapeutic uses of *C. melo* seed kernels for treating chronic disorders. This study investigated the motility of intestinal, tracheal, and urinary longitudinal smooth muscle strips in vitro and in vivo to determine how *C. melo* seed kernel sequential extracts regulate smooth muscle contractions [33]. We found that extracts of *C. melo* seed kernels modulate intracellular calcium homeostasis in smooth muscle contractions, thereby having anti–asthmatic effects.

Asthma is associated with increased smooth muscle mass due to hyperplasia and proliferation of smooth muscle, alterations in the extracellular matrix (ECM), and decreased apoptosis in ASM cells [34]. Th2 cells secreting IL–4, IL–5, and IL–13 play a key role in the inflammatory process in allergic asthma, smooth muscle spasms, and mucus production [35]. During pulmonary inflammation, IκB kinase phosphorylated NF–κB, producing iNOS and COX–2, which are inflammatory mediators [35]. As a result, NF–κB was partly responsible for the remodeling and inflammation of the ASM. In addition to signaling muscle contraction and metabolism, intracellular calcium contributes to cell motility, cell proliferation, protein release, and cell differentiation [34]. The development of asthmatic ASM phenotype and hyperresponsiveness has long been hypothesized to be associated with abnormalities in calcium homeostasis in ASM. In studies, rutin and quercetin significantly reduce eosinophilia and inflammation caused by cigarette smoke, while ovalbumin causes allergic asthma. Both rutin and quercetin reduced IgE levels, Th17 levels, iNOS levels, and cytokines, while stimulating IFN–γ levels [36,37].

These target genes directly or indirectly have a pivotal role in smooth muscle contraction with calcium–mediated signaling and smooth muscle contraction. In molecular docking, rutin and quercetin had a binding affinity with the L–type voltage–gated calcium ion channel (genes: CACNA1C, CACNA1D) and phosphoinositide phospholipase C (gene: PLCB, PLCE), myosin light chain kinase (genes: MYLK, MYL9), and calcium/calmodulin kinase (CAMK2B). Hence, it can be assumed that the strong antispasmodic activity of *C. melo* seed kernels was due to the strong binding affinity of quercetin and rutin towards target proteins, i.e., L–type voltage–gated calcium ion channel and phosphoinositide phospholipase C, myosin light chain kinase, and calcium/calmodulin kinase. It blockaded the signal transduction responsible for contraction. These proposed GSEA, GO, and KEGG pathways were verified with in vitro and in vivo experimentation. Both experiments were performed on the jejunum, trachea, and urinary bladder for the calcium signal transduction pathway on K^+^ (80 mM) and calcium concentration–response curves.

We investigated the impact of calcium ions to determine how *C. melo* seed kernels extract alleviated muscle tension. A well–known physiological process, “excitation–contraction”, is essential for maintaining intracellular calcium homeostasis, which is essential for relaxed and contracted smooth muscles [38]. Calcium entering the cytosol from extracellular fluid or calcium released from the endoplasmic reticulum causes the intestinal smooth muscles to contract spontaneously when calcium levels rise. Voltage–dependent calcium ion channels are vital in controlling intestinal motility [39]. *C. melo* seed kernel sequential extracts were tested for antispasmodic activity at isolated jejunum tissue contractions in a tissue organ bath. Results revealed that *C. melo* seed kernel sequential extracts ease smooth muscle spasms by reducing contractions and inhibiting calcium ions influx into the cell [40], except Cm–ethanol, which, in atropinzed jejunal preparation, relaxed the spontaneous tissue. The smooth muscle relaxant potential of *C. melo* seed kernel sequential extracts was further examined against Potassium chloride (80 and 25 mM) spastic contractions to ascertain whether it interfered with calcium ion channel inhibition or potassium ion channel activation. The potassium chloride (80 mM) triggered an intense depolarization of the membrane action potential of the cells, associated with increases in calcium inward current that produced a prolonged contractile response [41]. *C. melo* seed kernel sequential extracts repolarized this intense depolarization of membrane action potential to relax intestinal smooth muscle and obstruct the influx of calcium current. It was predicted that the following chronicle events would be interrupted [38,42]: (1) the concentration of cytosolic calcium ions was reduced due to a reduction in calcium inward current; (2) As there was insufficient interaction with regulatory protein phosphokinase C, ae calcium–calmodulin complex was not formed; (3) in the absence of calcium–calmodulin, myosin light chain kinase (MLCK) failed to activate, which resulted in a reduction in myosin light chain (MLC) phosphorylation, and; (4) this reduction decreased the availability of phosphorylated MLCs, resulting in the loss of myosin and actin filament crosslinking and the absence of a contractile response [43]. The pretreatment of jejunum tissue with *C. melo* seed kernel sequential extracts suppressed calcium DRCs at 1 and 3 mg/mL and induced a rightward shift comparable to verapamil [44], indicating that *C. melo* seed kernel sequential extracts behaved as calcium ion channel blockers and repolarized membrane action potential [41]. Amira et al. (2008) reported that six flavonoids (apigenin, genistein, quercetin, rutin, naringenin, and catechin) had an antispasmodic effect (at dose 30 µM) by regulating the gastric tone of the stomach. Thus, the antispasmodic and antidiarrheal activity of *C. melo* seed kernel sequential extracts may be attributed to the abundant presence of quercetin, epicatechin, rutin, and apigenin.

We investigated the antispasmodic properties of *C. melo* by inducing action potential depolarization in tracheal and urinary bladder tissue preparations in response to carbachol (1 µM) and potassium chloride (80 mM)–induced contractions [45]. *C. melo* seed kernel sequential extracts demonstrated dose–dependent relaxation of carbachol (1 µM) and potassium chloride (80 mM) elicited contraction, sustaining a repolarization state. Similar results for induced contractions were seen in *C. melo* seed kernel extract on the jejunal tissue preparation. The muscarinic M3 receptor activity of *C. melo* seed kernel sequential extracts on tracheal and urinary bladder preparations was established by the EC_50_ value of carbachol (1 µM). It is known that muscarinic agonists (Ach, carbachol) cause muscular contractions by triggering the inflow of calcium ions into cells or intracellular cellular calcium release. Thus, we assumed that *C. melo* seed kernel sequential extracts reduced carbachol (1 µM)–induced contraction may be mediated through the calcium signaling pathway. The stimulation of the M_3_ muscarinic receptor produced secondary regulatory proteins trisphosphate (IP3) and diacylglycerol (DAG), which are involved in the intracellular calcium–calmodulin complex. Therefore, muscarinic receptors share the same calcium–mediated signaling pathway for smooth muscle contractile response [41,46]—the pretreatment of *C. melo* seed kernel sequential extracts on tracheal preparations caused a rightward shift in carbachol DRCs, such as the verapamil validated hypothesis [47]. In addition, pretreatment with *C. melo* seed kernel sequential extracts on the urine bladder resulted in a rightward shift and calcium DRCs inhibition, which may indicate the function of calcium in the therapeutic action of sequential extracts of *C. melo* seed kernels. Therefore, *C. melo* seed kernel sequential extracts exhibited calcium–mediated signaling and smooth muscle relaxation.

Chan et al. [48] reported the anti–asthmatic activity of quercetin and rutin. This antimuscarinic response was produced due to the blockade of phosphoinositide phospholipase C (PLC), an active member in calcium mediates signaling. As previously shown in molecular docking, PLC showed a strong binding affinity with rutin (−37.75 kcal/mol) and quercetin (26.15 kcal/mol). Phosphoinositide phospholipase C, a key enzyme present under cell members, promotes signal transduction of M_3_ muscarinic receptor to induce contraction in smooth muscles. The activation of the M_3_ muscarinic receptor stimulates PLC, which hydrolyzes phosphatidylinositol 4,5–bisphosphate into two secondary messengers: inositol 1,4,5–trisphosphate (IP_3_) and diacylglycerol (DAG). IP_3_ stimulates inositol 1,4,5–trisphosphate receptors (IP_3_R) on the sarcoplasmic reticulum to release calcium ions, increasing cytosolic calcium levels. DAG, along with calcium, activates a regulatory protein kinase C (PKC, gene: PRKCA), through which phosphorylation of calmodulin occurs to form a calcium/calmodulin complex. This calcium/calmodulin complex activates another myosin light chain kinase (MLCK) that causes phosphorylation of myosin light chains (MLCs), phosphorylated MLCs, and actin, which form an interaction network to produce a contractile response [38,39,41]. Hence, *C. melo* seed kernel sequential extracts exert their bronchodilator and dysuria action by decreasing cytosolic calcium release from the sarcoplasmic reticulum and blockading the signal transduction of the muscarinic receptor pathway of contractile response.

Diarrhea is the abnormal expulsion of low–consistency stool due to disturbance in electrolytes and water transport in the intestine. Castor oil induces electrolyte and water transport changes in the intestine to cause diarrhea and increase peristaltic movements [49]. Our present study studied antidiarrheal, antiperistalsis, and fluid intestinal accumulation activities of *C. melo* seed kernel sequential extracts. The antispasmodic properties of *C. melo* seed kernel sequential extracts also inhibit the movement of charcoal meal at 150 and 300 mg/kg, producing a substantial decrease in peristalsis, diarrhea, and electrolyte imbalance. Castor oil contains ricinoleic acid [50,51], an active component that may enhance intestinal fluid and electrolytes [52] when the mucosa of the intestinal tract breaks it down. Subsequently, significant contractions occurred in the transverse colon and distal colon. *C. melo* seed kernel sequential extracts inhibited fluid secretion and castor oil–induced diarrhea [53] in animals. *C. melo* seed kernel sequential extracts exert antiperistaltic, antisecretory, and antidiarrheal effects mediated through calcium channel inhibition mechanisms, similar to loperamide and verapamil [54,55].

## 4. Materials and Methods

### 4.1. Preparation of Extract

In the summer of 2018, a botanist from Bahauddin Zakariya University, Multan (60000), Pakistan, verified and validated *Cucumis melo* L seed kernels and fruit obtained from locally framed fruits. A voucher specimen (Sp. Pl. 1011–1753) was placed at the herbarium of the Institute of Biology and Applied Sciences, Bahauddin Zakariya University, Multan (60000), Pakistan. The inner portion of the seed kernels was manually separated from the husk and processed into a coarse powder using an herbal grinder. Then, it was processed in a Soxhlet system using *n*–hexane, dichloromethane (DCM), ethanol, and water at their boiling points for 24 ± 2 h. Solvents were evaporated under reduced pressure at 35 ± 2 °C through rotary evaporation to produce yellowish–brown greasy extracts. The yields of *Cucumis melo* seed kernel sequential extracts, i.e., Cm–hexane, Cm–DCM, Cm–ethanol, and Cm–aqueous, were 41.60, 9.31, 16.96, and 21.20 g, respectively, with an overall yield of 64%. Dried extracts thus obtained were stored at −20 °C in an amber glass jar and were later screened for their pharmacological properties. On the day of the experiment, distilled water or normal saline was used to dissolve and dilute the extract to the required concentration. Cm–hexane and Cm–DCM were dissolved in Tween–80 and diluted in normal saline or distilled water.

### 4.2. Chemicals

All analytical–grade solvents and chemicals used in this experiment were provided by Sigma Compounds Co., St. Louis, MO, USA.

### 4.3. Phytochemical Analysis of C. melo Seed Kernel Sequential Extracts

LC/ESI–MS/MS studies were conducted for the determination, characterization, and quantification of the constituents present in polarity–based sequential extracts of *C. melo,* following parameters and thresholds mentioned in one of our earlier reports [23,24].

#### 4.3.1. Preparation of Samples

A 1.0 mL amount of 100 percent methanol (MeOH) was mixed with sequential extracts of *C. melo* (100 mg) to prepare samples for chromatographic analysis. We centrifuged the mixture for 14 ± 2 min at 14,000 rpm and then filtered the supernatant with a 0.22 µm syringe filter.

#### 4.3.2. LC/ESI–MS/MS Analysis

*C. melo* seed kernel sequential extracts were investigated using LC/ESI–MS/MS to determine their phytoconstituents [56]. The sample was injected directly into an ESI probe for negative and positive ion modes. The mobile phases for Cm–ethanol and Cm–aqueous were made of Solvent–A, which contains 0.1% formic acid (FA) in MeOH, and Solvent–B, which contains 0.1% F.A. in acetonitrile (ACN) and water (20:80), respectively. The mobile phases for Cm–hexane and Cm–DCM were made of Solvent–A, which contains 0.1% FA in MeOH, and Solvent–B contains 0.1% FA in ACN, respectively. Other optimal chromatographic and mass spectroscopic parameters are detailed in earlier reports [23,24].

#### 4.3.3. RP–HPLC Quantification and Method Validation

We performed HPLC analysis to confirm and quantify the phytoconstituents of *C. melo* seed kernel sequential extracts [24,57]. The mobile phases for Cm–ethanol and Cm–aqueous were made of Solvent–A, which contains 0.1% trifluoroacetic acid (TFA) in MeOH, and Solvent–B contains 0.1%canA with ACN and water (20:80), respectively. On the other hand, the mobile phases for Cm–DCM were made of Solvent–A, which contains 0.1% TFA in MeOH, and Solvent–B contains 0.1% TFA and ACN, respectively. Other optimal chromatographic and analytical method validation processes are detailed in our previous publications [23,24].

### 4.4. Ethical Committee Provision

The animal ethical committee of the Department of Pharmacology permitted the experimental methods for animal studies to vide E.C./04PhDL/S2018. The research was conducted following the Commission on Laboratory Animal Resources [58]. We used male and female albino rabbits (1.26–1.65 kg), Sprague–Dawley rats (155–180 g), and albino mice (17–26 g) throughout this study. Animals were housed in controlled conditions with a standard prescribed diet. For in vitro and in vivo testing, rats and mice were dislocated at the cervical spine, and rabbits were sacrificed with a sharp knife.

### 4.5. Isolated Tissue Experimentation for Smooth Muscle Contraction

For in vitro investigations, we followed the protocols of Saqib and Janbaz [41] and Wahid et al. [57]. We recorded the physiological response of the tissue with an isotonic and isometric transducer.

#### 4.5.1. Isolated Rabbit Jejunum Preparations

Jejunum portions were dissected and placed in a tissue organ containing Tyrode’s solution continuously recirculated with the carbogen gas at 37 ± 0.5 °C to keep them alive and ready for use under preload tension of 1 g. Before administering the *C. melo* seed kernel sequential extracts, the jejunum tissue was equilibrated for 25 ± 5 min. Test samples (extracts) were applied to the bath solution containing the jejunum tissue. The decrease in spontaneous rhythmic contractions for each tested sample was measured with repeated flushes of Tyrode solution every 10 min [47]. As reported previously, the calcium–mediated signaling pathways are involved in smooth contraction by triggering the calcium ion channel closing or the potassium channel opening [38]. The potassium chloride (80 mM) initiated the closing of the calcium ion channel, and the potassium ion channel was opened with potassium chloride (25 mM) [41,56]. Potassium chloride (80 mM) depolarized the cells and induced a calcium ion influx, which may alter smooth muscle contraction. The extract may be categorized as a calcium channel blocker if it inhibits these contractions [43]—the doses of *C. melo* seed kernel sequential extracts were successively administered to establish an inhibitory effect.

In addition, the calcium–antagonistic properties of *C. melo* seed kernel sequential extracts were examined. The preparation of jejunal tissue was pretreated with potassium chloride (80 mM), subsequently incubated in an EDTA Tyrode solution for 30 ± 7 min, and incubated for 55 ± 7 min in calcium–free Tyrode solution that is rich with potassium, to reduce or diminish intracellular calcium concentrations. Following incubation, doses of calcium were gradually added to produce superimposable control calcium dose–response curves (DRCs). Calcium DRCs were produced in Cm–hexane, Cm–DCM, Cm–ethanol, and Cm–aqueous extract pretreated jejunal preparations [43].

#### 4.5.2. Isolated Rabbit Tracheal Preparations

We dissected the rabbit tracheal tissue, removed the adherent substances, formed the 2 to 3 mm wide rings, and each ring was incised longitudinally to produce sandwich smooth muscle tracheal strip preparation. Each preparation was placed in a Krebs buffer–filled tissue organ bath (pH 7.4) continuously circulated with carbogen at 37 ± 0.5 °C, under tension of 1 g. Before administering *C. melo* seed kernel sequential extracts, the tracheal preparation was stabilized for 55 ± 10 min. The bronchodilator activity of *C. melo* seed kernel sequential extracts was studied with potassium chloride (80 and 25 mM) and CCh (1 µM). A dose–dependent inhibitory response was established by cumulative administration of Cm–hexane, Cm–DCM, Cm–ethanol, and Cm–aqueous extracts. The preparation of tracheal tissue was treated with CCh (1 µM), then incubated for 55 ± 10 min in normal Kreb’s solution. Following incubation, different concentrations of carbachol were gradually administered to the tissue preparation to form superimposable control carbachol dose–response curves (DRCs). The tracheal preparation was incubated with *C. melo* seed kernel sequential extracts for 55 ±10 min, and then carbachol DRCs were produced. To ascertain the presence of muscarinic receptor antagonism action, these carbachol DRCs of *C. melo* seed kernel sequential extracts were compared to control carbachol DRCs [43].

#### 4.5.3. Isolated Urinary Bladder Preparations

We dissected the rabbit urinary bladder tissue, removed the adherent substances, and formed 2 to 3 cm wide strips. Each preparation was placed in a Krebs buffer–filled tissue organ bath continuously gassed with carbogen at 37 ± 0.5 °C, under tension of 1 g. Before administering the test drug, the tissue preparation was equilibrated for 55 ± 5 min. The dysuria activity of *C. melo* seed kernel sequential extracts was studied with potassium chloride (80 and 25 mM) and CCh (1 µM). A dose–dependent inhibitory response was established by cumulative administration of Cm–hexane, Cm–DCM, Cm–ethanol, and Cm–aqueous extracts. In addition, calcium antagonistic properties of *C. melo* seed kernel sequential extracts were examined. The preparation of bladder tissue was pretreated with potassium chloride (80 mM), subsequently incubated in an EDTA Tyrode solution for 30 ± 7 min, and incubated for 55 ± 7 min in calcium–free Tyrode solution that is rich with potassium to reduce or diminish intracellular calcium concentrations. Following incubation, doses of calcium were gradually added to produce superimposable control calcium dose–response curves (DRCs). Calcium DRCs were produced in Cm–hexane, Cm–DCM, Cm–ethanol, and Cm–aqueous extract pretreated bladder preparations [43].

### 4.6. In Vivo Experimentation

#### 4.6.1. Evaluation of Maximum Tolerated Dose

*C. melo* seed kernel sequential extracts were tested to their maximum tolerated dose. Rats were orally given *C. melo* seed sequential extracts at doses of 50, 100, 150, 200, and 300 mg/kg every day for 28 days following the OECD guidelines, whereas the control rats were given saline. During the 28–day study, death rates, body weights, behavioral changes, and clinical signs of discomfort were recorded [59].

#### 4.6.2. Protocol and Design

We randomly divided the mice, of either sex, into six groups with five animals each. Before the trial, water was freely available to all groups, but the food was withdrawn for 10 ± 3 h.

Group I was orally fed with normal saline (10 mL/kg).
Group II was orally fed with castor oil or charcoal meal.
Group III and IV were orally fed with 10 mg/kg of verapamil and loperamide.
Groups V and VI were orally fed with 150 and 300 mg/kg of Cm–hexane.
Groups VII and VIII were orally fed with 150 and 300 mg/kg of Cm–DCM.
Groups IX and X were orally fed with 150 and 300 mg/kg of Cm–ethanol.
Groups XI and XII were orally fed with 150 and 300 mg/kg of Cm–aqueous.

#### 4.6.3. Charcoal Meal GI Transit Test

In the antiperistalsis activity [41] of *C. melo* seed kernel sequential extracts, all animals received the charcoal meal orally after 15 min except Group I. Animals were sacrificed after 30 min, and their small intestines were excised to estimate the distance traveled by charcoal. The percentage of the peristaltic index was calculated [24].

#### 4.6.4. Castor Oil–Induced Diarrhea

Castor oil–induced diarrheal activity [57] of *C. melo* seed kernel sequential extracts; all animals received the castor oil orally after 30 min except Group I. After 6–7 h, wet fecal spots were observed on white paper. The percentage of protection for defecation was calculated [24].

#### 4.6.5. Castor Oil–Induced Intestinal Fluid Accumulation

In the antisecretory activity [44] of *C. melo* seed kernel sequential extracts, mice in all groups received the castor oil orally after 15 min except for Group I. Animals were sacrificed after 30 min and ligated pylorus and caecum ends of the small intestine before being removed. The weight of the small intestine with and without intestinal fluid was monitored. The amount of fluid accumulated (g) was determined by weighing the fluid accumulated in the intestine [24].

### 4.7. WGCNA and DEG Studies

#### 4.7.1. Data Download and Preprocessing

Asthma trait datasets GSE41649 and GSE15283 were acquired using the NCBI GEO (https://www.ncbi.nlm.nih.gov/geo/ (accessed on 22 June 2022)). The coefficient of variation was calculated after normalizing both datasets and eliminating the expression of genes with low expression levels.

#### 4.7.2. Differentially Expressed Genes

In this study, differentially expressed genes (DEGs) from GSE41649 and GSE15823 were evaluated using the Limma package (v3.52) of R in the context of the following contrasts: *bronchial asthma* vs. *control* for GSE1484, *asthma* vs. *control*, and *post–ICS* vs. *asthma* for GSE15283 with log2 fold–change thresholds of ≥0.2 and 1, and *p*–value ≥ 0.05. The data were plotted using the R package ggplot2 (v3.3). In the following step, we determined the top 15 DEG genes and separated them into groups based on the *hclust* method.

#### 4.7.3. Weighted Correlation Network Analysis

Identification of modules: We used the R package WGCNA (v1.70) to analyze GSE41649 and GSE15823 datasets for co–expression networks. The expressions of all genes were grouped using hclust to eliminate outliers. A Pearson test was used to create an adjacency matrix, and a paired gene expression similarity matrix was generated. The scale–free gene co–expression topological approach was used to evaluate the adjacency matrix at the lowest possible soft–threshold values. We constructed dissimilarity TOM (dissTOM) and topological overlap matrices (TOM) to assess gene expression profiles. A minimum module size of 30 was calculated to identify the co–expressed modules of genes. The modules with high similarity scores were combined at a threshold of 0.25.

Construction of module–trait relationships: We identified modules with a strong correlation to clinical characteristics following Module Eigengene (ME). Gene expression for each module was associated with its primary component as an eigenvector. An eigengene is the average gene expression pattern representing a module’s central element.

Detection of hub genes: We analyzed gene significance scores (GS) to identify asthma–associated gene expressions. Each gene expression was assigned a module membership (MM) to determine the correlation between ME and gene expression. The most significant genes in the modules are strongly associated with GS and MM, suggesting they are strongly associated with clinical characteristics. In addition, they can be used to identify module hub genes as well as create network diagrams.

#### 4.7.4. Functional Annotation and Pathway Enrichment

The Cluster Profiler (v4.4.4) software was used to analyze functional gene ontology annotation and pathway enrichment from WGCNA modules and DEG genes of each dataset with an FDR ≤ 0.05 threshold.

#### 4.7.5. Gene Set Enrichment Analysis (GSEA)

In both datasets, GSEA was performed to determine the biological significance of significantly overlapping gene expressions between the WGCNA and DEG genes. Gene expression levels were divided into low and high categories according to their expression levels. GSEA analysis of KEGG and GO biological processes was conducted with Cluster profiler software with an FDR ≤ 0.05 threshold.

#### 4.7.6. Screening of Disease and Bioactive Compounds Associated with Target Genes

The potential genes associated with the bioactive substances of *C. melo* seed kernel sequential extracts were acquired from the Drugbank (http://drugbank.com, accessed on 24 August 2021) and the Swiss–target prediction tool (http://www/swisstargetprediction.ch, accessed on 24 August 2021). The key terms “asthma”, “bronchitis”, and “coughing” were used to obtain the disease–associated genes on GeneCards (https://www.genecards.org, accessed on 24 August 2021), DisGeNET (http://www.disgenet.org/web/DisGeNET, accessed on 24 August 2021), PubMed (https://pubmed.ncbi.nlm.nih.gov, accessed on 24 August 2021), and Online Mendelian Inheritance in Man (OMIM) (https://www.omim.org, accessed on 24 August 2021).

#### 4.7.7. Detection of Key Genes

We identified key genes based on the intersection of genes of bioactive substances, disease, WGCNA, and DEGs using the VennDiagram (v1.7.3) R package. The relationship between bioactive chemicals, illness, WGCNA, and DEGs was represented through a Venn diagram.

### 4.8. Construction of PPI, Bioactive Compounds, and Functional Enrichment Networks

Cytoscape 3.8.0 was used to construct and evaluate the bioactive compounds and disease–associated target genes (C–T–D), protein–protein interaction (PPI), bioactive compounds, disease–associated target proteins, and pathway (C–T–P) networks. PPI was constructed string plugin of Cytoscape.

### 4.9. Protein Homology Modeling

We used the Uniprot database to obtain high–quality protein sequences (https://www.uniprot.org/, accessed on 27 August 2021). Uniprot protein sequences were blasted against one or more PDB templates to identify a three–dimensional homology model (Maestro v11.8, Schrodinger suite 2018–4). In brief, we initially evaluated the Uniprot sequence in the Extasy ProtParam tool (http://www.web.expasy.org/protparam, accessed on 30 August 2021) for physical and chemical characteristics [23,60]. Homology modeling involves the following steps: (1) import of Uniprot sequence, (2) BLAST the sequence in the PDB Database, (3) sequence alignment, and (4) model building. After modeling the protein, the model was tested and submitted for a protein reliability report. The Prime Refine–Loops and Prime Minimize modules were applied to refine the constructed model if protein reliability did not meet expectations. A Ramachandran plot using PROCHECK and SAVES v6 (https://saves.mbi.ucla.edu/, accessed on 30 August 2021) was used to validate the minimized protein model [61,62].

### 4.10. Molecular Docking

The various modules of Maestro and BIOVIA Discovery Studio 2021 were used to carry out molecular docking according to the previously mentioned parameters and thresholds [23]. Two–dimensional ligands from NCBI PubChem (https://pubchem.ncbi.nlm.nih.gov, accessed on 28 August 2021) were optimized, minimized, and ionized using the LigPrep module. The protein preparation module was then employed for assigning hydrogen bonds, minimization of het–states, reducing zero–order, disulfide bond formation, ionization, and optimization of 3D structures of the protein model. Furthermore, structural gaps were filled with the help of the prime tool, and het groups were protonated at a pH of (7.0 ± 2.0) using the Epik tool. At a pH of 7.0, PROPKA was employed for the optimal arrangement of hydrogen bonds in protein models, and OPLS3e was used for constraint energy minimization. To facilitate ligand interaction, the sitemap module was utilized to identify the coordinates for the protein binding pockets and the cubic grid box of the receptor grid generation module. The molecular docking was performed with Glide’s extra precision using prepared ligands, proteins, and receptor grid files. The Epik tool was utilized to apply penalties to the docking score. The Prime MM–GBSA module calculated the binding energies of Glide ligand–protein complexes. As reported in earlier studies, the predicated inhibition constant (Ki) was calculated [61,62].

### 4.11. Software

Physiological responses of tissues were recorded using LabChart Pro 7. Molecular docking was carried out using Maestro v11.8 (Schrodinger suite 2018–4) and BIOVIA Discovery Studio 2021. The statistical analysis was conducted by GraphPad Prism 8 and RStudio (version 2021.02.3), and the network was constructed using Cytoscape 3.8.0.

## 5. Conclusions

In summary, LC/ESI–MS/MS and HPLC analyses showed the presence of stigmasterol, rutin, kaempferol, quercetin, apigenin, and luteolin in *C. melo* seed kernel sequential extracts. In addition, an integrated approach of DEGs and WGCNA of asthmatic patients was employed to find the probable regulatory gene network involved in calcium–mediated smooth muscles of the extracts. This investigation suggests that *C. melo* seed kernel sequential extracts exert antispasmodic, antiperistaltic, antidiarrheal, dysuric, and anti–asthmatic activities by modulating contractile response via a calcium–mediated signaling pathway to repolarize the membrane action potential of a smooth muscle cell. However, the presence of stigmasterol, rutin, kaempferol, quercetin, apigenin, and luteolin in *C. melo* seed kernel sequential extracts may be responsible for the bioactivities of the *C. melo* sequential extracts. However, more research is needed to understand and corroborate the putative modes of action and pathways responsible for the medicinal benefits of *C. melo* seed kernels.

## Figures and Tables

**Table 1:** HPLC quantification and technique validation of *C. melo* seed kernel sequential extracts for phytochemicals.

Fraction	Analytes	λ (nm)	Rt (min)	Linear Regression Data			LOD µg/mL	LOQ µg/mL	Concentration µg/g	Precision * (RSD %)		Recovery *		Analytes + Extracts (µg/g)	
				Range µg/mL	Equation	r^2^				Inter–Day	Intra–Day	Mean	RSD %	50 µg	100 µg
DCM	Umbelliferone	270	5.3	7.81–500	y =147.14x + 22.15	0.9996	0.69	2.08	319.97	0.94	1.14	98.65	1.30	369.45	419.09
	Stigmasterol		18.2	7.81–500	y =163.15x + 28.64	0.9998	0.61	1.84	324.69	1.88	1.03	98.26	0.86	374.17	423.80
	β–sitosterol		19.5	7.81–500	y = 105.11x + 9.81	0.9993	0.59	1.79	216.78	0.63	0.71	99.34	1.71	266.41	316.11
Ethanol	Caffeic acid	280	8.3	7.81–500	y =209.19x + 21.41	0.9999	0.44	1.32	341.27	1.07	0.30	99.02	0.76	390.72	440.34
	Rutin		9.7	7.81–500	y =263.12x + 20.66	0.9999	0.47	1.44	752.61	1.25	1.49	99.31	0.67	801.49	850.82
	Quercetin		13.8	7.81–500	y =181.13x + 32.08	0.9994	0.66	2.02	768.89	1.69	1.28	99.60	0.99	817.74	867.07
	Apigenin		17.7	7.81–500	y =228.17x + 18.10	0.9999	0.27	0.87	572.91	1.29	1.62	98.85	0.94	622.04	671.50
	Ferulic acid		22.9	7.81–500	y =220.98x + 28.21	0.9999	0.35	1.05	417.12	1.63	1.33	99.38	1.14	466.47	516.03
	1,4–Dicaffeoylquinic acid	320	7.2	7.81–500	y =198.31x + 28.16	0.9997	0.39	1.18	610.34	0.90	1.75	99.25	0.71	659.42	708.85
Aqueous	Kaempferol	280	19.4	7.81–500	y =158.19x + 21.65	0.9999	0.34	1.03	791.37	0.92	1.83	98.72	1.27	840.19	889.50
	Luteolin		12.4	7.81–500	y =174.19x + 21.61	0.9999	0.41	1.26	624.71	1.92	1.56	99.16	0.78	673.77	723.19
	Hesperidin	320	17.2	7.81–500	y =234.21x + 15.73	0.9997	0.22	0.66	645.14	0.93	1.17	99.15	0.95	694.17	743.58
	Narcissin		12.5	7.81–500	y =220.16x + 28.19	0.9999	0.38	1.16	596.78	1.12	2.25	98.97	0.99	645.87	695.32

* All values are the mean ± S.D. taken as triplicates. All values are mean ± S.D. triplicate analyses for three days. The percent coefficient of variation (RSD %): (S.D./Mean) × 100.

**Table 2:** The half–maximal effective concentration (EC_50_) of *C. melo* seed kernel sequential extracts on isolated tissues preparations.

	Cm–Hexane (mg/mL)	Cm–DCM (mg/mL)	Cm–Ethanol (mg/mL)	Cm–Aqueous (mg/mL)	Verapamil (µM)
Jejunum					
Spontaneous	0.1767 (0.1244–0.2636; 95% CI)	0.2490 (0.1873–0.3344; 95% CI)	Spasmogenic	4.662 (2.125–16.28; 95% CI)	0.04725 (0.03764–0.05968; 95% CI)
K^+^ (80 mM)	0.2437 (0.1636–0.3969; 95% CI)	0.3128 (0.2484–0.3982; 95% CI)	2.089 (1.218–4.141; 95% CI)	0.5439 (0.4209–0.7075; 95% CI)	0.1194 (0.09889–0.1445; 95% CI)
K^+^ (25 mM)	0.1112 (0.08209–0.1532; 95% CI)	0.1926 (0.1281–0.2919; 95% CI)	1.076 (0.8378–1.402; 95% CI)	0.2654 (0.1822–0.3938; 95% CI)	0.01595 (0.01303–0.01956; 95% CI).
Trachea					
K^+^ (80 mM)	0.9567 (0.6366–1.482; 95% CI)	2.596 (1.855–3.796; 95% CI)	4.055 (2.838–5.896; 95% CI)	1.448 (1.186–1.783; 95% CI)	0.2564 (0.2111–0.3118; 95% CI)
CCh (1 µM)	0.5497 (0.4305–0.7191; 95% CI)	0.8250 (0.5890–1.176; 95% CI)	3.393 (2.357–5.184; 95% CI)	0.1972 (0.1556–0.2510; 95% CI)	0.06765 (0.05113–0.09052; 95% CI)
K^+^ (25 mM)	0.05417 (0.03680–0.07970; 95% CI)	0.7764 (0.5116–1.312; 95% CI)	1.726 (1.166–2.714; 95% CI)	0.2748 (0.2116–0.3606; 95% CI)	0.01278 (0.01017–0.01608; 95% CI).
Urinary Bladder					
K^+^ (80 mM)	0.5107 (0.3928–0.6686; 95% CI)	1.124 (0.8837–1.451; 95% CI)	1.607 (1.293–2.022; 95% CI)	0.2199 (0.1667–0.2924; 95% CI)	0.1036 (0.08172–0.1330; 95% CI)
CCh (1 µM)	0.2540 (0.1859–0.3521; 95% CI)	0.4637 (0.3606–0.6092; 95% CI)	0.6436 (0.5133–0.8126; 95% CI)	0.7115 (0.04493–0.1140; 95% CI)	0.1371 (0.1092–0.1723; 95% CI)
K^+^ (25 mM)	0.4152 (0.3313–0.5281)	0.7650 (0.5168–1.161; 95% CI)	1.461 (1.185–1.821; 95% CI)	0.1602 (0.1195–0.2154; 95% CI)	0.00944 (0.007502–0.01187; 95% CI)

**Table 3:** Physiochemical properties of proteins.

Parameters	IL2RB	NOS2	VGCAC1C
Molecular weight	61,117.20	131,117.23	248,976.62
Number of amino acid residues	551	1153	2221
Maximum amino acid residues	12.5% Leu, 10.5% Pro, 8.9% Ser	10.1% Leu, 6.8% Ser, 6.6% Glu	10.2% Leu, 7.7% Ala, 7.0% Ile
Negatively charged residues (Asp + Glu)	62	128	240
Positively charged residues (Arg + Lys)	40	134	255
Theoretical isoelectric point (pI)	4.93, indicating its acidic nature	8.20, indicating its basic nature	6.13, indicating its acidic nature
Instability index *	58.62	48.97	48.87
Aliphatic index	81.78	79.56	92.53
GRAVY score	−0.273	−0.385	−0.052
Extinction coefficient (M^−1^cm^−1^) **	109,150	172,255	244,660

* Instability index: a value ≥40 is considered unstable; ** can extinction coefficient for Cys, Trp, and Tyr concentrations at 280 nM.

**Table 4:** The binding energies (kcal/mol) of docked ligand–protein complex calculated with Prime MM–GBSA.

Compounds	Docking Score (kcal/mol)	Glide Energy kcal/mol)	∆G Bind (kcal/mol)	pKi (µM)	∆G Hbond (kcal/mol)	∆G vdW (kcal/mol)	Residue–Ligand Interactions with Distance (Å)	
							Hydrogen Bonds	Electrostatic/Hydrophobic Bonds
Interleukin–2 Receptor Subunit β (IL2RB)								
Rutin	−6.82	−41.93	−36.20	−12.49	−2.39	−25.23	Conventional hydrogen bond: Asn87 (1.78 Å), Asn43 (1.85 Å), Asn43 (1.90 Å), Arg131 (1.60 Å), Carbon hydrogen bond: Ser45 (2.49 Å), Asn87 (2.56 Å), Asn43 (3.03 Å)	Pi–Cation; Pi–Donor hydrogen bond: Arg131 (4.03 Å), Pi–Sulfur: Met133 (4.25 Å), Pi–Alkyl: Pro222 (4.91 Å), Pro222 (4.88 Å), Arg131 (5.47 Å)
Luteolin	−6.22	−35.82	−38.97	−13.70	−2.36	−24.03	Conventional hydrogen bond: Arg131 (1.62 Å), Phe37 (2.33 Å), Carbon hydrogen bond: Met133 (2.88 Å), Ala134 (1.84 Å), Arg131 (2.32 Å)	Pi–Sigma: Pro222 (2.61 Å), Pi–Cation: Arg131 (4.30 Å), Pi–Sulfur: Met133 (4.22 Å), Pi–Alkyl: Met133 (5.06 Å), Pro222 (4.45 Å)
Quercetin	−3.85	−42.28	−28.68	−9.23	−2.25	−29.05	Conventional hydrogen bond: Ser45 (2.91 Å), Asn87 (1.91 Å), Arg131 (2.18 Å), Arg131 (2.54 Å), Phe37 (3.05 Å), Arg131 (3.10 Å), Asn43 (2.41 Å), Carbon hydrogen bond: Ser45 (2.67 Å)	Pi–Sigma: Pro222 (2.72 Å), Pi–Sulfur: Met133 (5.76 Å), Alkyl: Val47 (4.63 Å), Pi–Alkyl: Ala134 (4.67 Å), Pro222 (4.34 Å), Met133 (5.12 Å)
Umbelliferone	−3.52	−26.52	−35.59	−12.23	−1.10	−20.30	Conventional hydrogen bond: Ala134 (2.17 Å), Arg131 (1.64 Å), Carbon hydrogen bond: Pro222 (3.07 Å)	Pi–Cation: Arg131 (4.57 Å), Pi–Sulfur: Met133 (3.73 Å), Met133 (3.77 Å), Pi–Alkyl: Pro222 (4.87 Å)
Kaempferol	−3.40	−31.55	−30.08	−9.83	−1.81	−24.49	Conventional hydrogen bond: Arg131 (2.05 Å), Arg131 (1.71 Å), Trp223 (2.37 Å), Carbon hydrogen bond: Ser45 (2.60 Å)	Pi–Cation: Arg131 (4.46 Å), Pi–Sulfur: Met133 (4.24 Å), Met133 (3.98 Å), Pi–Alkyl: Pro222 (5.35 Å), Ala134 (3.71 Å), Pro222 (4.56 Å)
Ferulic acid	−3.27	−22.50	−20.23	−5.56	−1.12	−21.03	Conventional hydrogen bond: Arg131 (2.25 Å), Arg131 (1.91 Å), Carbon hydrogen bond: Phe37 (2.48 Å), Asn43 (2.74 Å), Asn43 (2.58 Å), Phe37 (2.73 Å)	Pi–Cation: Arg131pi–Sulfur: Met133 (3.88 Å)
Apigenin	−2.97	−30.28	−28.44	−9.12	−1.81	−25.27	Conventional hydrogen bond Arg131 (2.06 Å), Arg131 (1.72 Å), Trp223 (2.39 Å), Carbon hydrogen bond: Ser45 (2.62 Å)	Pi–Cation: Arg131 (4.46 Å), Pi–Sulfur: Met133 (4.24 Å), Met133 (3.99 Å), Pi–Alkyl: Pro222 (5.32 Å), Ala134 (3.71 Å), Pro222 (4.56 Å)
Verapamil	−1.60	−37.75	−46.89	−17.13	−1.10	−36.91	Conventional hydrogen bond: Ser45 (2.76 Å), Arg131 (2.03 Å), Carbon hydrogen bond: Ser45 (2.62 Å), Ser45 (2.69 Å), Asn43 (2.50 Å), Phe37 (2.55 Å), Asn43 (2.68 Å), Phe37 (2.72 Å), Arg131 (2.61 Å)	Pi–Cation; Pi–Donor hydrogen bond: Arg131 (4.13 Å), Pi–Sigma: Val79 (2.94 Å), Pi–Sulfur: Met133 (4.45 Å), Alkyl: Val47 (4.59 Å), Val79 (4.57 Å), Val79 (4.78 Å), Pi–Alkyl: Ala85 (3.89 Å)
Nitric Oxide Synthase 2 (NOS2)								
Rutin	−14.69	−75.74	−18.93	−4.99	−4.77	−56.65	Conventional hydrogen bond: Ile201 (1.92 Å), Glu377 (2.07 Å), Pro350 (2.61 Å), Cys200 (3.03 Å), Arg199 (1.63 Å), Asn370 (1.84 Å), Arg199 (2.99 Å), Carbon hydrogen bond: Gly371 (2.95 Å), Glu377 (2.55 Å), Arg199 (2.51 Å), Arg199 (2.98 Å), Trp463 (2.56 Å)	Pi–Anion: Cys200 (3.87 Å), Pi–Pi Stacked: Trp194 (4.22 Å), Trp194 (3.65 Å), Trp194 (5.32 Å), Trp194 (4.13 Å), Phe369 (4.34 Å), Phe369 (4.14 Å), Pi–Alkyl: Cys200 (4.21 Å), Ala197 (4.32 Å), Cys200 (4.66 Å)
Quercetin	−10.50	−58.68	−42.09	−15.05	−2.24	−44.52	Conventional hydrogen bond: Tyr491 (2.45 Å), Glu377 (2.32 Å), Glu377 (2.16 Å), Tyr489 (1.79 Å), Cys200 (2.14 Å), Carbon hydrogen bond: Arg199 (2.65 Å), Glu377 (2.85 Å)	Pi–Sigma: Met355 (2.45 Å), Pi–Sulfur: Met355 (4.27 Å), Met355 (4.41 Å), Pi–Pi Stacked: Trp194 (4.29 Å), Phe369 (3.93 Å), Pi–Pi T–Shaped: Tyr491 (5.55 Å), Pi–Alkyl: Ala197 (4.42 Å), Arg199 (5.23 Å), Cys200 (5.44 Å), Ala197 (4.78 Å), Arg199 (4.40 Å), Ala197 (4.79 Å), Cys200 (4.47 Å)
Luteolin	−9.09	−40.07	−47.68	−17.48	−1.01	−32.84	Conventional hydrogen bond: Tyr491 (2.44 Å), Tyr489 (1.81 Å), Cys200 (2.01 Å), Carbon hydrogen bond: Arg199 (2.63 Å)	Pi–Sulfur: Met355 (4.27 Å), Met355 (4.41 Å), Pi–Pi Stacked: Trp194 (4.31 Å), Phe369 (3.98 Å), Pi–Pi T–Shaped: Tyr491 (5.47 Å), Pi–Alkyl: Ala197 (4.38 Å), Arg199 (5.19 Å), Ala197 (4.86 Å), Arg199 (4.39 Å), Ala197 (4.73 Å), Cys200 (4.41 Å)
Kaempferol	−8.14	−39.60	−25.84	−7.99	−0.75	−36.79	Conventional hydrogen bond: Ser242 (1.95 Å), Asn370 (2.30 Å), Carbon hydrogen bond: Gly371 (2.78 Å)	Pi–Donor hydrogen bond: Tyr489 (3.07 Å), Trp372 (2.38 Å), Pi–Pi Stacked: Trp194 (3.53 Å), Trp194 (3.92 Å), Trp194 (3.94 Å), Trp194 (5.27 Å), Phe369 (3.94 Å), Pi–Pi T–Shaped: Trp372 (5.07 Å), Trp372 (5.53 Å), Pi–Alkyl: Cys200 (4.91 Å), Ala197 (5.20 Å), Cys200 (4.60 Å)
Apigenin	−7.98	−36.73	−38.28	−13.40	−0.85	−34.03	Conventional hydrogen bond: Tyr491 (2.61 Å), Tyr489 (1.79 Å), Carbon hydrogen bond: Arg199 (2.67 Å)	Pi–Donor hydrogen bond: Trp194 (2.89 Å), Pi–Sigma: Met355 (2.46 Å), Pi–Sulfur: Met355 (4.32 Å), Met355 (4.38 Å), Pi–Pi Stacked: Trp194 (4.21 Å), Trp194 (5.69 Å), Phe369 (3.89 Å), Pi–Pi T–Shaped: Tyr491 (5.62 Å), Pi–Alkyl: Ala197 (4.31 Å), Arg199 (5.32 Å), Cys200 (5.34 Å), Ala197 (4.73 Å), Arg199 (4.41 Å), Ala197 (4.83 Å), Cys200 (4.57 Å)
Umbelliferone	−6.83	−25.99	−28.30	−9.06	−0.96	−23.40	Conventional hydrogen bond: Asn370 (1.73 Å)	Pi–Pi Stacked: Trp194 (4.04 Å), Trp194 (3.58 Å), Trp194 (5.43 Å), Trp194 (3.92 Å), Phe369 (3.80 Å), Phe369 (4.46 Å), Pi–Alkyl: Ala197 (5.08 Å), Cys200 (4.77 Å)
Verapamil	−6.08	−55.87	−60.12	−22.88	−1.57	−58.63	Salt bridge; Attractive charge: Glu377 (2.50 Å), Hydrogen bond: Trp372 (2.79 Å), Carbon hydrogen bond: Arg199 (3.07 Å), Glu377 (2.62 Å), Tyr373 (3.02 Å), Trp372 (2.80 Å)	Pi–Sigma: Gly202 (2.59 Å), Pi–Sulfur: Cys200 (3.99 Å), Pi–Pi T–Shaped: Phe369 (4.51 Å), Trp372 (5.53 Å), Alkyl: Met374 (4.92 Å), Met434 (3.95 Å), Pi–Alkyl: Trp194 (4.06 Å), Trp194 (3.66 Å), Trp194 (5.26 Å), Trp194 (3.58 Å), Trp194 (5.02 Å), Phe369 (3.51 Å), Trp372 (4.19 Å), Trp372 (4.16 Å), Trp372 (3.62 Å), Tyr489 (4.38 Å), Cys200 (5.19 Å)
Ferulic acid	−5.95	−26.82	−8.19	−0.33	−0.19	−33.16	Conventional hydrogen bond: Ser242 (2.73 Å)	Pi–Pi Stacked: Trp194 (3.49 Å), Trp194 (4.08 Å), Phe369 (4.55 Å), Alkyl: Leu209 (4.53 Å), Ile244 (4.44 Å), Pi–Alkyl: Trp194 (4.94 Å), Phe369 (4.29 Å), Tyr489 (4.49 Å), Cys200 (5.15 Å)
Voltage–dependent L–type calcium channel subunit alpha–1C (VGCAC1C)								
Rutin	−9.07	−57.33	−20.14	−5.52	−2.79	−45.06	Conventional hydrogen bond: Asn741 (1.69 Å), Ser1132 (1.73 Å), Glu1135 (2.21 Å), Gly1463 (3.00 Å), Glu363 (1.96 Å), Carbon hydrogen bond: Gly705 (2.98 Å), Asn741 (2.99 Å), Asn741 (2.79 Å)	Alkyl: Leu744 (4.82 Å), Leu745 (4.44 Å), Met1178 (5.29 Å), Pi–Alkyl: Phe748 (5.44 Å)
Quercetin	−7.58	−48.23	−40.37	−14.30	−1.05	−47.92	Conventional hydrogen bond Ile738 (2.57 Å), Asn741 (1.92 Å), Thr361 (1.90 Å), Carbon hydrogen bond: Ile738 (2.86 Å)	Pi–Pi T–Shaped: Phe737 (5.24 Å), Phe737 (5.29 Å), Alkyl: Ile738 (4.12 Å), Pi–Alkyl: Leu397 (4.87 Å), Ile360 (4.71 Å), Val396 (5.33 Å)
Apigenin	−6.12	−32.47	−24.50	−7.41	−0.78	−29.70	Conventional hydrogen bond: Ile360 (2.04 Å), Asn741 (2.71 Å)	Pi–Donor Hydrogen Bond: Tyr742 (3.29 Å), Pi–Alkyl: Val275 (5.46 Å), Val400 (5.07 Å), Ala272 (5.42 Å), Val400 (4.33 Å)
Luteolin	−5.78	−42.83	−16.03	−3.73	−2.57	−28.94	Conventional hydrogen bond: Asn741 (2.11 Å), Ile360 (1.84 Å), Glu1135 (1.90 Å), Glu1135 (2.55 Å), Thr1462 (3.04 Å), Gly1463 (1.98 Å)	Pi–Alkyl: Leu397 (4.90 Å), Met362 (5.05 Å)
Verapamil	−5.77	−56.89	−25.64	−7.91	−0.81	−48.66	Salt bridge; Attractive charge: Glu363 (3.12 Å), Glu1135 (2.41 Å), Attractive Charge: Glu706 (5.02 Å), Carbon hydrogen bond: Glu363 (2.60 Å), Glu1135 (2.71 Å), Thr704 (2.49 Å), Glu363 (2.65 Å), Glu1135 (2.73 Å), Asn741 (2.59 Å), Ile360 (2.69 Å), Ser1132 (2.80 Å), Ser1132 (2.86 Å)	Pi–Pi T–Shaped: Phe737 (5.70 Å), Tyr1508 (5.67 Å), Alkyl: Ala1512 (3.85 Å), Met362 (5.13 Å), Pi–Alkyl: Phe737 (4.51 Å), Phe1134 (4.76 Å), Tyr1508 (5.15 Å), Leu397 (4.71 Å)
Kaempferol	−5.75	−35.56	−30.99	−10.23	−1.59	−21.96	Conventional hydrogen bond: Asn741 (1.63 Å), Ile360 (1.80 Å), Thr704 (1.74 Å)	Pi–Pi T–Shaped: Phe737 (5.49 Å), Phe737 (5.20 Å), Pi–Alkyl: Leu397 (4.55 Å), Leu397 (4.59 Å)
Umbelliferone	−4.57	−23.01	−25.39	−7.80	−0.24	−23.76	Conventional hydrogen bond: Asn741 (2.86 Å), Carbon hydrogen bond: Thr704 (2.79 Å)	Pi–Alkyl: Leu744 (4.38 Å), Ala1174 (5.01 Å), Leu744 (4.24 Å), Ala1174 (5.25 Å)
Ferulic acid	−3.85	−17.57	−10.73	−1.43	0.00	−22.75	Carbon hydrogen bond: Ser393 (2.43 Å)	Pi–Alkyl: Phe737 (4.68 Å), Leu397 (4.70 Å)

∆GBinding: binding free energy; pKi: logarithm of inhibition constant (Ki); ∆GHbond: hydrogen bonding energy; ∆GvdW: Van der Waals force energy; these energies all contribute to binding free energy (∆GBinding).

## Data Availability

Data is contained within the article and Supplementary Material.

## Supplementary Materials

The following supporting information can be downloaded at: https://www.mdpi.com/article/10.3390/ph15121522/s1, Figure S1: HPLC DAD–UV/Vis chromatograms of *C. melo* seed extracts at different wavelengths and standard addition validation chromatograms of bioactive compounds; Figure S2. In jejunal tissue preparations, *C. melo* kernel sequential extracts and verapamil inhibit smooth muscle contraction; Figure S3. A. In tracheal tissue preparations, *C. melo* kernel sequential extracts and verapamil inhibit smooth muscle contraction; Figure S4. A. In urinary bladder tissue preparations, *C. melo* kernel sequential extracts and verapamil inhibit smooth muscle contraction; Figure S5. Functional enrichments of up-regulated and down-regulated DEGs of datasets. Figure S6 Identification of potential WGCNA hub-genes; Figure S7. The functional enrichment of top four WGCNA modules of GSE41649 and GSE15283; Figure S8. GSEA of GSE41649 for GO and KEGG functional enrichment; Figure S9. GSEA of GSE15823 for GO and KEGG functional enrichment; Figure S10. 2D ligand-protein interaction between bioactive compounds of *C. melo* seed sequential extracts and proteins; A. Interleukin 2 Receptor Subunit β (IL2RB) B. Nitric Oxide Synthase 2 (NOS2) C. Voltage-dependent L–type calcium channel subunit alpha 1C α; Table S1. Identification of phytoconstituents of *C. melo* seeds extracts with LC ESI-MS/MS; Table S2. Precision validation of analytical method *C. melo* seed extracts; Table S3. Accuracy validation of analytical method of *C. melo* seed extracts through % recovery method; Table S4. Network analysis of pathogenic target genes interaction with phytoconstituents; Table S5. The binding energies (kcal/mol) of docked ligand-protein complex calculated with Prime MM–GBSA.

## References

[1] Stern J., Pier J., Litonjua A.A. (2020). Asthma Epidemiology and Risk Factors. Semin. Immunopathol..

[2] Michaeloudes C., Abubakar–Waziri H., Lakhdar R., Raby K., Dixey P., Adcock I.M., Mumby S., Bhavsar P.K., Chung K.F. (2022). Molecular Mechanisms of Oxidative Stress in Asthma. Mol. Aspects Med..

[3] Chung K.F. (2013). New Treatments for Severe Treatment–Resistant Asthma: Targeting the Right Patient. Lancet Respir. Med..

[4] Lambrecht B.N., Hammad H., Fahy J.V. (2019). The Cytokines of Asthma. Immunity.

[5] Hellström P.M. (2019). Pathophysiology of the Irritable Bowel Syndrome—Reflections of Today. Best Pract. Res. Clin. Gastroenterol..

[6] Camilleri M., Sellin J.H., Barrett K.E. (2017). Pathophysiology, Evaluation, and Management of Chronic Watery Diarrhea. Gastroenterology.

[7] Anbazhagan A.N., Priyamvada S., Alrefai W.A., Dudeja P.K. (2018). Pathophysiology of IBD Associated Diarrhea. Tissue Barriers.

[8] Malysz J., Petkov G.V. (2020). Urinary Bladder Smooth Muscle Ion Channels: Expression, Function, and Regulation in Health and Disease. Am. J. Physiol. Physiol..

[9] Duke J.A. (2008). Melon (*Cucumis melo* L.). Duke’s Handbook of Medicinal Plants of the Bible.

[10] Silva M.A., Albuquerque T.G., Alves R.C., Oliveira M.B.P.P., Costa H.S. (2020). Melon (*Cucumis melo* L.) by–Products: Potential Food Ingredients for Novel Functional Foods?. Trends Food Sci. Technol..

[11] Mariod A.A., Saeed Mirghani M.E., Hussein I. (2017). Cucumis Melo Var. Cantalupo Cantaloupe. Unconv. Oilseeds Oil Sources.

[12] Erhirhie E., Ekene N. (2014). Medicinal Values on Citrullus Lanatus (Watermelon): Pharmacological Review. Int. J. Res. Pharm. Biomed. Sci..

[13] Siddiqui W.A., Shahzad M., Shabbir A., Ahmad A. (2018). Evaluation of Anti–Urolithiatic and Diuretic Activities of Watermelon (Citrullus Lanatus) Using in Vivo and in Vitro Experiments. Biomed. Pharmacother..

[14] Rahman H., Priyanka P., Lavanya T., Srilakshni N., Payili R.K. (2013). A Review on Ethnobotany, Phytochemisrty and Pharmacology Of. Int. Res. J. Pharm. Appl. Sci..

[15] Rimando A.M., Perkins–Veazie P.M. (2005). Determination of Citrulline in Watermelon Rind. J. Chromatogr. A.

[16] Reetu, Tomar M. (2017). Watermelon: A Valuable Horticultural Crop with Nutritional Benefits. Pop. Kheti.

[17] Gómez–García R., Campos D.A., Aguilar C.N., Madureira A.R., Pintado M. (2020). Valorization of Melon Fruit (*Cucumis melo* L.) by–Products: Phytochemical and Biofunctional Properties with Emphasis on Recent Trends and Advances. Trends Food Sci. Technol..

[18] Asif H.M., Akhtar N., Sultana S., Rehman S.U., Akram M., Rehman U.J. (2014). Medicinal Properties of Cucumis Melo Linn. J. Pharm. Pharm. Sci..

[19] Patel S., Rauf A. (2017). Edible Seeds from Cucurbitaceae Family as Potential Functional Foods: Immense Promises, Few Concerns. Biomed. Pharmacother..

[20] Salehi B., Capanoglu E., Adrar N., Catalkaya G., Shaheen S., Jaffer M., Giri L., Suyal R., Jugran A.K., Calina D. (2019). Cucurbits Plants: A Key Emphasis to Its Pharmacological Potential. Molecules.

[21] Dixit Y., Kar A. (2010). Protective Role of Three Vegetable Peels in Alloxan Induced Diabetes Mellitus in Male Mice. Plant Foods Hum. Nutr..

[22] Wahid M., Ali A., Saqib F., Aleem A., Bibi S., Afzal K., Ali A., Baig A., Khan S.A., Bin Asad M.H.H. (2020). Pharmacological Exploration of Traditional Plants for the Treatment of Neurodegenerative Disorders. Phyther. Res..

[23] Wahid M., Saqib F., Akhtar S., Ali A., Wilairatana P., Mubarak M.S. (2022). Possible Mechanisms Underlying the Antispasmodic, Bronchodilator, and Antidiarrheal Activities of Polarity–Based Extracts of Cucumis Sativus L. Seeds in In Silico, In Vitro, and In Vivo Studies. Pharmaceuticals.

[24] Wahid M., Saqib F. (2022). Scientific Basis for Medicinal Use of Citrullus Lanatus (Thunb.) in Diarrhea and Asthma: In Vitro, in Vivo and in Silico Studies. Phytomedicine.

[25] Sirous H., Chemi G., Campiani G., Brogi S. (2019). An Integrated in Silico Screening Strategy for Identifying Promising Disruptors of P53–MDM2 Interaction. Comput. Biol. Chem..

[26] Kuhn B., Kollman P.A. (2000). Binding of a Diverse Set of Ligands to Avidin and Streptavidin: An Accurate Quantitative Prediction of Their Relative Affinities by a Combination of Molecular Mechanics and Continuum Solvent Models. J. Med. Chem..

[27] Du X., Li Y., Xia Y.-L., Ai S.-M., Liang J., Sang P., Ji X.-L., Liu S.-Q. (2016). Insights into Protein–Ligand Interactions: Mechanisms, Models, and Methods. Int. J. Mol. Sci..

[28] Yu X. (2021). Prediction of Inhibitory Constants of Compounds against SARS–CoV 3CLpro Enzyme with 2D–QSAR Model. J. Saudi Chem. Soc..

[29] Soltani R., Hashemi M., Farazmand A., Asghari G., Heshmat–Ghahdarijani K., Kharazmkia A., Ghanadian S.M. (2017). Evaluation of the Effects of Cucumis Sativus Seed Extract on Serum Lipids in Adult Hyperlipidemic Patients: A Randomized Double–Blind Placebo–Controlled Clinical Trial. J. Food Sci..

[30] Rajasree R.S., Sibi P.I., Francis F., William H. (2016). Phytochemicals of Cucurbitaceae Family—A Review. Int. J. Pharmacogn. Phytochem. Res..

[31] Mallik J., Priyanka D., Sourav D. (2013). Pharmacological Activity of Cucumis Sativus L.—A Complete Review. Asian J. Pharm. Res. Dev..

[32] Mukherjee P.K., Nema N.K., Maity N., Sarkar B.K. (2013). Phytochemical and Therapeutic Potential of Cucumber. Fitoterapia.

[33] Yang C., Zhang S.S., Li X.L., Wang Z.F., Zhao L.Q. (2015). Inhibitory Effect of TongXie–YaoFang Formula on Colonic Contraction in Rats. World J. Gastroenterol..

[34] Mahn K., Ojo O.O., Chadwick G., Aaronson P.I., Ward J.P.T., Lee T.H. (2010). Ca^2+^ Homeostasis and Structural and Functional Remodelling of Airway Smooth Muscle in Asthma. Thorax.

[35] Athari S.S. (2019). Targeting Cell Signaling in Allergic Asthma. Signal Transduct. Target. Ther..

[36] Chen L.W., Ko W.C. (2021). Suppressive Effects of Rutin, Quercitrin, and Isoquercitrin on Atypical Allergic Asthma in an Animal Model. Med. Drug Discov..

[37] Liu L.L., Zhang Y., Zhang X.F., Li F.H. (2018). Influence of Rutin on the Effects of Neonatal Cigarette Smoke Exposure–Induced Exacerbated MMP–9 Expression, Th17 Cytokines and NF–KB/INOS–Mediated Inflammatory Responses in Asthmatic Mice Model. Korean J. Physiol. Pharmacol..

[38] Hall J.E., Hall M.E. (2020). Excitation and Contraction of Smooth Muscle. Guyton and Hall Textbook of Medical Physiology.

[39] Sharkey K.A., MacNaughton W.K., Brunton L.L., Hilal-Dandan R., Knollmann B.C. (2018). Gastrointestinal Motility and Water Flux, Emesis, and Biliary and Pancreatic Disease. Goodman and Gilman’s The Pharmacological Basis of Therapeutics.

[40] Gilani A.H., Ghayur M.N., Saify Z.S., Ahmed S.P., Choudhary M.I., Khalid A. (2004). Presence of Cholinomimetic and Acetylcholinesterase Inhibitory Constituents in Betel Nut. Life Sci..

[41] Saqib F., Janbaz K.H. (2016). Rationalizing Ethnopharmacological Uses of Alternanthera Sessilis: A Folk Medicinal Plant of Pakistan to Manage Diarrhea, Asthma and Hypertension. J. Ethnopharmacol..

[42] Wallace J.L., Sharkey K.A., Brunton L.L., Hilal-Dandan R., Knollmann B.C. (2018). Pharmacotherapy of Gastric Acidity, Peptic Ulcers, and Gastroesophageal Reflux Disease. Goodman and Gilman’s The Pharmacological Basis of Therapeutics.

[43] Saqib F., Janbaz K.H. (2021). Ethnopharmacological Basis for Folkloric Claims of Anagallis Arvensis Linn. (Scarlet Pimpernel) as Prokinetic, Spasmolytic and Hypotensive in Province of Punjab, Pakistan. J. Ethnopharmacol..

[44] Gilani A.H., Jabeen Q., Ghayur M.N., Janbaz K.H., Akhtar M.S. (2005). Studies on the Antihypertensive, Antispasmodic, Bronchodilator and Hepatoprotective Activities of the Carum Copticum Seed Extract. J. Ethnopharmacol..

[45] Janbaz K.H., Nisa M., Saqib F., Imran I., Zia-Ul-Haq M., De Feo V. (2013). Bronchodilator, Vasodilator and Spasmolytic Activities of Methanolic Extract of Myrtus Communis L.. J. Physiol. Pharmacol..

[46] Miyazaki E., Yabu H., Sunano S. (1971). Excitation and Contraction of the Smooth Muscle. Jpn. J. Smooth Muscle Res..

[47] Janbaz K.H., Zaeem Ahsan M., Saqib F., Imran I., Zia-Ul-Haq M., Abid Rashid M., Jaafar H.Z.E., Moga M. (2015). Scientific Basis for Use of Pyrus Pashia Buch.-Ham. Ex D. Don. Fruit in Gastrointestinal, Respiratory and Cardiovascular Ailments. PLoS ONE.

[48] Chan H.J., Ji Y.L., Chul H.C., Chang J.K. (2007). Anti-Asthmatic Action of Quercetin and Rutin in Conscious Guinea-Pigs Challenged with Aerosolized Ovalbumin. Arch. Pharm. Res..

[49] Agbor G.A., Longo F., Makong E.A., Tarkang P.A. (2014). Evaluation of the Antidiarrheal and Antioxidant Properties of Justicia Hypocrateriformis. Pharm. Biol..

[50] Iwao I., Terada Y. (1962). On the Mechanism of Diarrhea Due to Castor Oil. Jpn. J. Pharmacol..

[51] Atta A.H., Mouneir S.M. (2004). Antidiarrhoeal Activity of Some Egyptian Medicinal Plant Extracts. J. Ethnopharmacol..

[52] Gaginella T.S., Phillips S.F. (1975). Ricinoleic Acid: Current View of an Ancient Oil. Am. J. Dig. Dis..

[53] Yakubu M.T., Salimon S.S. (2015). Antidiarrhoeal Activity of Aqueous Extract of Mangifera Indica L. Leaves in Female Albino Rats. J. Ethnopharmacol..

[54] Reynolds I.J., Gould R.J., Snyder S.H. (1984). Loperamide: Blockade of Calcium Channels as a Mechanism for Antidiarrheal Effects. J. Pharmacol. Exp. Ther..

[55] Crowe A., Wong P. (2003). Potential Roles of P–Gp and Calcium Channels in Loperamide and Diphenoxylate Transport. Toxicol. Appl. Pharmacol..

[56] Wahid M., Saqib F., Qamar M., Ziora Z.M. (2022). Antispasmodic Activity of the Ethanol Extract of Citrullus Lanatus Seeds: Justifying Ethnomedicinal Use in Pakistan to Treat Asthma and Diarrhea. J. Ethnopharmacol..

[57] Wahid M., Saqib F., Ahmedah H.T., Gavris C.M., De Feo V., Hogea M., Moga M., Chicea R. (2021). Cucumis Sativus l. Seeds Ameliorate Muscular Spasm-Induced Gastrointestinal and Respiratory Disorders by Simultaneously Inhibiting Calcium Mediated Signaling Pathway. Pharmaceuticals.

[58] Rowan A.N. (1979). Guide for the Care and Use of Laboratory Animals.

[59] Elasoru S.E., Rhana P., de Oliveira Barreto T., Naves de Souza D.L., Menezes-Filho J.E.R., Souza D.S., Loes Moreira M.V., Gomes Campos M.T., Adedosu O.T., Roman-Campos D. (2021). Andrographolide Protects against Isoproterenol-Induced Myocardial Infarction in Rats through Inhibition of L-Type Ca^2+^ and Increase of Cardiac Transient Outward K^+^ Currents. Eur. J. Pharmacol..

[60] Chauhan V., Singh M.P. (2020). Immuno-Informatics Approach to Design a Multi-Epitope Vaccine to Combat Cytomegalovirus Infection. Eur. J. Pharm. Sci..

[61] Yu Z., Kang L., Zhao W., Wu S., Ding L., Zheng F., Liu J., Li J. (2021). Identification of Novel Umami Peptides from Myosin via Homology Modeling and Molecular Docking. Food Chem..

[62] Subhani S., Jayaraman A., Jamil K. (2015). Homology Modelling and Molecular Docking of MDR1 with Chemotherapeutic Agents in Non-Small Cell Lung Cancer. Biomed. Pharmacother..

