# Supplementary material for: Integrated Mechanisms of Polarity–Based Extracts of Cucumis melo L. Seed Kernels for Airway Smooth Muscle Relaxation via Key Signaling Pathways Based on WGCNA, In Vivo, and In Vitro Analyses
Source: Pharmaceuticals (Basel). 2022 Dec 7;15(12):1522. doi: 10.3390/ph15121522 (PMC9784679; doi:10.3390/ph15121522)
Supplement: Supplementary file 1 [file pharmaceuticals-15-01522-s001.zip › pharmaceuticals-1987324-supplementary.pdf]

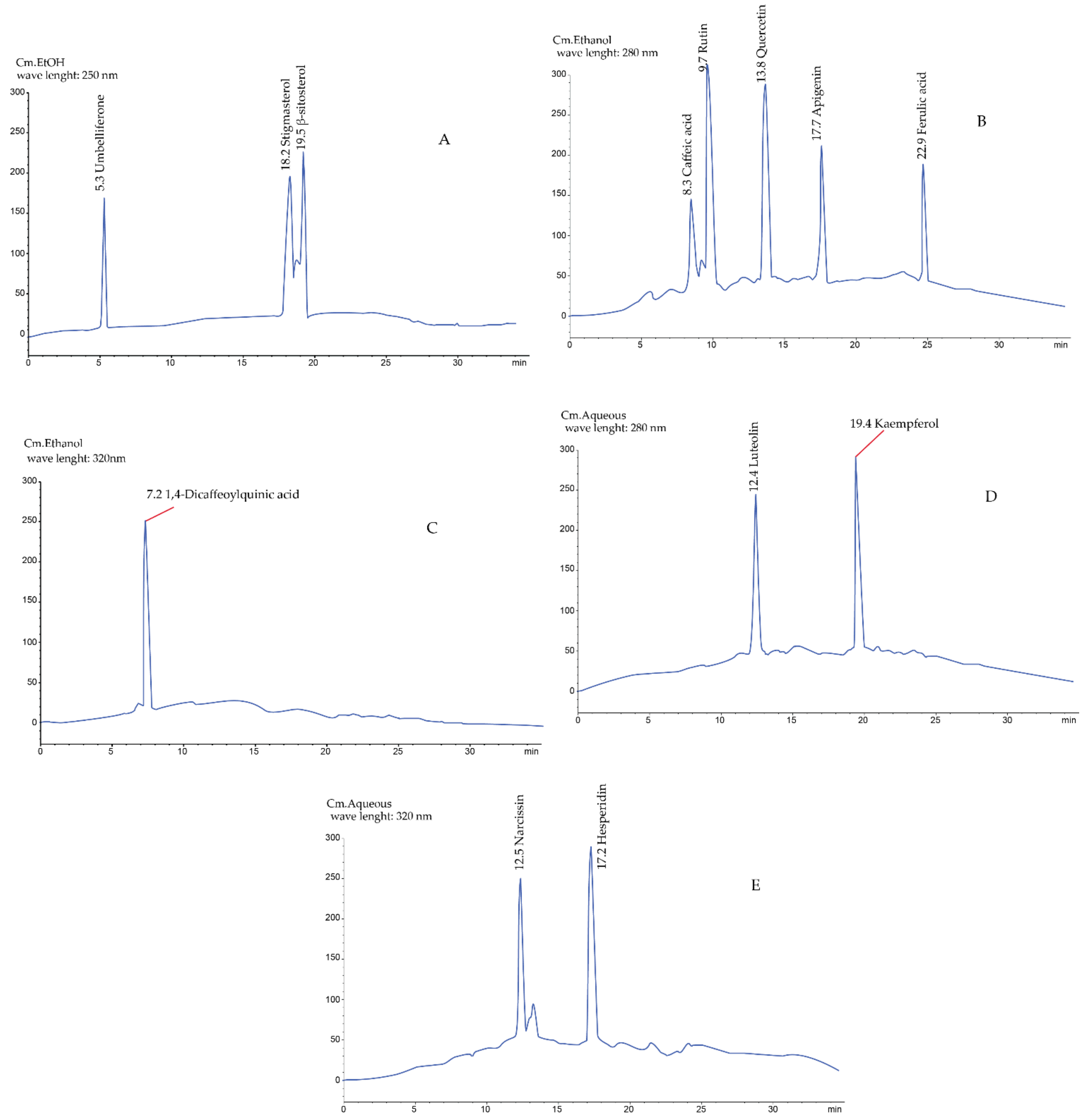

Figure S1. HPLC DAD-UV/Vis chromatograms of a *C. melo* seed extracts at different wavelengths and standard addition validation chromatograms of bioactive compounds.

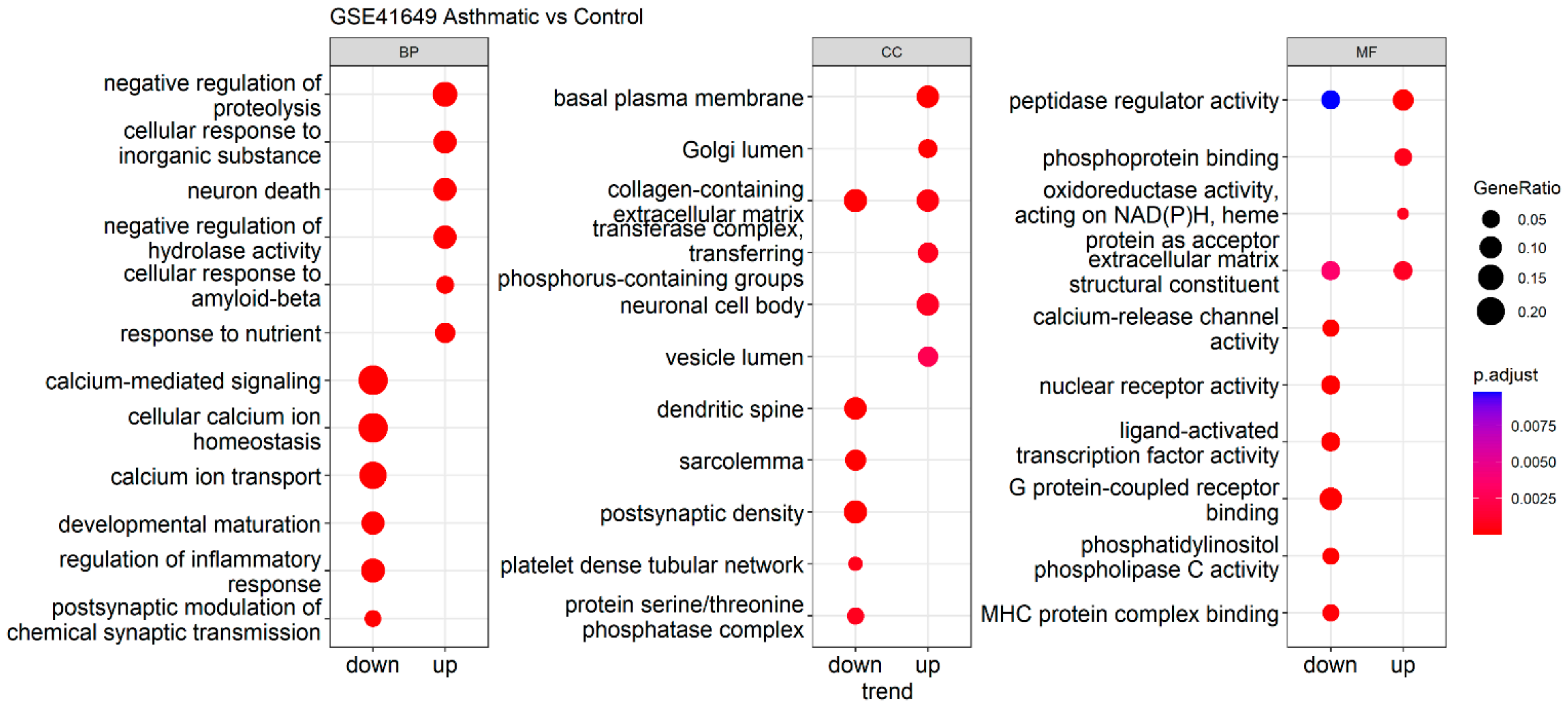

GSE41649 Asthmatic vs Control
BP
CC
MF
negative regulation of proteolysis
cellular response to inorganic substance
neuron death
negative regulation of hydrolase activity
cellular response to amyloid-beta
response to nutrient
calcium-mediated signaling
cellular calcium ion homeostasis
calcium ion transport
developmental maturation
regulation of inflammatory response
postsynaptic modulation of chemical synaptic transmission
basal plasma membrane
Golgi lumen
collagen-containing extracellular matrix
transferase complex, transferring phosphorus-containing groups
neuronal cell body
vesicle lumen
dendritic spine
sarcolemma
postsynaptic density
platelet dense tubular network
protein serine/threonine phosphatase complex
peptidase regulator activity
phosphoprotein binding
oxidoreductase activity, acting on NAD(P)H, heme protein as acceptor
extracellular matrix structural constituent
calcium-release channel activity
nuclear receptor activity
ligand-activated transcription factor activity
G protein-coupled receptor binding
phosphatidylinositol phospholipase C activity
MHC protein complex binding
down
up
trend
GeneRatio
0.05
0.10
0.15
0.20
p.adjust
0.0075
0.0050
0.0025

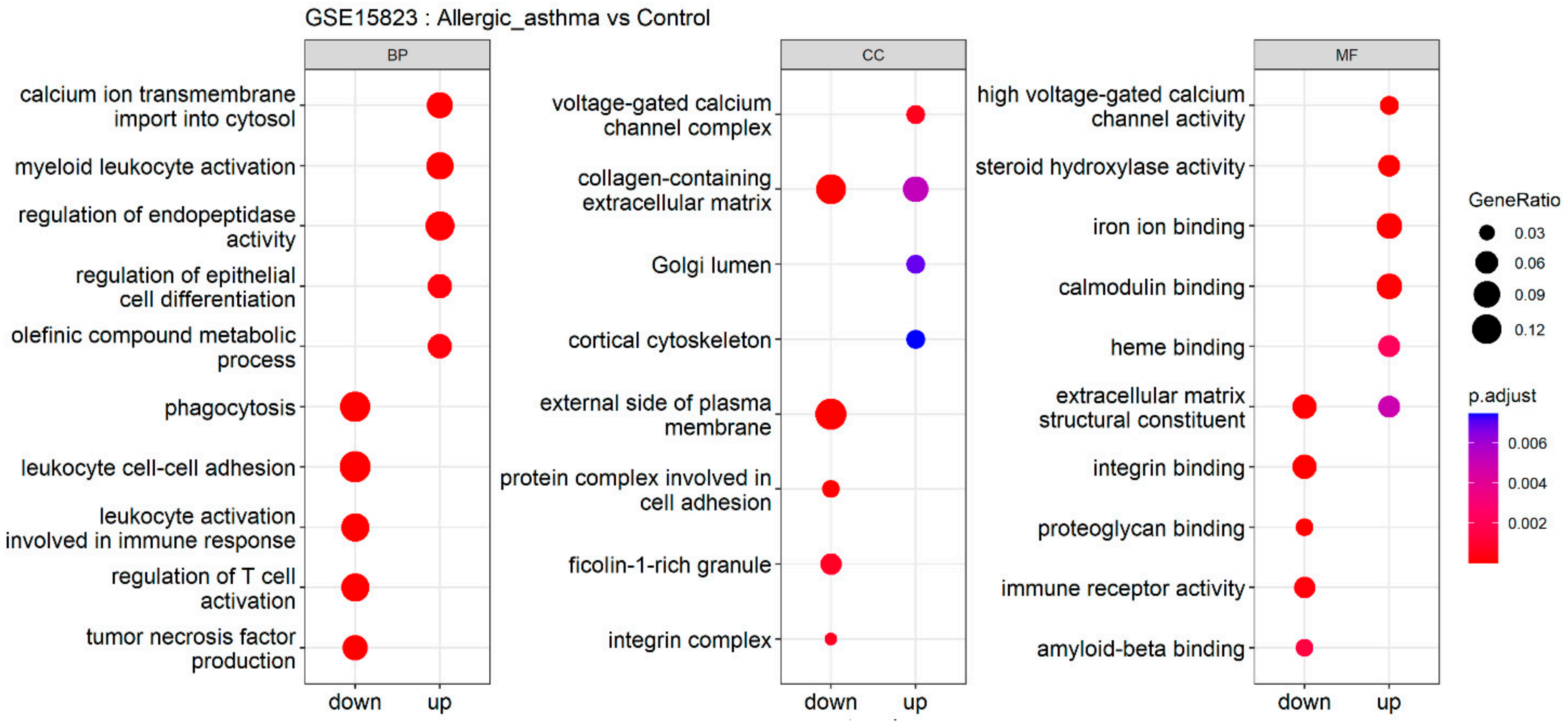
GSE15823 : Allergic_asthma vs Control
BP
calcium ion transmembrane import into cytosol
myeloid leukocyte activation
regulation of endopeptidase activity
regulation of epithelial cell differentiation
olefinic compound metabolic process
phagocytosis
leukocyte cell-cell adhesion
leukocyte activation involved in immune response
regulation of T cell activation
tumor necrosis factor production
down
up
CC
voltage-gated calcium channel complex
collagen-containing extracellular matrix
Golgi lumen
cortical cytoskeleton
external side of plasma membrane
protein complex involved in cell adhesion
ficolin-1-rich granule
integrin complex
down
up
MF
high voltage-gated calcium channel activity
steroid hydroxylase activity
iron ion binding
calmodulin binding
heme binding
extracellular matrix structural constituent
integrin binding
proteoglycan binding
immune receptor activity
amyloid-beta binding
down
up
GeneRatio
0.03
0.06
0.09
0.12
p.adjust
0.006
0.004
0.002

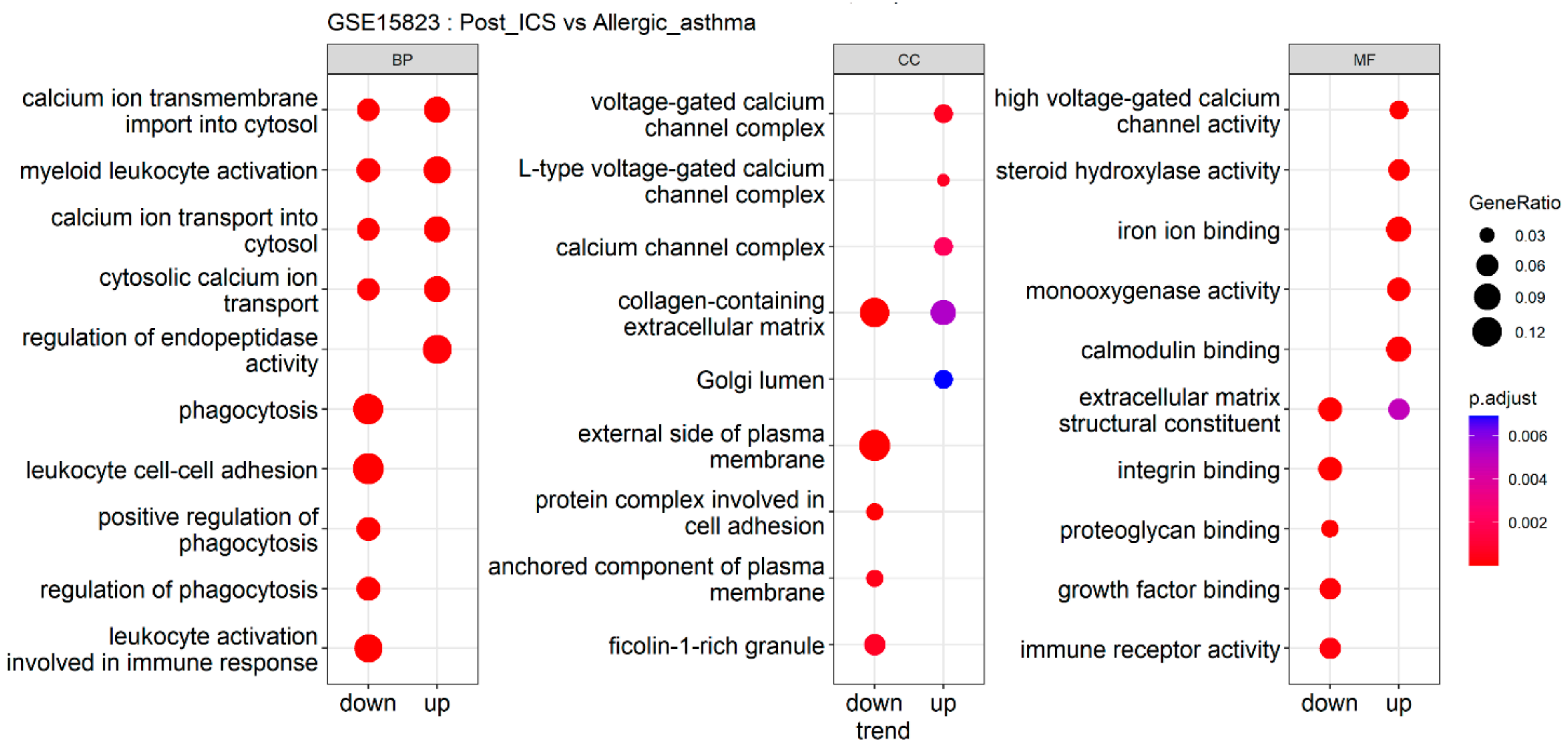

A. GO enrichment of differentially expressed genes

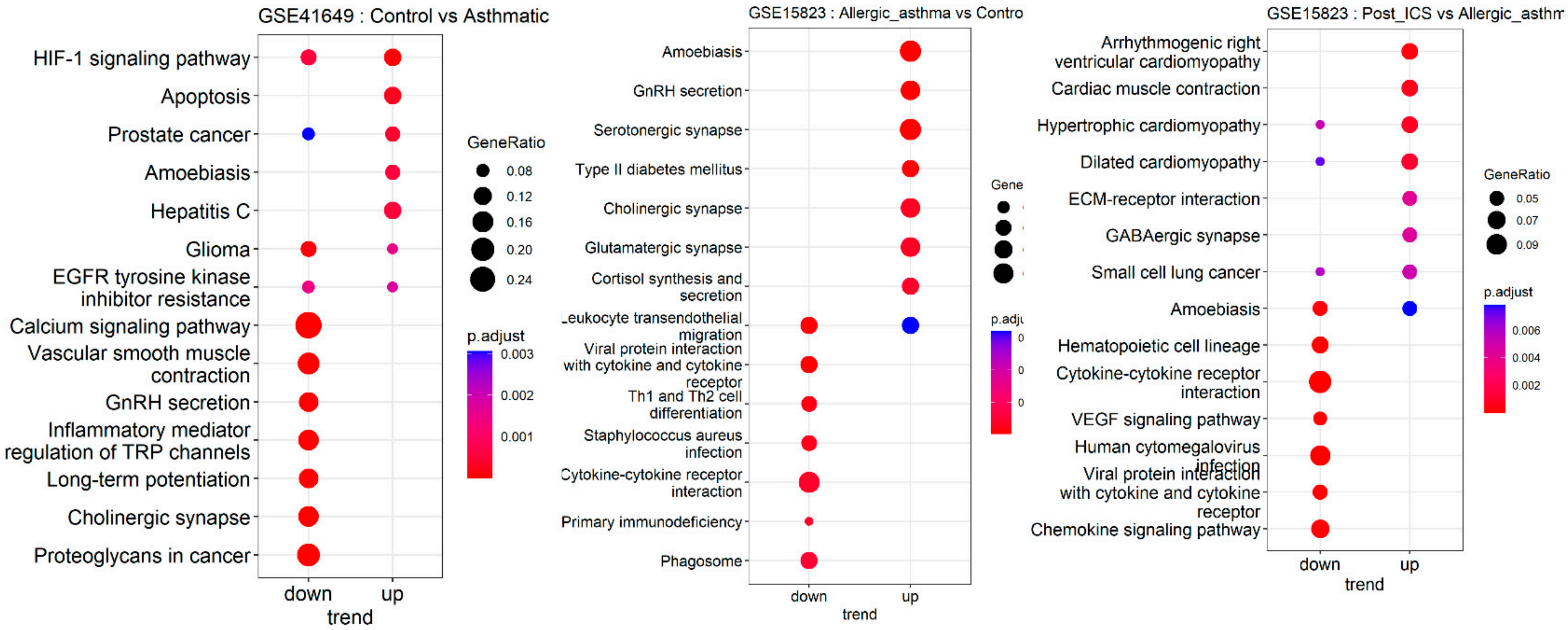

B. KEGG pathways enrichment of differentially expressed genes

Figure S2. Functional enrichments of up-regulated and down-regulated DEGs of datasets. **A.** Gene Ontology. **B.** KEGG pathways enrichment**.** G.O.: Gene Ontology; B.P.: Biological process; CC: cellular compartment; M.F.: molecular functions

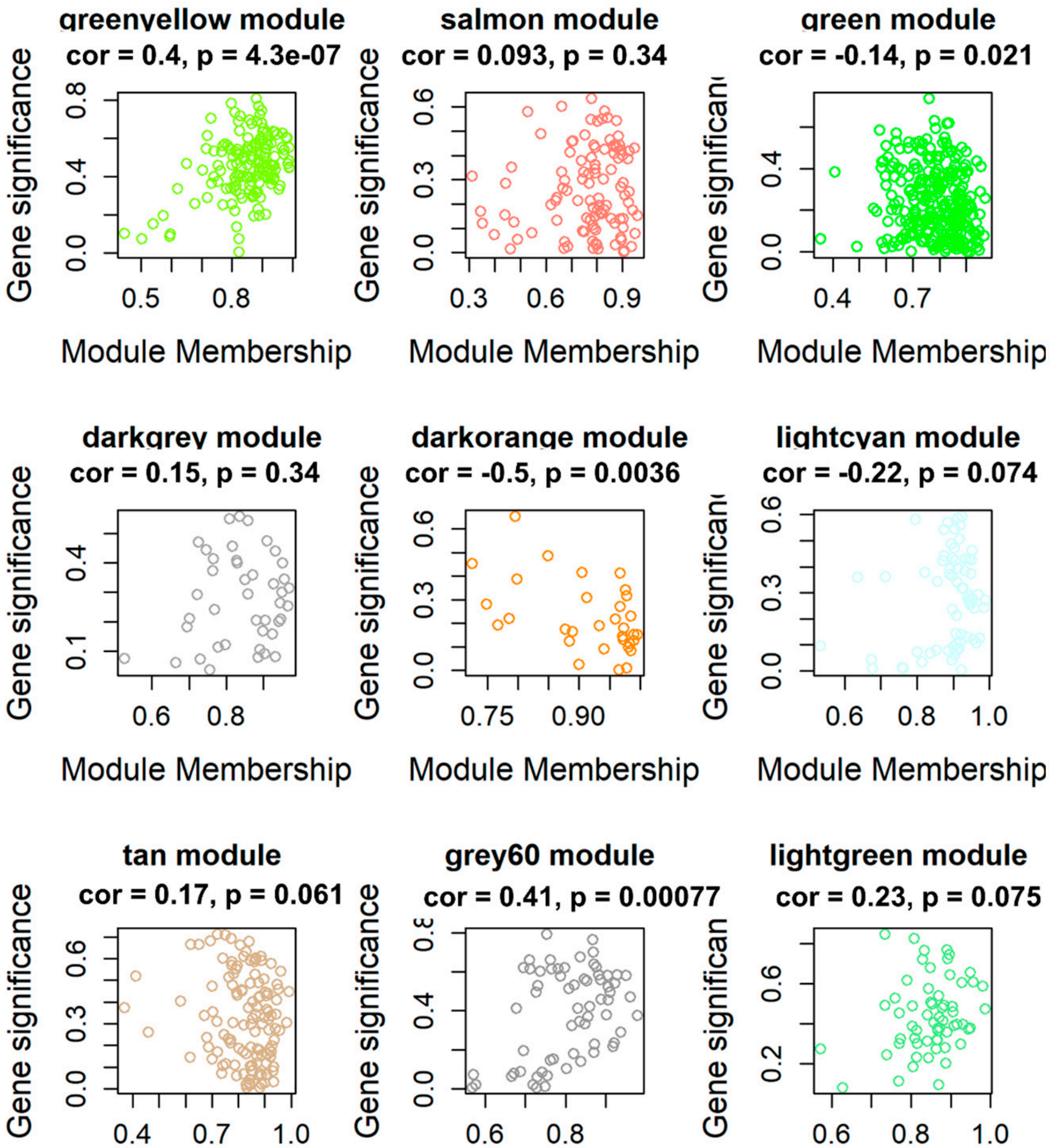
greenyellow module
cor = 0.4, p = 4.3e-07
salmon module
cor = 0.093, p = 0.34
green module
cor = -0.14, p = 0.021
darkgrey module
cor = 0.15, p = 0.34
darkorange module
cor = -0.5, p = 0.0036
lightcyan module
cor = -0.22, p = 0.074
tan module
cor = 0.17, p = 0.061
grey60 module
cor = 0.41, p = 0.00077
lightgreen module
cor = 0.23, p = 0.075
Gene significance
Module Membership

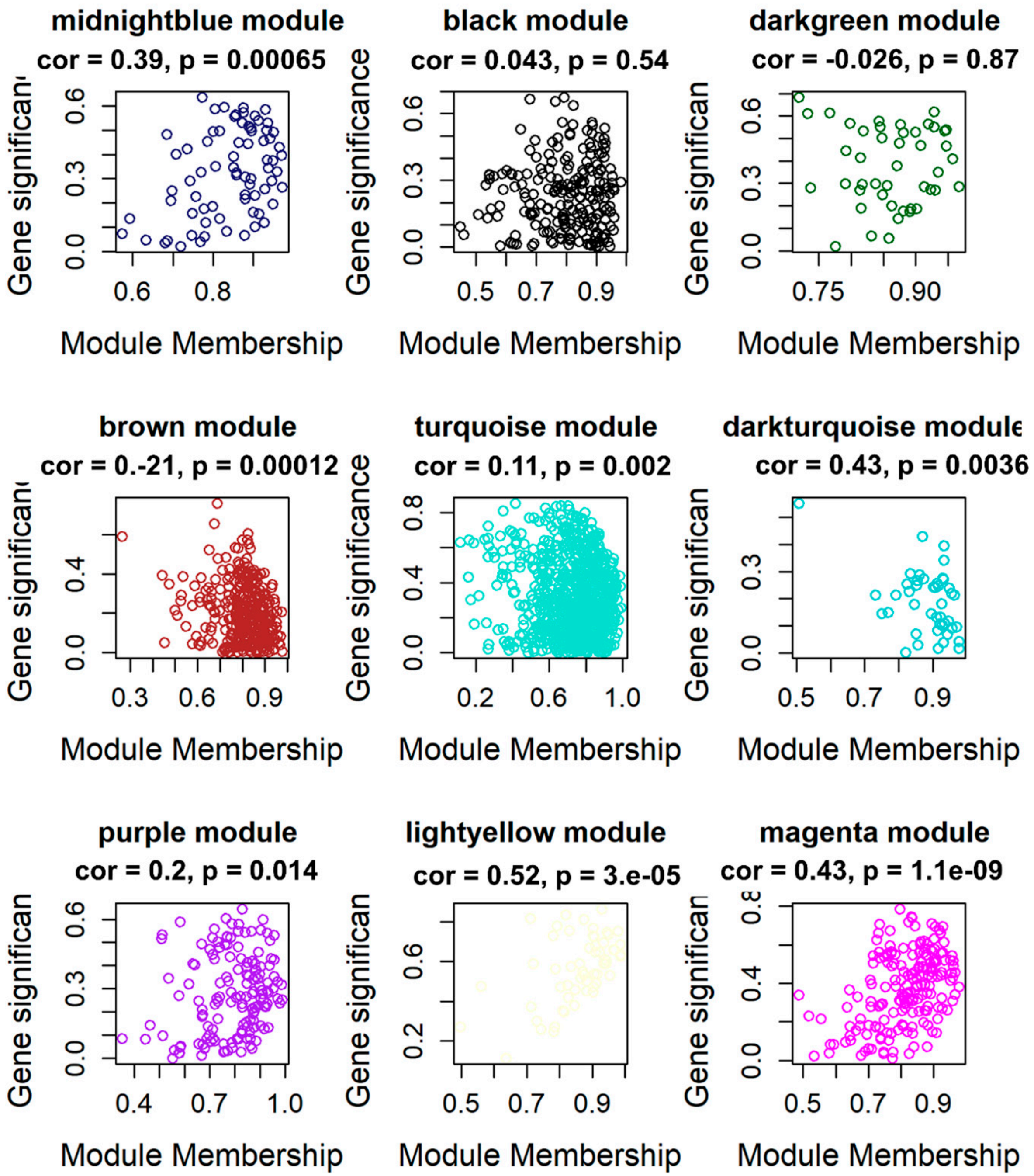

midnightblue module
cor = 0.39, p = 0.00065
black module
cor = 0.043, p = 0.54
darkgreen module
cor = -0.026, p = 0.87
brown module
cor = 0.-21, p = 0.00012
turquoise module
cor = 0.11, p = 0.002
darkturquoise module
cor = 0.43, p = 0.0036
purple module
cor = 0.2, p = 0.014
lightyellow module
cor = 0.52, p = 3.e-05
magenta module
cor = 0.43, p = 1.1e-09
Gene significance
Module Membership

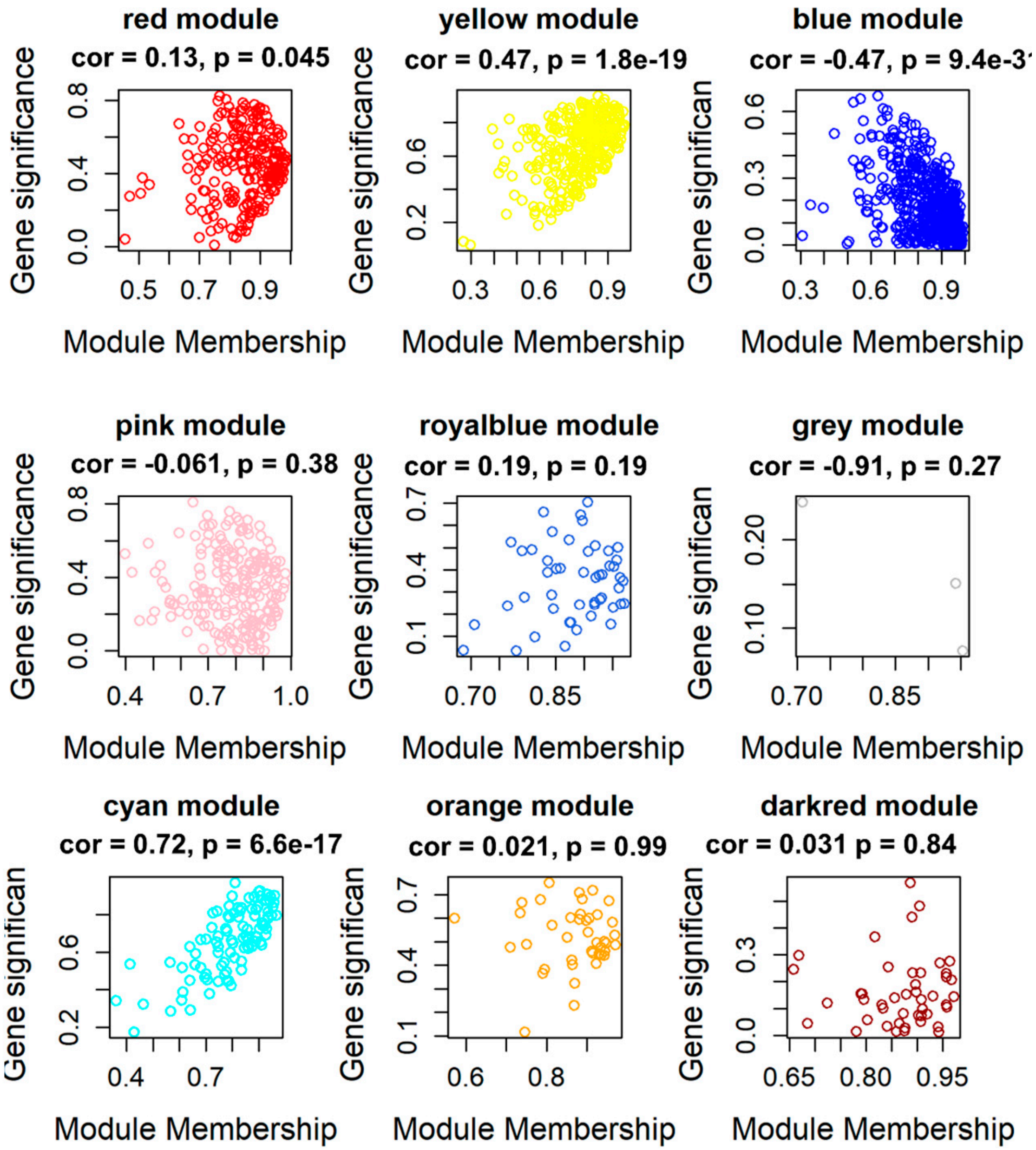

A. Identification potential WGCNA modules of GSE41649

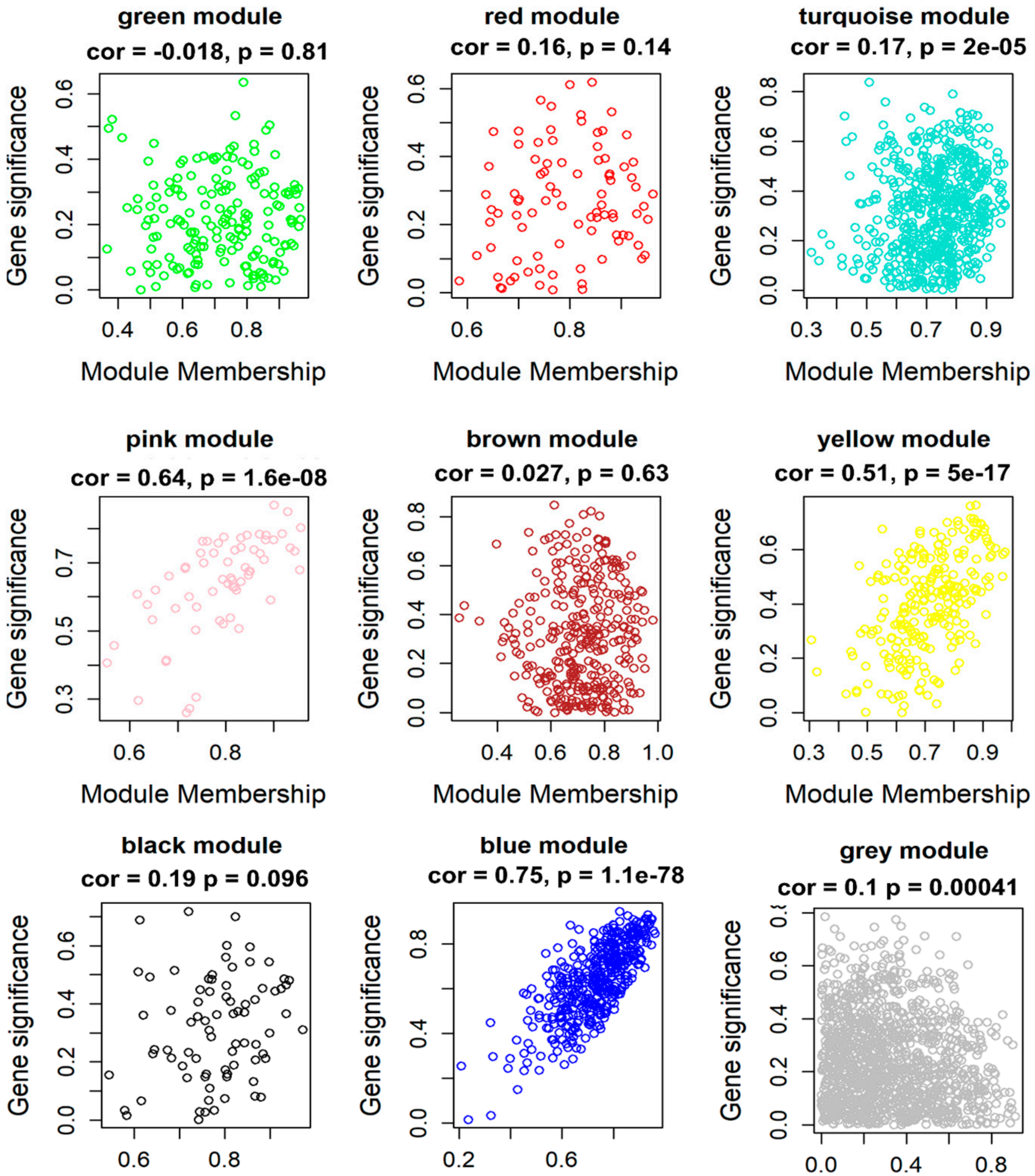

green module
cor = -0.018, p = 0.81
red module
cor = 0.16, p = 0.14
turquoise module
cor = 0.17, p = 2e-05
pink module
cor = 0.64, p = 1.6e-08
brown module
cor = 0.027, p = 0.63
yellow module
cor = 0.51, p = 5e-17
black module
cor = 0.19 p = 0.096
blue module
cor = 0.75, p = 1.1e-78
grey module
cor = 0.1 p = 0.00041
Gene significance
Module Membership

Figure S3 Identification of potential WGCNA hub-genes.

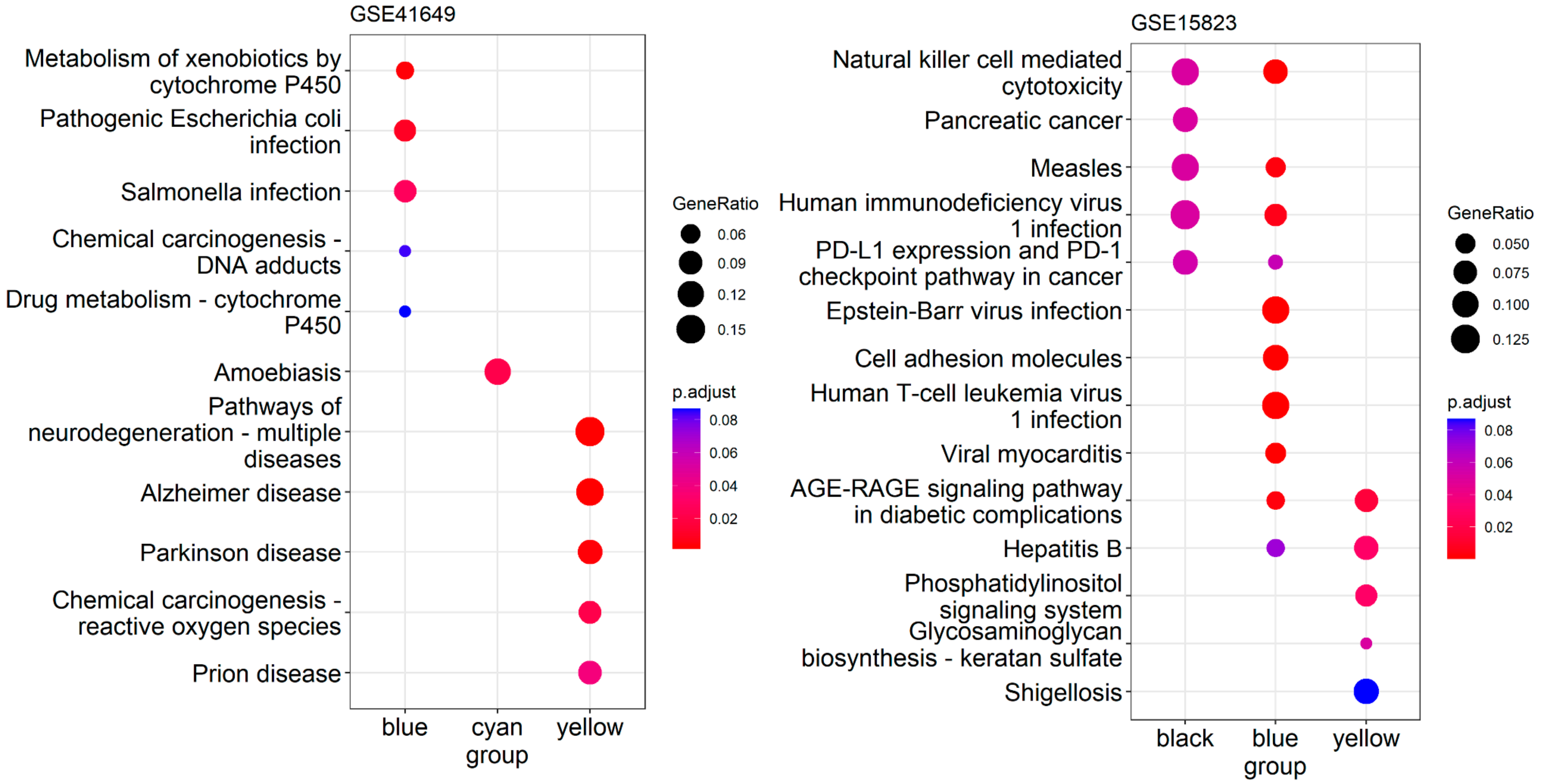

B. KEGG pathways enrichment of top 4 modules

Figure S4. The functional enrichment of top 4 WGCNA modules of GSE41649 and GSE15283. **A.** Gene Ontology (G.O.) enrichment in biological process (B.P.), cellular compartment (CC), and molecular functions (M.F.). **B.** KEGG pathways enrichment.

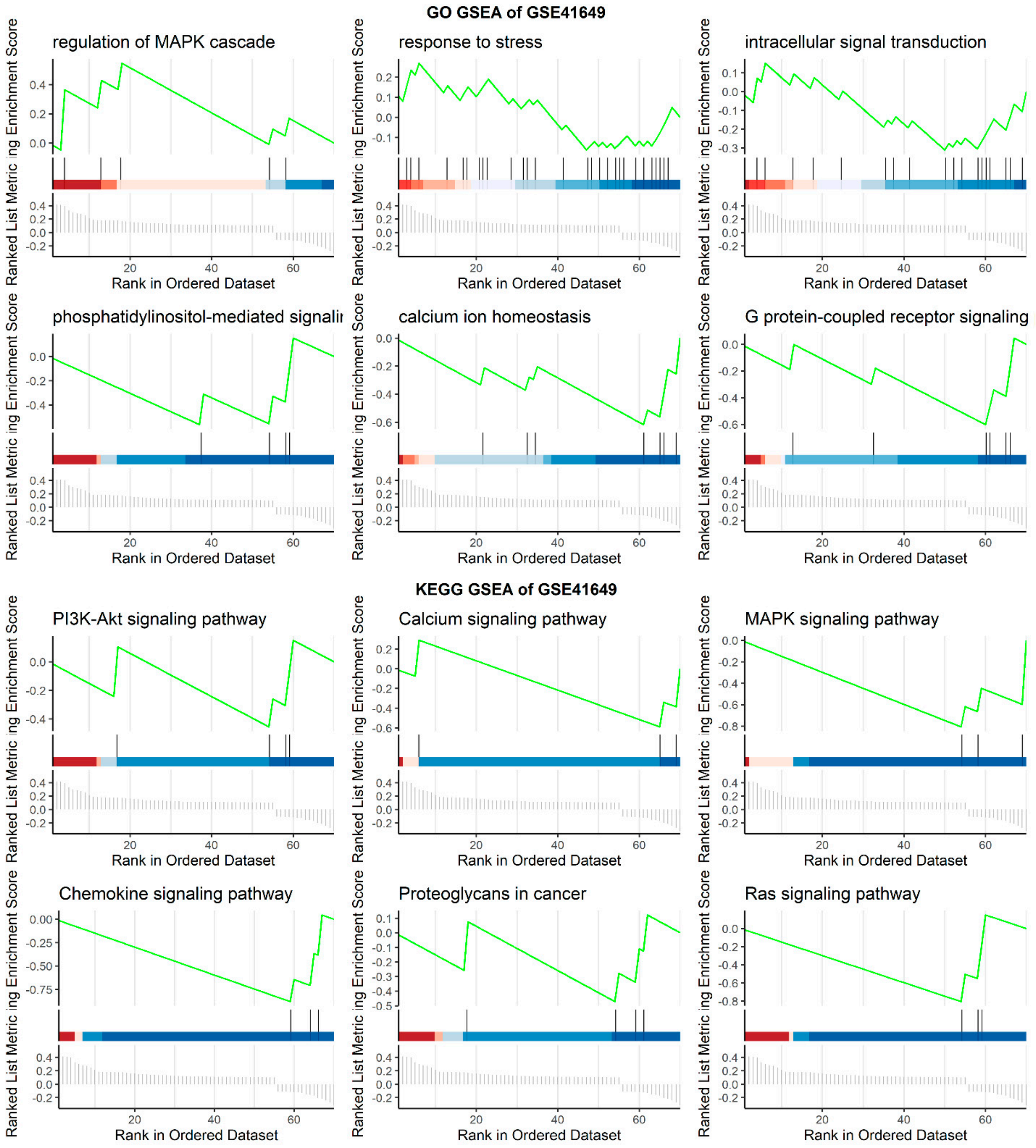
GO GSEA of GSE41649
regulation of MAPK cascade
response to stress
intracellular signal transduction
phosphatidylinositol-mediated signalir
calcium ion homeostasis
G protein-coupled receptor signaling
KEGG GSEA of GSE41649
PI3K-Akt signaling pathway
Calcium signaling pathway
MAPK signaling pathway
Chemokine signaling pathway
Proteoglycans in cancer
Ras signaling pathway
Ranked List Metric ing Enrichment Score
Rank in Ordered Dataset

Figure S5. GSEA of GSE41649 for GO and KEGG functional enrichment.

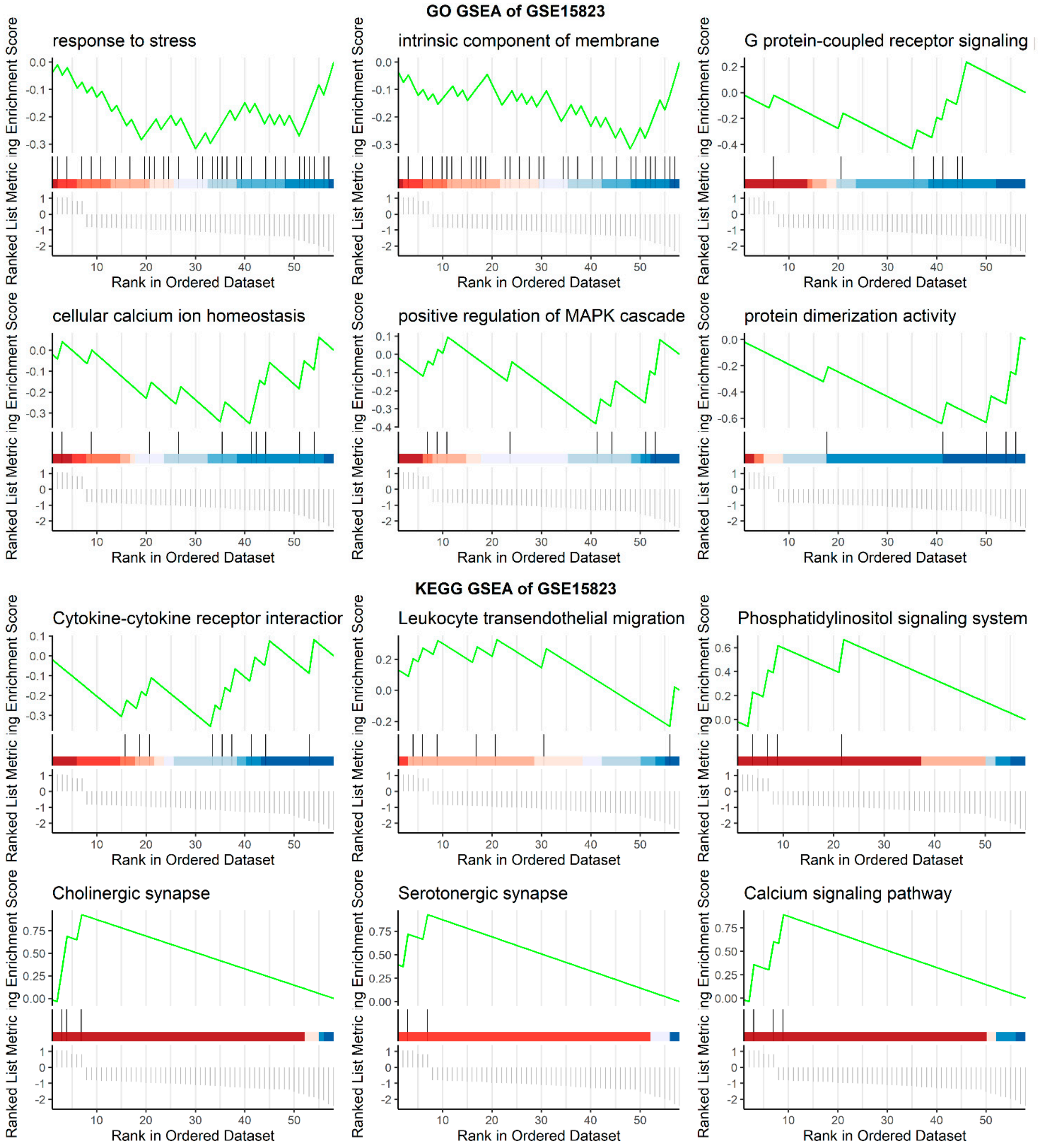
GO GSEA of GSE15823
response to stress
intrinsic component of membrane
G protein-coupled receptor signaling
cellular calcium ion homeostasis
positive regulation of MAPK cascade
protein dimerization activity
KEGG GSEA of GSE15823
Cytokine-cytokine receptor interactior
Leukocyte transendothelial migration
Phosphatidylinositol signaling system
Cholinergic synapse
Serotonergic synapse
Calcium signaling pathway
ing Enrichment Score
Ranked List Metric
Rank in Ordered Dataset

Figure S6. GSEA of GSE15823 for GO and KEGG functional enrichment**.**

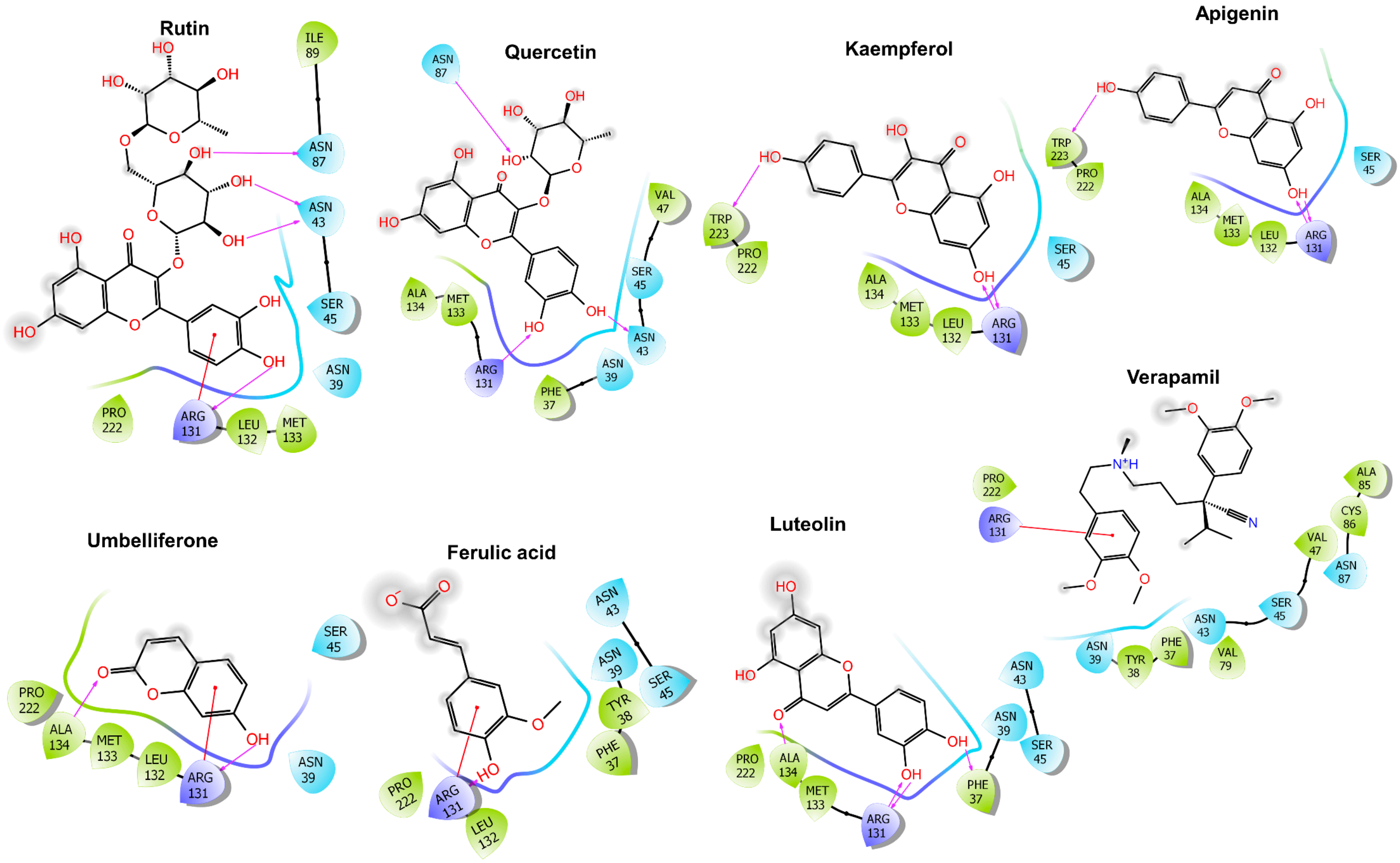

A. Interleukin 2 Receptor Subunit β (IL2RB)

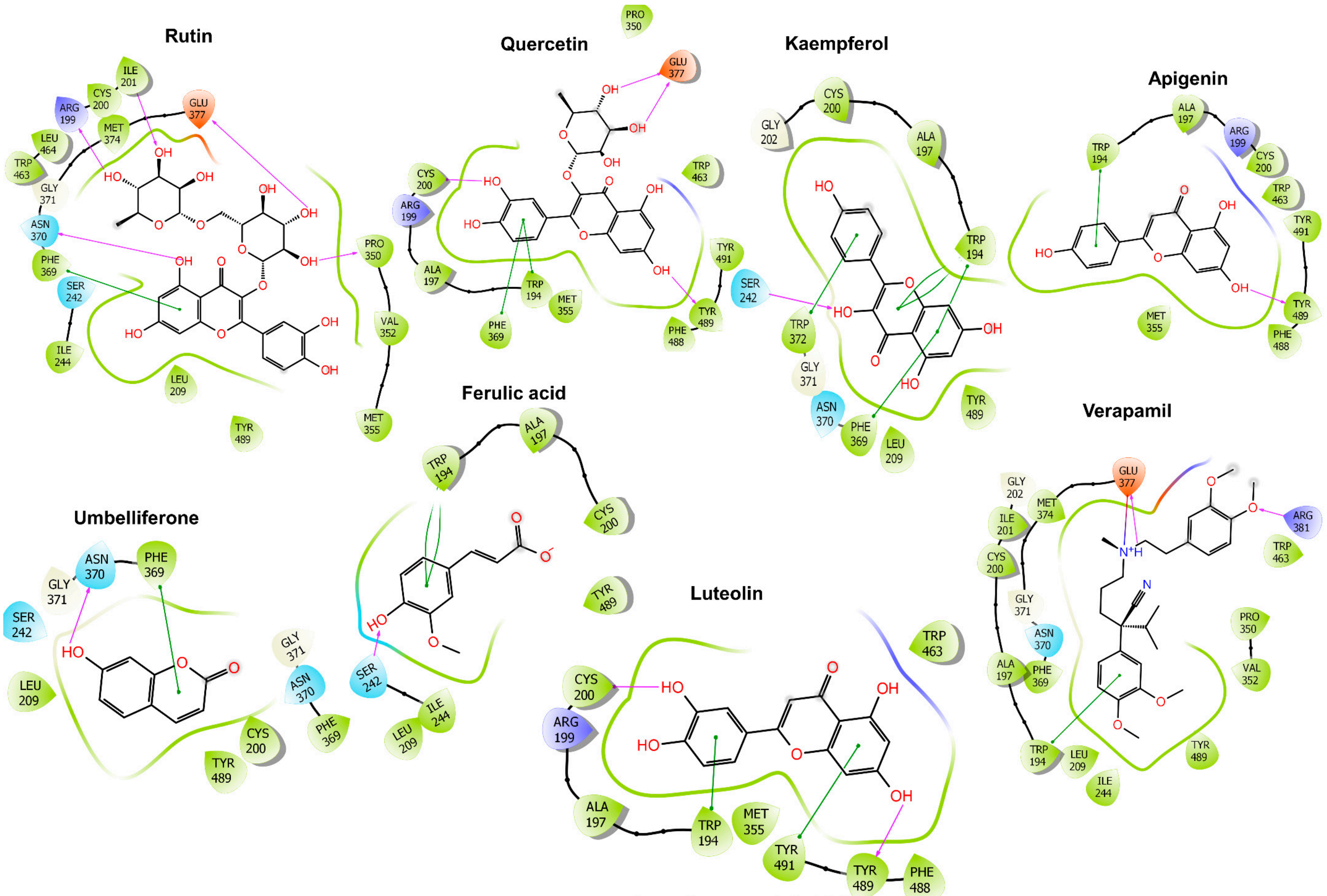

B. Nitric Oxide Synthase 2 (NOS2)

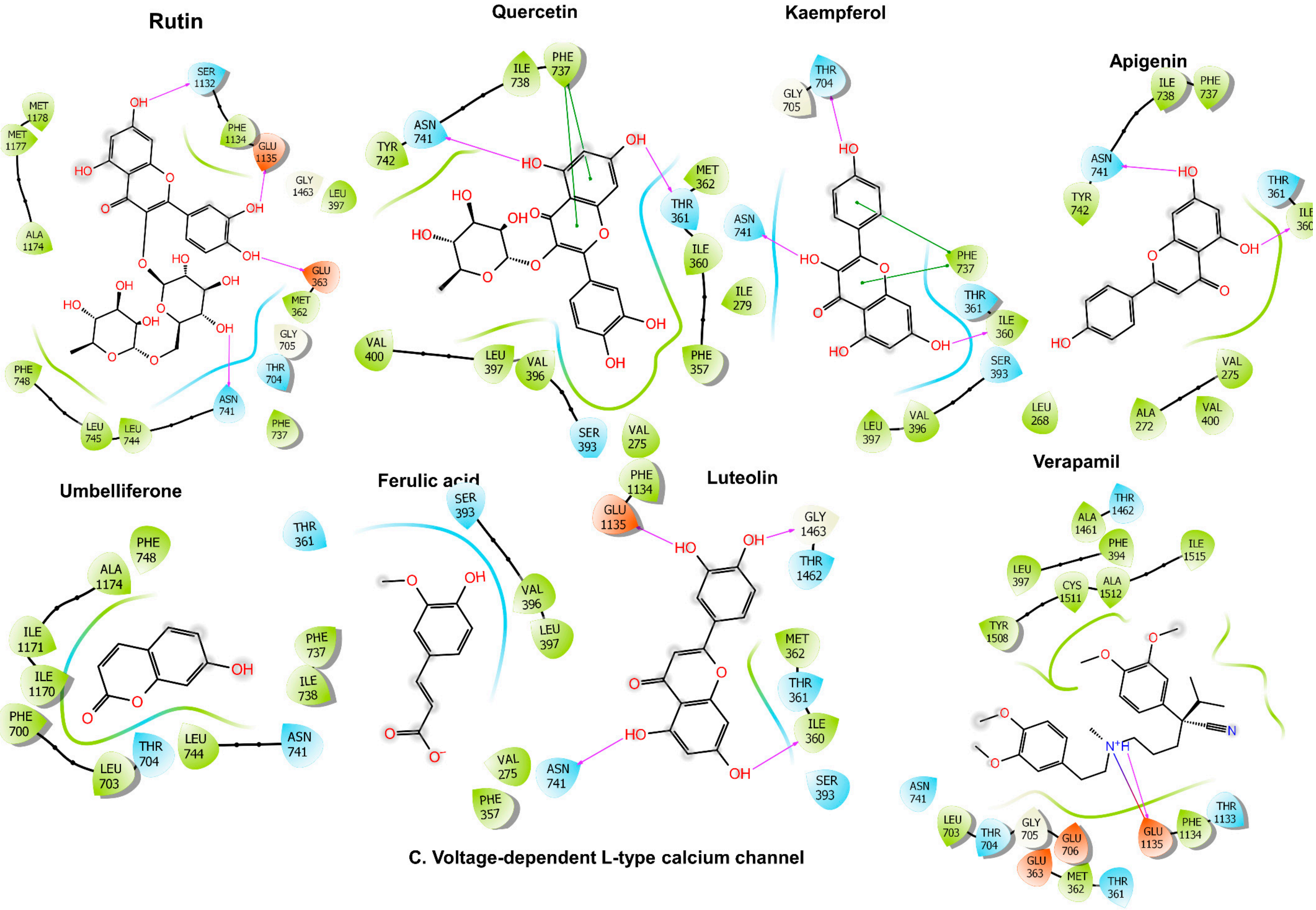

C. Voltage-dependent L-type calcium channel

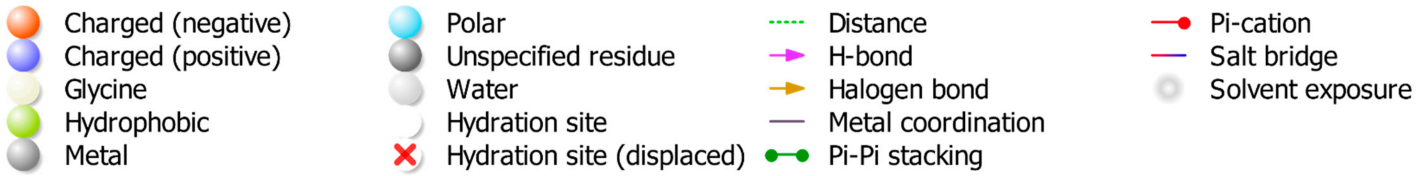

Figure S7. 2D ligand-protein interaction between bioactive compounds of *C. melo* seed sequential extracts and proteins; **A.** Interleukin 2 Receptor Subunit β (IL2RB) **B.** Nitric Oxide Synthase 2 (NOS2) **C.** Voltage-dependent L–type calcium channel subunit alpha 1C *α*.

Table S1. Identification of phytoconstituents of *C. melo* seeds extracts with LC ESI-MS/MS.

Extract	Molecular Weight	Observed MS (*m*/*z*)	Calculated MS (*m*/*z*)	Error	Precursor type	ESI–IT MS/MS (Ions)	Empirical Formula	Proposed compound	Class
Hexane	278.4	279.3318	279.3312	–2.15	$[M–H]^-$	279.08, 261.08, 247.08,	$C_{18}H_{30}O_2$	Linoleic acid	Polyunsaturated fatty acid; omega–6
	228.37	227.2014	227.2019	2.20	$[M–H]^-$	228.25, 227.25, 136.25	$C_{14}H_{28}O_2$	myristic acid	Fatty acids
	256.42	255.2327	255.2319	–3.13	$[M–H]^-$	255, 237, 223, 217, 181	$C_{16}H_{32}O_2$	Palmitic acid	Fatty acids
DCM	414.7	413.3785	413.3777	–1.94	$[M–H]^-$	412.08, 311.25, 297, 285.08, 255.08, 236.08, 171, 119	$C_{29}H_{50}O$	β–sitosterol	Phytosterols
	412.69	413.8455	413.8463	1.93	$[M+H]^+$	397, 379, 355, 342.1, 297,	$C_{29}H_{48}O$	Stigmasterol	Sterol
	162.14	161.0249	161.0247	–1.24	$[M–H]^-$	161, 133, 117,	$C_9H_6O_3$	Umbelliferone	Hydroxycoumarins
	430.71	431.3882	431.3879	–0.70	$[M+H]^+$	432,165, 136,	$C_{29}H_{50}O_2$	*α*–tocopherol	Fat-soluble vitamin
Ethanol	448.38	445.0979	445.0970	–2.03	[M–H]–	445.25, 417.08, 327.08, 297	$C_{21}H_{20}O_{11}$	Orientin	Flavone glucoside
	516.4	515.0226	515.0214	–2.33	[M–H]–	471, 353, 335, 317, 299.25, 179	$C_{25}H_{24}O_{12}$	1,4–Dicaffeoylquinic acid	Quinic acids
	270.24	269.0462	269.0458	–1.49	[M–H]–	269, 201, 151, 117	$C_{15}H_{10}O_5$	Apigenin	Flavonoids
	180.16	179.0361	179.0358	–1.68	[M–H]–	179, 135, 107	$C_9H_8O_4$	Caffeic acid	Hydroxycinnamic acids
	194.18	193.0506	193.0504	–1.04	$[M–H]^-$	193, 178, 164, 133, 108	$C_{10}H_{10}O_4$	Ferulic acid	Cinnamic acids
	134.09	133.0141	133.0137	–3.01	$[M–H]^-$	133, 115, 71	$C_4H_6O_5$	Malic acid	Carboxylic acid
	272.25	271.0628	271.0636	2.95	$[M–H]^-$	271, 177, 151, 119,	$C_{15}H_{12}O_5$	Naringenin	Flavanone
	302.23	301.0351	301.0346	–1.66	$[M–H]^-$	301.08, 271, 179, 151,119	$C_{15}H_{10}O_7$	Quercetin	Flavonoid
	610.5	609.1456	609.1454	–0.33	$[M–H]^-$	609.25, 301.08, 299, 271, 255, 178	$C_{27}H_{30}O_{16}$	Rutin	Flavonoid glycosides
	178.18	383.4023	383.4008	–3.91	$[M–H]^-$	177, 135,119	$C_{10}H_{10}O_3$	4–Methoxycinnamic acid	Cinnamic acids
	192.17	191.0346	191.0353	3.66	$[M–H]^-$	192, 148, 120, 104	$C_{10}H_8O_4$	Scopoletin	Hydroxycoumarins
	290.27	289.0714	289.0716	0.69	$[M–H]^-$	290, 271, 205, 179 161	$C_{15}H_{14}O_6$	Epicatechin	Catechin/ Flavonoids
	432.38	431.0980	431.0986	1.39	$[M–H]^-$	431, 285, 255, 227	$C_{21}H_{20}O_{10}$	kaempferol–3–*O*–rhamnoside	Flavonoid glycosides

	463.4	463.0867	463.0887	4.32	$[M–H]^-$	463, 301, 271	$C_{21}H_{19}O_{12}$	Quercetin–3–*O*–glucoside	Flavonoid
Aqueous	610.6	609.1811	609.1806	–0.82	$[M–H]^-$	609, 301	$C_{28}H_{34}O_{15}$	Hesperidin	Flavonoid glycosides
	286.23	285.0374	285.0392	6.31	$[M–H]^-$	285, 164,119	$C_{15}H_{10}O_6$	Kaempferol	Flavanols
	448.38	447.0959	447.0944	–3.35	$[M–H]^-$	447.09, 285, 255,227, 151	$C_{21}H_{20}O_{11}$	kaempferol–3–*O*–glucoside	Flavonoid glycosides
	286.24	285.0399	285.0402	1.05	$[M–H]^-$	285, 175, 152, 133, 107	$C_{15}H_{10}O_6$	Luteolin	Flavones
	448.37	447.0934	447.0927	–1.57	$[M–H]^-$	447, 285	$C_{21}H_{20}O_{11}$	Luteolin 7–*O*–glucoside	Flavonoid glycosides
	624.54	623.1626	623.1609	–2.73	$[M–H]^-$	623,315, 299,285,271	$C_{28}H_{32}O_{16}$	Narcissin/Isorhmnetin 3–*O*–rutinoside	Flavonoid glycosides
	164.16	163.0399	163.0404	3.07	$[M–H]^-$	163.0, 119, 93	$C_9H_8O_3$	*p*–coumaric acid	Hydroxycinnamic acids
	196.16	195.0506	195.0501	–2.56	$[M–H]^-$	195, 177, 129, 75	$C_6H_{12}O_7$	Gluconic Acid	Sugar acids and derivatives

Table S2. Precision validation of analytical method *C. melo* seed extracts

**Analytes**	**Theoretical concentration (µg ml $^{-1}$)**	**Inter-day precision (*n* = 5)**		**Intra-day precision (*n*=5)**	
		Measured concentration (µg ml $^{-1}$)	RSD %	Measured concentration (µg ml $^{-1}$)	RSD %
Stigmasterol	100	99.24±1.86	1.88	98.61±1.01	1.03
β-sitosterol	100	98.10±0.62	0.63	98.72±0.70	0.71
Umbelliferone	100	97.93±0.92	0.94	98.19±1.12	1.14
Caffeic acid	100	98.37±1.05	1.07	98.81±0.30	0.30
Rutin	100	98.06±1.23	1.25	97.98±1.46	1.49
Quercetin	100	98.77±1.67	1.69	98.65±1.27	1.28
Apigenin	100	98.80±1.27	1.29	98.88±1.60	1.62
Ferulic acid	100	98.76±1.61	1.63	98.61±1.31	1.33
1,4-Dicaffeoylquinic acid	100	98.40±0.88	0.90	98.75±1.73	1.75
Kaempferol	100	98.72±0.91	0.92	98.28±1.80	1.83
Luteolin	100	97.94±1.88	1.92	98.52±1.54	1.56
Hesperidin	100	98.66±0.92	0.93	98.59±1.15	1.17
Narcissin	100	97.91±1.09	1.12	98.32±2.21	2.25

All values are mean ± S.D. taken as triplicates. Percent coefficient of variation (% RSD); (SD/Mean) ×100.

Table S3. Accuracy validation of analytical method of *C. melo seed extracts* through % recovery method

Analytes	Standard additions µg/ml	% Recovery			Mean± SD	RSD%
		Day 1	Day 2	Day 3		
Stigmasterol	50	49.42	49.60	49.42	49.11±0.54	1.09
	100	98.05	96.89	99.04	98.26±0.84	0.86
	200	197.79	197.94	200.25	198.95±1.27	0.64
β-sitosterol	50	48.32	49.09	48.92	49.41±0.93	1.88
	100	96.82	98.43	101.03	99.34±1.70	1.71
	200	195.42	195.96	197.85	197.60±2.00	1.01
Umbelliferone	50	48.32	49.49	48.92	49.09±0.71	1.45
	100	96.82	98.97	98.03	98.65±1.28	1.30
	200	194.82	195.96	197.24	198.57±3.68	1.85
Caffeic acid	50	48.32	48.59	48.92	48.79±0.37	0.75
	100	97.81	98.97	99.03	99.02±0.75	0.76
	200	194.82	194.97	197.24	198.37±3.88	1.95
Rutin	50	50.30	48.86	50.92	50.16±0.79	1.57
	100	98.80	98.43	100.03	99.31±0.67	0.67
	200	197.79	197.08	200.25	198.78±1.48	0.75
Quercetin	50	48.32	49.19	48.92	49.43±0.92	1.87
	100	98.82	98.43	100.05	99.60±0.98	0.99
	200	195.81	195.96	198.25	197.76±1.91	0.97
Apigenin	50	48.32	49.49	48.92	49.08±0.51	1.04
	100	97.81	98.97	99.03	98.85±0.93	0.94
	200	194.82	197.94	197.24	197.76±2.02	1.02
Ferulic acid	50	49.30	49.49	49.91	49.88±0.52	1.04
	100	98.80	97.63	100.03	99.38±1.14	1.14
	200	195.82	197.94	199.25	198.36±1.70	0.86
1,4-Dicaffeoylquinic acid	50	47.75	48.50	49.34	48.86±0.75	1.54
	100	98.31	98.97	99.54	99.25±0.70	0.71
	200	197.19	197.95	200.64	198.92±1.36	0.68
Kaempferol	50	48.51	48.50	49.11	48.99±1.00	2.03
	100	97.22	97.98	98.43	98.72±1.25	1.27
	200	196.33	196.95	198.77	198.63±2.21	1.11
Luteolin	50	48.51	49.48	49.11	49.03±0.39	0.80
	100	98.00	98.97	99.22	99.16±0.77	0.78
	200	195.67	197.94	198.10	198.77±2.37	1.19
Hesperidin	50	48.90	49.05	49.51	49.72±0.88	1.78
	100	98.33	97.98	99.55	99.15±0.94	0.95
	200	196.99	200.91	199.44	199.27±1.43	0.72
Narcissin	50	48.51	49.49	49.11	49.76±1.06	2.13
	100	99.66	97.98	97.87	98.97±0.98	0.99
	200	196.99	194.98	199.24	198.34±2.32	1.17

All values are mean ± S.D. taken as triplicates. Percent relative of SD variation (% RSD); (SD/Mean) ×100.

Table S4. Network analysis of pathogenic target genes interaction with phytoconstituents

Name	Type	Betweenness Centrality	Closeness Centrality	Degree
PIK3R1	Gene	0.053844436	0.324324324	5
Quercetin	Ethanol	0.148531949	0.380952381	6
Apigenin	Ethanol	0.226053803	0.380952381	6
Kaempferol	Aqueous	0.078000548	0.358208955	5
Luteolin	Aqueous	0.078000548	0.358208955	5
naringenin	Ethanol	0.010174021	0.275862069	3
NOS2	Gene	0.435628019	0.333333333	5
Narcissin	Aqueous	0.00102657	0.258064516	2
Stigmasterol	DCM	0.079830918	0.311688312	2
Ferulic acid	Ethanol	0.334057971	0.369230769	2
Linoleic acid	Hexane	0.22826087	0.269662921	2
Palmitic acid	Hexane	0.008091787	0.311688312	2
PTGDR2	Gene	0.163043478	0.222222222	3
Sitosterol	DCM	0	0.252631579	1
Oleic acid	Hexane	0	0.252631579	1
ALOX15	Gene	0.398168108	0.413793103	6
Caffeic acid	Ethanol	0	0.183206107	1
IGF1R	Gene	0.0854269	0.333333333	6
1,4-Dicaffeoylquinic acid	Ethanol	0	0.183206107	1
ABCC1	Gene	0.072443808	0.333333333	6
IL2RB	Gene	0.10034219	0.307692308	5
Rutin	Ethanol	0	0.237623762	1
CACNA1A	Gene	0.086755233	0.307692308	3
Rutin	Ethanol	0	0.237623762	1
TBXAS1	Gene	0.100724638	0.342857143	2

Table S5. The binding energies (kcal/mol) of docked ligand-protein complex calculated with Prime MM–GBSA

Compounds	Docking score (kcal/mol)	Glide energy kcal/mol)	ΔG Bind (kcal/mol)	pKi (μM)	ΔG Coulomb (kcal/mol)	ΔG Covalent (kcal/mol)	ΔG Hbond (kcal/mol)	ΔG Lipo (kcal/mol)	ΔG Packing (kcal/mol)	ΔG Solv GB (kcal/mol)	ΔG vdW (kcal/mol)	Residue–Ligand Interactions with Distance (Å)	
												Hydrogen Bonds	Electrostatic / Hydrophobic Bonds
Interleukin 2 Receptor Subunit β (IL2RB)													
Rutin	–6.82	–41.93	–36.20	–12.49	–26.81	7.07	–2.39	–8.03	–1.17	20.33	–25.23	Conventional hydrogen bond: Asn87 (1.78 Å), Asn43 (1.85 Å), Asn43 (1.90 Å), Arg131 (1.60 Å), Carbon hydrogen bond: Ser45 (2.49 Å), Asn87 (2.56 Å), Asn43 (3.03 Å)	Pi–Cation; Pi–Donor Hydrogen Bond: Arg131 (4.03 Å), Pi–Sulfur: Met133 (4.25 Å), Pi–Alkyl: Pro222 (4.91 Å), Pro222 (4.88 Å), Arg131 (5.47 Å),
Luteolin	–6.22	–35.82	–38.97	–13.70	–26.40	6.22	–2.36	–6.75	–1.93	16.28	–24.03	Conventional hydrogen bond: Arg131 (1.62 Å), Phe37 (2.33 Å), Carbon hydrogen bond: Met133 (2.88 Å), Ala134 (1.84 Å), Arg131 (2.32 Å),	Pi–Sigma: Pro222 (2.61 Å), Pi–Cation: Arg131 (4.30 Å), Pi–Sulfur: Met133 (4.22 Å), Pi–Alkyl: Met133 (5.06 Å), Pro222 (4.45 Å),
Quercetin	–3.85	–42.28	–28.68	–9.23	–64.90	6.58	–2.25	–7.41	–3.05	71.39	–29.05	Conventional hydrogen bond: Ser45 (2.91 Å), Asn87 (1.91 Å), Arg131 (2.18 Å), Arg131 (2.54 Å), Phe37 (3.05 Å), Arg131 (3.10 Å), Asn43 (2.41 Å),	Pi–Sigma: Pro222 (2.72 Å), Pi–Sulfur: Met133 (5.76 Å), Alkyl: Val47 (4.63 Å), Pi–Alkyl: Ala134 (4.67 Å), Pro222 (4.34 Å), Met133 (5.12 Å),

												Carbon hydrogen bond: Ser45 (2.67 Å),	
Umbelliferone	–3.52	–26.52	–35.59	–12.23	–17.79	1.15	–1.10	–6.93	–2.01	11.39	–20.30	Conventional hydrogen bond: Ala134 (2.17 Å), Arg131 (1.64 Å), Carbon hydrogen bond: Pro222 (3.07 Å),	Pi–Cation: Arg131 (4.57 Å), Pi–Sulfur: Met133 (3.73 Å), Met133 (3.77 Å), Pi–Alkyl: Pro222 (4.87 Å),
Kaempferol	–3.40	–31.55	–30.08	–9.83	–22.87	7.41	–1.81	–5.41	–1.08	18.17	–24.49	Conventional hydrogen bond: Arg131 (2.05 Å), Arg131 (1.71 Å), Trp223 (2.37 Å), Carbon hydrogen bond: Ser45 (2.60 Å),	Pi–Cation: Arg131 (4.46 Å), Pi–Sulfur: Met133 (4.24 Å), Met133 (3.98 Å), Pi–Alkyl: Pro222 (5.35 Å), Ala134 (3.71 Å), Pro222 (4.56 Å),
Ferulic acid	–3.27	–22.50	–20.23	–5.56	–8.78	1.25	–1.12	–8.48	–0.97	18.90	–21.03	Conventional hydrogen bond: Arg131 (2.25 Å), Arg131 (1.91 Å), Carbon hydrogen bond: Phe37 (2.48 Å), Asn43 (2.74 Å), Asn43 (2.58 Å), Phe37 (2.73 Å),	Pi–Cation: Arg131pi–Sulfur: Met133 (3.88 Å),
Apigenin	–2.97	–30.28	–28.44	–9.12	–23.70	9.14	–1.81	–4.56	–1.09	18.86	–25.27	Conventional hydrogen bond Arg131 (2.06 Å), Arg131 (1.72 Å), Trp223 (2.39 Å), Carbon hydrogen bond: Ser45 (2.62 Å),	Pi–Cation: Arg131 (4.46 Å), Pi–Sulfur: Met133 (4.24 Å), Met133 (3.99 Å), Pi–Alkyl: Pro222 (5.32 Å), Ala134 (3.71 Å), Pro222 (4.56 Å),
Verapamil	–1.60	–37.75	–46.89	–17.13	–2.41	2.52	–1.10	–17.30	–1.00	9.31	–36.91	Conventional hydrogen bond: Ser45	Pi–Cation; Pi–Donor Hydrogen Bond: Arg131

												(2.76 Å), Arg131 (2.03 Å), Carbon hydrogen bond: Ser45 (2.62 Å), Ser45 (2.69 Å), Asn43 (2.50 Å), Phe37 (2.55 Å), Asn43 (2.68 Å), Phe37 (2.72 Å), Arg131 (2.61 Å),	(4.13 Å), Pi–Sigma: Val79 (2.94 Å), Pi–Sulfur: Met133 (4.45 Å), Alkyl: Val47 (4.59 Å), Val79 (4.57 Å), Val79 (4.78 Å), Pi–Alkyl: Ala85 (3.89 Å),
Nitric Oxide Synthase 2 (NOS2)													
Rutin	–14.69	–75.74	–18.93	–4.99	–27.16	23.80	–4.77	–18.44	–9.78	74.07	–56.65	Conventional hydrogen bond: Ile201 (1.92 Å), Glu377 (2.07 Å), Pro350 (2.61 Å), Cys200 (3.03 Å), Arg199 (1.63 Å), Asn370 (1.84 Å), Arg199 (2.99 Å), Carbon hydrogen bond: Gly371 (2.95 Å), Glu377 (2.55 Å), Arg199 (2.51 Å), Arg199 (2.98 Å), Trp463 (2.56 Å),	Pi–Anion: Cys200 (3.87 Å), Pi–Pi Stacked: Trp194 (4.22 Å), Trp194 (3.65 Å), Trp194 (5.32 Å), Trp194 (4.13 Å), Phe369 (4.34 Å), Phe369 (4.14 Å), Pi–Alkyl: Cys200 (4.21 Å), Ala197 (4.32 Å), Cys200 (4.66 Å),
Quercetin	–10.50	–58.68	–42.09	–15.05	–37.64	8.23	–2.24	–15.54	–4.90	54.53	–44.52	Conventional hydrogen bond: Tyr491 (2.45 Å), Glu377 (2.32 Å), Glu377 (2.16 Å), Tyr489 (1.79 Å), Cys200 (2.14 Å), Carbon hydrogen bond: Arg199 (2.65 Å), Glu377 (2.85 Å),	Pi–Sigma: Met355 (2.45 Å), Pi–Sulfur: Met355 (4.27 Å), Met355 (4.41 Å), Pi–Pi Stacked: Trp194 (4.29 Å), Phe369 (3.93 Å), Pi–Pi T–Shaped: Tyr491 (5.55 Å), Pi–Alkyl: Ala197 (4.42 Å), Arg199 (5.23 Å), Cys200 (5.44 Å),

													Ala197 (4.78 Å), Arg199 (4.40 Å), Ala197 (4.79 Å), Cys200 (4.47 Å),
Luteolin	−9.09	−40.07	−47.68	−17.48	−41.19	6.01	−1.01	−12.68	−4.92	38.94	−32.84	Conventional hydrogen bond: Tyr491 (2.44 Å), Tyr489 (1.81 Å), Cys200 (2.01 Å), Carbon hydrogen bond: Arg199 (2.63 Å),	Pi–Sulfur: Met355 (4.27 Å), Met355 (4.41 Å), Pi–Pi Stacked: Trp194 (4.31 Å), Phe369 (3.98 Å), Pi–Pi T–Shaped: Tyr491 (5.47 Å), Pi–Alkyl: Ala197 (4.38 Å), Arg199 (5.19 Å), Ala197 (4.86 Å), Arg199 (4.39 Å), Ala197 (4.73 Å), Cys200 (4.41 Å),
Kaempferol	−8.14	−39.60	−25.84	−7.99	−16.63	2.14	−0.75	−10.09	−9.93	46.22	−36.79	Conventional hydrogen bond: Ser242 (1.95 Å), Asn370 (2.30 Å), Carbon hydrogen bond: Gly371 (2.78 Å),	Pi–Donor Hydrogen Bond: Tyr489 (3.07 Å), Trp372 (2.38 Å), Pi–Pi Stacked: Trp194 (3.53 Å), Trp194 (3.92 Å), Trp194 (3.94 Å), Trp194 (5.27 Å), Phe369 (3.94 Å), Pi–Pi T–Shaped: Trp372 (5.07 Å), Trp372 (5.53 Å), Pi–Alkyl: Cys200 (4.91 Å), Ala197 (5.20 Å), Cys200 (4.60 Å),
Apigenin	−7.98	−36.73	−38.28	−13.40	−27.47	4.65	−0.85	−13.17	−4.97	37.56	−34.03	Conventional hydrogen bond: Tyr491 (2.61 Å), Tyr489 (1.79 Å), Carbon hydrogen bond: Arg199 (2.67 Å),	Pi–Donor Hydrogen Bond: Trp194 (2.89 Å), Pi–Sigma: Met355 (2.46 Å), Pi–Sulfur: Met355 (4.32 Å), Met355 (4.38 Å), Pi–Pi Stacked:

													Trp194 (4.21 Å), Trp194 (5.69 Å), Phe369 (3.89 Å), Pi–Pi T–Shaped: Tyr491 (5.62 Å), Pi–Alkyl: Ala197 (4.31 Å), Arg199 (5.32 Å), Cys200 (5.34 Å), Ala197 (4.73 Å), Arg199 (4.41 Å), Ala197 (4.83 Å), Cys200 (4.57 Å),
Umbelliferone	−6.83	−25.99	−28.30	−9.06	−0.91	3.78	−0.96	−17.83	−10.04	21.05	−23.40	Conventional hydrogen bond: Asn370 (1.73 Å),	Pi–Pi Stacked: Trp194 (4.04 Å), Trp194 (3.58 Å), Trp194 (5.43 Å), Trp194 (3.92 Å), Phe369 (3.80 Å), Phe369 (4.46 Å), Pi–Alkyl: Ala197 (5.08 Å), Cys200 (4.77 Å),
Verapamil	−6.08	−55.87	−60.12	−22.88	−27.31	4.87	−1.57	−34.44	−4.91	61.88	−58.63	Salt Bridge;Attractive Charge: Glu377 (2.50 Å), Hydrogen Bond: Trp372 (2.79 Å), Carbon hydrogen bond: Arg199 (3.07 Å), Glu377 (2.62 Å), Tyr373 (3.02 Å), Trp372 (2.80 Å),	Pi–Sigma: Gly202 (2.59 Å), Pi–Sulfur: Cys200 (3.99 Å), Pi–Pi T–Shaped: Phe369 (4.51 Å), Trp372 (5.53 Å), Alkyl: Met374 (4.92 Å), Met434 (3.95 Å), Pi–Alkyl: Trp194 (4.06 Å), Trp194 (3.66 Å), Trp194 (5.26 Å), Trp194 (3.58 Å), Trp194 (5.02 Å), Phe369 (3.51 Å), Trp372 (4.19 Å), Trp372 (4.16 Å), Trp372 (3.62 Å),

												Tyr489 (4.38 Å), Cys200 (5.19 Å),
Ferulic acid	–5.95	–26.82	–8.19	–0.33	28.26	1.88	–0.19	–20.51	–5.24	20.77	–33.16	Conventional hydrogen bond: Ser242 (2.73 Å),	Pi–Pi Stacked: Trp194 (3.49 Å), Trp194 (4.08 Å), Phe369 (4.55 Å), Alkyl: Leu209 (4.53 Å), Ile244 (4.44 Å), Pi–Alkyl: Trp194 (4.94 Å), Phe369 (4.29 Å), Tyr489 (4.49 Å), Cys200 (5.15 Å),
Voltage-dependent L–type calcium channel subunit alpha–1C (VGCAC1C)													
Rutin	–9.07	–57.33	–20.14	–5.52	–58.00	10.83	–2.79	–14.40	–0.41	89.69	–45.06	Conventional hydrogen bond: Asn741 (1.69 Å), Ser1132 (1.73 Å), Glu1135 (2.21 Å), Gly1463 (3.00 Å), Glu363 (1.96 Å), Carbon hydrogen bond: Gly705 (2.98 Å), Asn741 (2.99 Å), Asn741 (2.79 Å),	Alkyl: Leu744 (4.82 Å), Leu745 (4.44 Å), Met1178 (5.29 Å), Pi–Alkyl: Phe748 (5.44 Å),
Quercetin	–7.58	–48.23	–40.37	–14.30	–5.50	6.64	–1.05	–16.75	–1.00	25.22	–47.92	Conventional hydrogen bond Ile738 (2.57 Å), Asn741 (1.92 Å), Thr361 (1.90 Å), Carbon hydrogen bond: Ile738 (2.86 Å),	Pi–Pi T–Shaped: Phe737 (5.24 Å), Phe737 (5.29 Å), Alkyl: Ile738 (4.12 Å), Pi–Alkyl: Leu397 (4.87 Å), Ile360 (4.71 Å), Val396 (5.33 Å),
Apigenin	–6.12	–32.47	–24.50	–7.41	–11.43	0.24	–0.78	–8.01	–0.24	25.42	–29.70	Conventional hydrogen bond: Ile360 (2.04 Å), Asn741 (2.71 Å),	Pi–Donor Hydrogen Bond: Tyr742 (3.29 Å), Pi–Alkyl: Val275 (5.46 Å), Val400 (5.07 Å),

												Ala272 (5.42 Å), Val400 (4.33 Å),
Luteolin	–5.78	–42.83	–16.03	–3.73	–34.43	5.30	–2.57	–3.71	–0.94	49.26	–28.94	Conventional hydrogen bond: Asn741 (2.11 Å), Ile360 (1.84 Å), Glu1135 (1.90 Å), Glu1135 (2.55 Å), Thr1462 (3.04 Å), Gly1463 (1.98 Å),	Pi–Alkyl: Leu397 (4.90 Å), Met362 (5.05 Å),
Verapamil	–5.77	–56.89	–25.64	–7.91	–89.04	2.95	–0.81	–18.14	–0.67	128.72	–48.66	Salt Bridge;Attractive Charge: Glu363 (3.12 Å), Glu1135 (2.41 Å), Attractive Charge: Glu706 (5.02 Å), Carbon hydrogen bond: Glu363 (2.60 Å), Glu1135 (2.71 Å), Thr704 (2.49 Å), Glu363 (2.65 Å), Glu1135 (2.73 Å), Asn741 (2.59 Å), Ile360 (2.69 Å), Ser1132 (2.80 Å), Ser1132 (2.86 Å),	Pi–Pi T–Shaped: Phe737 (5.70 Å), Tyr1508 (5.67 Å), Alkyl: Ala1512 (3.85 Å), Met362 (5.13 Å), Pi–Alkyl: Phe737 (4.51 Å), Phe1134 (4.76 Å), Tyr1508 (5.15 Å), Leu397 (4.71 Å),
Kaempferol	–5.75	–35.56	–30.99	–10.23	–40.42	3.84	–1.59	–6.27	–1.29	36.70	–21.96	Conventional hydrogen bond: Asn741 (1.63 Å), Ile360 (1.80 Å), Thr704 (1.74 Å),	Pi–Pi T–Shaped: Phe737 (5.49 Å), Phe737 (5.20 Å), Pi–Alkyl: Leu397 (4.55 Å), Leu397 (4.59 Å),
Umbelliferone	–4.57	–23.01	–25.39	–7.80	–4.01	0.59	–0.24	–11.97	–1.08	15.09	–23.76	Conventional hydrogen bond: Asn741 (2.86 Å), Carbon hydrogen bond: Thr704 (2.79 Å),	Pi–Alkyl: Leu744 (4.38 Å), Ala1174 (5.01 Å), Leu744 (4.24 Å), Ala1174 (5.25 Å),

Ferulic acid	–3.85	–17.57	–10.73	–1.43	15.48	2.68	0.00	–11.80	0.00	5.66	–22.75	Carbon hydrogen bond: Ser393 (2.43 Å),	Pi–Alkyl: Phe737 (4.68 Å), Leu397 (4.70 Å),

Values are expressed as the mean ± S.D., $n = 3$. ΔGBinding: Binding free energy; pKi: Logarithmic of Inhibition Constant (Ki); ΔGCoulomb: Coulomb binding energy; ΔGCovalent: Covalent binding energy; ΔG.H.: Hydrogen bonding energy; ΔGLipophilic: Lipophilic binding energy; ΔGSolv GB: Generalized born electrostatic solvation energy; ΔGvdW: Van der Waals forces energy; These energies all contribute to Binding free energy (ΔGBinding).
